# Distinctive signatures and ultrafast dynamics of Brønsted sites, silanol nests and adsorbed water in zeolites revealed by 2D-IR spectroscopy†

**DOI:** 10.1039/d4sc08093a

**Published:** 2025-03-25

**Authors:** Paul M. Donaldson, Alexander P. Hawkins, Russell F. Howe

**Affiliations:** a Central Laser Facility, Research Complex at Harwell, STFC Rutherford Appleton Laboratory Harwell Science and Innovation Campus, Didcot OX11 0QX UK paul.donaldson@stfc.ac.uk; b Department of Chemistry, University of Aberdeen Aberdeen AB24 3UE UK

## Abstract

Characterising hydroxyl groups in zeolites and other amorphous solids often relies on methods such as IR and NMR spectroscopy. Their power to distinguish different types of hydroxyl groups diminishes when band broadening from hydrogen bonding and structural heterogeneity occurs. In support of this problem, we report *in situ* femtosecond 2D-IR spectroscopy of some of the different types of hydroxyl groups present in zeolites. Despite the samples studied being optically scattering pellets, we show that their structural and rotational dynamics can be determined. We show that the hydroxyl groups of Brønsted acid sites, silanol defects and water of hydration display distinct features in their 2D-IR spectra. Brønsted site hydroxyl group structural distributions have characteristic inhomogeneously broadened 2D-IR bandshapes. Water of hydration and partially hydrogen bonded silanol groups give unique 2D-IR cross peak signatures off-diagonal. Hydrogen bonded silanol groups arising from vacancy defects (silanol nests) show a distinctive 2D-IR signature with unique ultrafast dynamics observed to be identical between ZSM-5 and silicalite-1. 2D-IR spectroscopy makes IR measurements quantitative, and we use this property to estimate the concentration of ZSM-5 silanol nest hydroxyl groups relative to the number of Brønsted sites. Overlapping silanol nest spectral features are revealed by frequency dependence of their vibrational lifetime. In contrast to other framework hydroxyls, the silanol nest band shows picosecond 2D-IR anisotropy decay and spectral diffusion. The signatures of nest structural mobility revealed here presents new opportunities to understand these hitherto elusive structural defects.

## Abstract 2

Exploring the information rich structural and dynamical landscape of the vibrations of zeolite hydration water, Brønsted sites and silanol nest vacancy defects using ultrafast 2D-IR spectroscopy.

## Graphical abstract

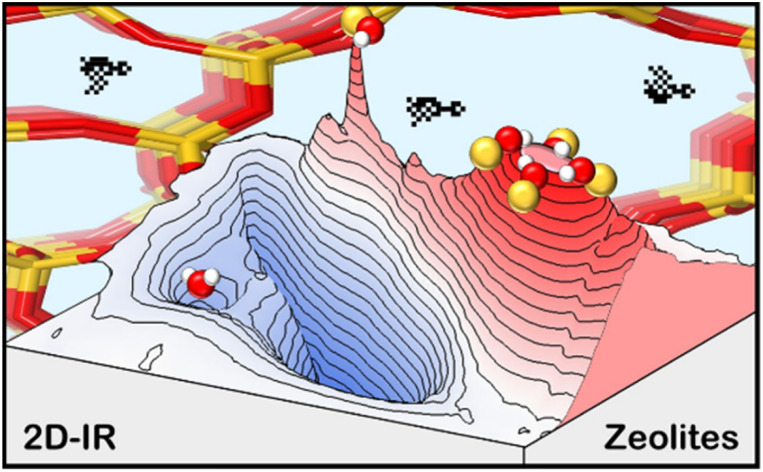

## 1. Introduction

Siliceous materials play important roles in many scientifically and industrially important research areas such as catalysis, chemical purification, materials science and geology. Much of the molecular-scale knowledge gathered about these materials comes from physical characterisation methods involving diffraction, imaging and spectroscopy. The heterogeneous and disordered character of many zeolites makes spectroscopic methods such as infrared (IR) and Nuclear Magnetic Resonance (NMR) especially important tools. Here, in the context of IR spectroscopy we examine zeolites: an important class of aluminosilicate materials ubiquitous as catalysts. The diversity of zeolite hydroxyl group types are readily observable in IR and NMR spectra,^1^ however spectral congestion and overlap with bound water can be an obstacle to fully understanding hydroxyl composition. This paper applies ultrafast laser multidimensional (2D)-IR spectroscopy measurements in support of the problem. In particular we explore the use of 2D-IR spectroscopy to distinguish different zeolite Brønsted sites, silanol defects and bound water, and we demonstrate 2D-IR's ability to determine relative concentrations of hydroxyl species. We show conclusively that one particular commercial ZSM-5 zeolite studied contains high concentrations of silanol defect nests with identical 2D-IR waiting time dynamics to those of silanol nests in an aluminium-free silicalite. Such defects are hardly present in a different ZSM-5 sample or in a ferrierite zeolite.

**Fig. 1:**
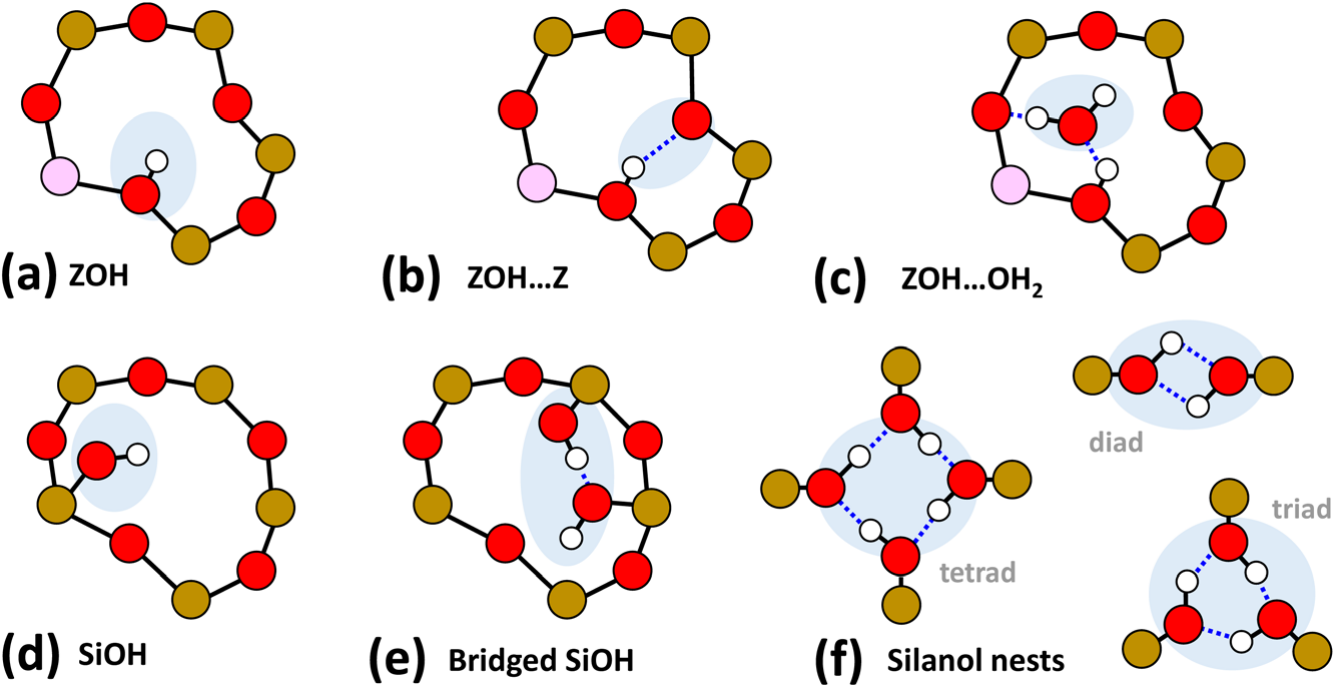
Hydroxyl groups in zeolites. (a)–(c) all involve the Brønsted site. Defect silanol groups in zeolites are categorised into (d) free silanols, (e) bridged silanols and (f) silanol nests. Red = O, brown = Si, pink = Al and white = H. Several further structures involving hydroxyl interactions with anionic SiO^−^ are described by Medeiros-Costa *et al.*^1^

Of the numerous forms of hydroxyl species in zeolites, the primary type of hydroxyl group present in a fully dehydrated acid zeolite are Brønsted acid sites, comprising protons bonded to oxide ions bridging silicon and aluminium atoms, formally Si(OH)Al. We denote these as ‘ZOH’, and ‘ZOD’ for the deuterated form (Fig. 1(a)). Brønsted acid sites may interact with the zeolite framework^2^ (ZOH⋯Z, Fig. 1(b)) and may be hydrogen bonded to water (ZOH⋯OH_2_, Fig. 1(c)). Nearly always present in ZSM-5 are silanol (SiOH) groups associated with internal defects or the external surface of the zeolite (Fig. 1(d)). These arise at crystal boundaries and faults (connectivity defects) or at individual lattice sites (vacancy defects). Fig. 1 depicts a hierarchy of some of these silanol defects ranging from ‘free’ (non-hydrogen bonded), *e.g.*Fig. 1(d), to bridged silanols 1(e) and nests 1(f). A variety of other defects involving silanols, oxide ions and impurity cations have been hypothesised and studied.^1,3,4^

In this paper, we envisage the term ‘nest’ to refer to fully hydrogen bonded silanol vacancy defects, however the definition continues to be the subject of debate. The term describes defects created by removal of an Al atom from an internal site of a zeolite, leaving a vacancy of four oxygens in the framework made chemically stable through termination of each oxygen with hydrogens.^5^ The four hydroxyl groups are often depicted hydrogen bonded, giving the characteristic silanol nest motif depicted in Fig. 1(f) (tetrad). Questions arise as to whether tetrad silanol nest defects are at all stable,^6^ whether the hydroxyl groups are hydrogen bonded equally in strength,^4^ or whether nests are hydrogen bonded diads^3,7,8^ or triads.^1,4,9^ Accompanying these questions are difficulties in distinguishing silanol defects from one another or from weakly hydrogen bonded Brønsted sites with NMR or IR spectroscopy.^1,10^ As nests and other silanol defects modify the adsorption, reactivity and aging behaviour of commercially important zeolite catalysts, developing an understanding and control of their properties and structural distributions are an active area of zeolite research.^1^

This paper is concerned with IR spectroscopy, which measures the frequency dependent absorption of IR light by a sample's molecular vibrations. A weak, continuous light source is typically used in benchtop IR absorption spectrometers. Such instruments are therefore unable to directly access the sub-nanosecond (ns) timescales of molecular vibrational dynamics, nor readily distinguish different types of band broadening mechanisms. These constraints disappear when IR spectroscopy is performed with short IR laser pulses. Indeed, early picosecond IR laser pump–probe measurements were able to determine vibrational lifetimes of Brønsted hydroxyls in zeolites and quantify their inhomogeneous broadening.^11^ The more recent and more sophisticated femtosecond (fs) 2D-IR approach^12^ maps out the response of a sample to IR excitation in greater detail and with greater time resolution. The vibrational modes in a sample are usually excited by two intense, interfering pulses of IR light, as opposed to a single excitation pulse for pump–probe. The excited sample's evolution in time is probed by a further IR pulse after a waiting time period (defined here as ‘*t*_2_’).^13–16^ Although the chemical sensitivities and behaviours of nuclear spin and bond vibrations differ, the underlying approach of 2D-IR spectroscopy has much in common with 2D-NMR.^12^

The development of 2D-IR applications to microcrystalline and disordered solids is in its infancy. Owing to problems arising from optical scattering, 2D-IR studies of zeolites are few in the literature. 2D-IR spectra of water clusters in ZSM-5, and of Bronsted-sites hydrogen bonding to single water molecules were reported recently by Hack *et al.*,^17,18^ who quantified ZSM-5 water cluster hydrogen bond distributions and explained the unique shape of the hydrogen bonded Brønsted hydroxyl stretch IR spectrum as arising from a double-well proton potential. In these examples, the detrimental effect of intense laser scatter to recording a 2D-IR spectrum of the ZSM-5 particles was mitigated by dispersing them in index matching oil. Desiring to study zeolites under dynamic gas composition and temperature, we developed a transmission 2D-IR approach able to obtain spectra from compressed pellets without index matching oils.^19^ We reported 2D-IR spectra of the *ν*(OD) deuteroxyl stretch region of pelleted ZSM-5, zeolite Y, pyrogenic silica and P25 TiO_2_. For these samples, we observed 2D-IR spectroscopy to give *ν*(OD) spectra more unique than ‘1D’ IR absorption spectroscopy through the shape of the ‘diagonal’ 0 → 1 bleach and 1 → 2 excited state absorption (ESA) bands. This is because broadening due to structural inhomogeneities and broadening due to lifetime effects give different 2D-IR bandshapes.

Cross peaks from vibrational coupling are another key feature of 2D-IR measurements which report on structure and assist spectra-structure correlations. These were observed in the 2D-IR spectrum of clustered water in ZSM-5.^17^ We recently showed for the case of the *ν*(OD) bands of pyrogenic silica how vibrational coupling between hydrogen bonded and non-hydrogen bonded bridged silanol groups gives rise to distinctive cross peaks in the off-diagonal part of the 2D-IR spectrum.^20^ We demonstrate here how these effects support assignments of silanol nests and any form of hydration water. We also explore three further properties of 2D-IR which add considerable value to studies of zeolites and other siliceous materials. These are (i) the ability to determine timescales of structural dynamics through evolution of the diagonal 2D-IR bands with ultrafast ‘waiting time’ *t*_2_, (ii) determination of timescales of molecular rotational motion through the evolution of anisotropy with *t*_2_ and (iii) determination of concentrations of unknown spectral features relative to others in the spectrum. Being able to observe these effects *in situ* from optically scattering zeolite pellets is an important result.

This 2D-IR investigation focusses on characterising the Brønsted sites, hydration water and silanol nest defects of MFI zeolite ZSM-5. We make assignments by (i) exploring how the 2D-IR bands from the Brønsted sites and bound water behave in comparison to other zeolites and as a function of temperature, (ii) 2D-IR spectral differencing with reference to silicalite – considered to be a model compound for the silanol nest defect^21^ and (iii) close comparison of the ZSM-5 and silicalite 2D-IR waiting time dynamics. The identification of the silanol nest band in *ν*(OH) stretch IR absorption spectra of ZSM-5 has been discussed and explored elsewhere.^7,22^ Across the literature, ambiguities in interpretation of this band in ZSM-5 IR absorption spectra remain, with alternative assignments to Brønsted acid groups hydrogen bonded to the zeolite lattice or to residual adsorbed water often made.^23,24^

Spectral overlap is a common cited problem for both IR and solid state NMR,^10^ with solid state NMR argued as the better approach for silanol species characterisation.^25^ This 2D-IR investigation provides counterpoints to that conclusion. Vibrational lifetime measurements reveal that there are several distinct nest features in the samples studied, even at high temperatures where there appears by-eye to only be one band in the IR spectrum. Through cross peak evolution, spectral diffusion and anisotropy decays, we see evidence that the silanol nests are structurally dynamic on picosecond timescales. In studying hydroxyl and deuteroxyl stretch features by IR absorption spectroscopy, the challenge of determining concentrations arises. Given any mixture of molecular species, IR quantification of concentrations is only possible when the molar absorption coefficients of each component are known. By applying a spectrophotometric concentration ratio approach unique to pump–probe laser spectroscopies and 2D-IR^26^ we are able to quantify the ratio of ZSM-5 silanol nest deuteroxyl groups present relative to the Brønsted ZOD groups without knowledge of the absorption coefficients.

## 2. Materials and methods

### 2.1. Spectroscopy

2D-IR spectra were acquired at the CLF-ULTRA facility (>5 years of multiple visits). The spectrometer, techniques of scatter suppression, sample cells and determination of the pellet temperature have been described previously.^19^ A 50 fs 10 kHz Ti:sapphire-driven dual optical parametric amplifier (OPA) system making IR light through difference frequency mixing in AgGaS_2_ supplied IR pump and probe pulses of centre wavelength 2600 cm^−1^ and full width at half height spectral bandwidths of 350–450 cm^−1^ (probe) and 220–300 cm^−1^ (pump). The pump beam was passed through a germanium acousto-optic pulse shaper (Phasetech Spectroscopy) to make *t*_1_ time separated pulse-pairs. Over the different visits, instrument responses (Kerr effect) ranged from 80–120 fs. At the sample, incident pulse energies ranged between ∼0.5–1.2 μJ (probe) and ∼0.3–1 μJ (shaped pump light). Probe light incident on the sample was polarised at an angle of 45° to the pump light. Post-sample, the parallel and perpendicular components of the probe beam were separated with polarisers and dispersed in individual spectrometers onto two separate HgCdTe array detectors (Infrared Associates, 128 pixels) at ∼4 or ∼6 cm^−1^ spectral resolution per pixel depending on grating choice. In addition to 2D-IR, an FT-IR spectrometer (Bruker Tensor/Invenio) was operated adjacent to the 2D-IR spectrometer, allowing both types of spectra to be acquired under the same conditions.

For 2D-IR, we use labelling conventions^12^ of *t*_1_ for coherence time, waiting time *t*_2_ and frequencies *ω*_1_ (pump) and *ω*_3_ (probe). 2D-IR spectra were generated by Fourier Transform (FT) of *ω*_3_ spectral interferograms acquired using *t*_1_ scans in steps of 17–22 fs to final times of 3–4 ps, making the *ω*_1_ spectral resolution ∼4–5 cm^−1^. Signal averages of 100–2000s per waiting time were used to obtain data of the desired signal-to-noise and heterodyne scatter removal. The intensity of remaining homodyne scatter was often negligible for the Zeolyst ZSM-5 sample and larger for the ferrierite, zeolite X, Mobil ZSM-5 and silicalite samples. Subtractions of this scatter collected at long negative waiting times^27^ was not particularly reliable, due to laser noise and thermal grating effects (at early waiting times). In using tight focusing and the ‘bright probe’ approach, 5th order effects can appear in the 2D-IR spectra.^19,20^ Once identified in later sessions, we avoided <50 μm pump and probe focussing. Varying in strength between sessions^20^ (for example completely absent for the session leading to Fig. 3, 5 and ESI Section 9†), we are confident that 5th order effects do not affect the conclusions of this work.

### 2.2. Samples

Our choice of ZSM-5 sample was determined by commercial availability. Inherent to zeolites is the challenge that in addition to the framework type, the defect nature, crystal sizes and composition are dependent on the origins of the sample, including the batch number, synthesis, levels of trace metals, pre-treatment protocols and age. The samples were characterised by X-ray fluorescence (XRF) and NMR, as described in ESI Section 1,† and by powder X-ray diffraction and scanning electron microscopy. Ammonium form ZSM-5 (Si : Al ratio 24) was obtained as a powder from Zeolyst (CBV5524G, lot 2493-62). Unless noted, this is the ZSM-5 sample used for the investigations. A second sample of ZSM-5 (Si : Al ratio 24) synthesised by Mobil in the 1980s^28^ was also examined for comparisons with the Zeolyst sample. Ammonium-form ferrierite (Si : Al ratio 9) was obtained as a powder from TOSOH (series HSZ-700). All zeolites were converted to their proton form by calcination in air at 600 °C for 12 hours. The Na^+^-form of ZSM-5 was prepared by stirring the proton-form ZSM-5 in 2 M NaCl solution followed by repeated washing in water to remove the excess NaCl/HCl, then drying in an oven. 24 hours stirring at 70 °C was observed by IR spectroscopy to exchange all ZSM-5 Brønsted sites and remove >70% of the silanol nest band. 12 hours at 60 °C exchanged only the Brønsted sites. Silicalite-1 and zeolite X synthesised *via* standard published routes^29,30^ were kindly donated by Emma Campbell.

Pellets of each sample were made in a hydraulic press (Specac) using a 13 mm diameter die, 10–12 mg of material and a pressure of ∼2 tonnes applied for 1 minute. The self-supporting pellets were ∼80–100 μm in thickness and studied in two kinds of spectroscopy cells involving (a) direct contact with a heater block isolated from the cell housing (Linkam FTIR600, range −180 °C to 600 °C) or (b) placement between heated CaF_2_ windows and cell body (Harrick TFC-S25-3, range 2 °C to 220 °C). Owing to temperature gradients and laser heating, the heater block thermocouple temperatures were not the same as the sample temperature, requiring corrections to be made as described previously.^19^

The decision to deuterate the samples is historic and based on frequent operation of the laser and pulse-shaper in the OD-stretch region, and on the samples typically being less scattering at longer excitation wavelengths. For deuteration, the cells were operated at 1 atm pressure under flowing N_2_ carrying fixed dilutions (∼5–20% by flowrate) of D_2_O vapour obtained by sparging N_2_ through D_2_O in a Dreschel flask. Experiments were on free-standing zeolite pellets, as described above, or where noted, on thinner samples made by pressing smaller amounts of zeolite directly between CaF_2_ windows.

The free-standing pellets showed rapid H–D exchange times (<10 min at *T* > 220 °C), rapid sample equilibration times (<1 min for Δ*T* ∼ 50 °C) and rapid water re-uptake times (1–2 minutes upon cooling a hot-dehydrated sample in the driest possible stream of N_2_). Zeolites are extremely hygroscopic, but water re-uptake was found to be slowed significantly when the zeolite pellet was sandwiched between CaF_2_ windows. A hot-dehydrated zeolite or silicalite cooled rapidly to room temperature this way would take hours to reabsorb water from a stream of saturated vapour flowed through the cell. Purging the cell briefly with dry N_2_ extended water re-uptake times at the centre of the pellet to days and enabled studies at cryogenic temperatures not possible with free-standing pellets, no matter how dry the N_2_ stream. When sandwiched, H–D exchange was achieved in 10–15 minutes by cycling several times between (i) 50 and (ii) 250 °C in D_2_O/N_2_ vapor ((i) accelerating water uptake and (ii) accelerating exchange). We have not observed IR measurements conducted this way on zeolites in the literature and consider ‘sandwiching’ a very useful way for keeping zeolites dehydrated without applying a vacuum, for achieving much thinner samples than possible with a pellet press and for exploring diffusion of adsorbates through to the centre of the pellets.

## 3. ZSM-5 and ferrierite IR and 2D-IR spectra at high temperature

**Fig. 2:**
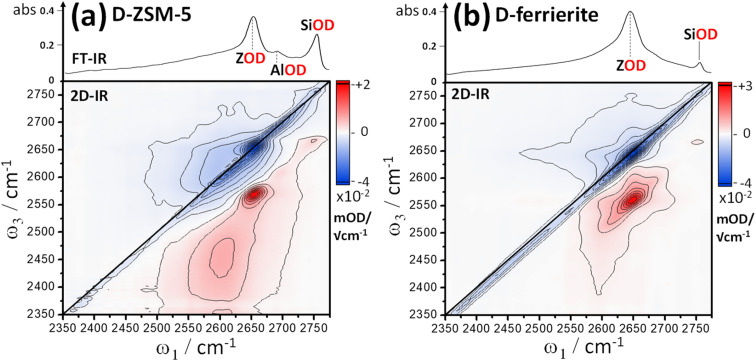
2D-IR spectra of deuterated pellets of ZSM-5 (a) and ferrierite (b) held at ∼240 °C in flowing D_2_O/N_2_ vapor. IR absorption spectra are shown at the top of the 2D spectra. Blue indicates negative signal (bleaching and stimulated emission), red indicates positive signal (excited state absorption, ESA). Crossed pump–probe polarisation (〈XXYY〉) and a waiting time of *t*_2_ = 300 fs were used.

Fig. 2 shows representative *ν*(OD)-stretch IR absorption and 2D-IR spectra measured from pressed pellets of ZSM-5 and ferrierite isotope exchanged at 240 °C in flowing, dilute D_2_O/N_2_ vapour. The IR absorption spectra match published *ν*(OH) region spectra of similar zeolites,^31^ with replacement of H by D reducing the stretch frequencies by a factor of ∼1.32–1.37.^32^ Despite being held in a hydrous vapour stream, at 240 °C, no water binds, as we will argue shortly. The IR spectra are therefore characteristic of dehydrated samples. The intense band at ∼2650 cm^−1^ is due to free Brønsted acid OD groups (ZOD, Fig. 1(a)). Both ZSM-5 and ferrierite show an IR band at 2756 cm^−1^ due to free SiOD groups (Fig. 1(d)) associated with defect and external surface sites. ZSM-5 shows a third weak feature at ∼2692 cm^−1^ attributable to extra-framework AlOD species (see ESI Section 1†).

One of the central features of discussion in this paper are the broad overlapping lower frequency bands present in the ZSM-5 and ferrierite IR spectra of Fig. 2. We shall return to these after discussing the 2D-IR spectra. We encourage the reader to look closely at the 2D-IR contours, and compare the features laid out in 2D with the IR absorption spectra directly above. On and around the diagonal of the 2D-IR spectra, the *ν*(OD) stretch features of the IR absorption spectra are evident as negative *v* = 0 → 1 bleach/stimulated emission signals (denoted here simply as ‘bleach’), coloured blue. The *ν*(OD) stretch groups also display excited state absorption (ESA, red) arising from *v* = 1 → 2 transitions, displaced from the diagonal by each mode's anharmonicity. Continuous signal in the region immediately on-diagonal (indicated by black lines in Fig. 2) is due to pump-light scattering into the detector and thermal grating signal.^19,33^ Importantly, for well averaged 2D-IR spectra these effects are limited to the diagonal.

Despite showing *ν*(OD) IR absorption spectra of qualitatively similar character, the ZSM-5 and ferrierite 2D-IR spectra of Fig. 2 are markedly different. The diagonal widths of both the positive and negative diagonal features of the ferrierite Brønsted stretch *ν*(ZOD) are larger in comparison to that of ZSM-5 (*ω*_1_ ∼ 2650 cm^−1^). Both samples' *ν*(ZOD) features have a narrow anti-diagonal width, consistent with these bands having a long vibrational lifetime^11^ (∼50 ps at this temperature). The broader diagonal band of the ferrierite ZOD stretch is particularly clear for the positive ESA feature and explains the bandshape of the ferrierite IR absorption spectrum as being due to several types of Brønsted site overlapping spectrally – one with a narrow (∼50 cm^−1^) range of absorptions and a second with a broader (∼100 cm^−1^) range of absorptions. An IR absorption spectrum cannot be used on its own to reveal that both bands have this type of (inhomogeneous) broadening. The 2D-IR spectrum therefore provides support for the IR absorption interpretation of Zholobenko *et al.*, who observed structured *ν*(ZOH/D) bandshapes in samples of ferrierite,^34^ with the *ν*(ZOH/D) stretch frequency variability thought to be due to weak interactions of the Brønsted hydroxyls with the more confined pore structure of ferrierite, despite there being fewer types of Al sites in ferrierite than in ZSM-5.

Although the ZSM-5 Brønsted *ν*(ZOD) feature is spectrally narrower than for ferrierite, ZSM-5 shows broad, distinctive 2D-IR bleach and ESA diagonal bands centred at pump frequency *ω*_1_ ∼ 2600 cm^−1^ that are completely absent from the ferrierite 2D-IR spectrum. In contrast to the *ν*(ZOD) band of ferrierite discussed above, these show a broader anti-diagonal width and increased bleach – ESA separation. These are the 2D-IR signatures of homogeneous broadening and anharmonicity. A most likely cause of this for a hydroxyl group is hydrogen bonding, which decreases the lifetime of the stretch modes^35^ and weakens the OH/D bond. As depicted in Fig. 1, for a given zeolite, the different types of hydrogen bonded hydroxyl-stretch moieties which could explain this band in the 2D-IR spectrum are Brønsted sites hydrogen bonded to framework oxygens (‘ZOD⋯Z’, Fig. 1(b)),^2^ Brønsted sites hydrogen bonded to water (
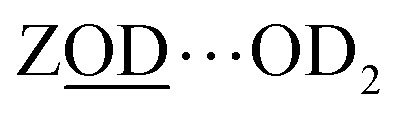
, Fig. 1(c)), Bronsted-bound water (
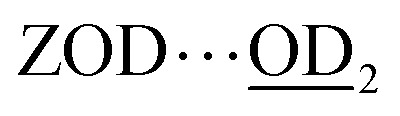
, 1(c)),^36^ hydrogen bonded bridging silanol defects (1(e)) and hydrogen bonded silanol nest vacancy defects (1(f)).

The consensus from IR analyses of H-form zeolites is that the ∼3500 cm^−1^*ν*(OH) equivalent to the ∼2600 cm^−1^*ν*(OD) band observed here is usually due to silanol nests.^7,22^ Although our assignment is therefore not unique, congestion in IR absorption spectra^10,23^ and ambiguity of the identity of the nest structures impede this conclusion. The IR, and even the NMR version of the band are sometimes assigned to water.^24,37^ Indeed, our IR/2D-IR observations show unusual characteristics reminiscent of liquid water – weakening/shifting with increasing temperature (ESI Section 8†) and ultrafast dynamical character (Section 6).

The process of silanol nest assignment by 2D-IR demonstrates some of its key features for hydroxyl analysis. Vibrational coupling cross peaks comprising bleach and ESA bands correlating the coupled modes are an important means in 2D-IR for making spectra-structure correlations not possible with conventional IR absorption spectroscopy. The distinct cross peaks of adsorbed water definitively rule it out as being responsible for the ∼2600 cm^−1^ band, as we shall see in Section 7. Non-hydrogen bonded SiOD groups (1(d)) show sharp features at ∼2760 cm^−1^ in the IR and 2D-IR spectra of Fig. 2. Our earlier 2D-IR study of pyrogenic silica showed that bridged-pair hydrogen bonded silanols and their non-hydrogen bonded partners display a very characteristic narrow 2D-IR cross peak accompanied by a distinct pattern of interstate coherence oscillations as a function of waiting time.^20^ The cross peaks for bridged silanols are not visible in Fig. 2, as they are dwarfed by the 2600 cm^−1^ band, which in Fig. 2(a) clearly does not display any narrow cross peak intensity correlated with high frequency non-hydrogen bonded silanol stretch *ν*(OD) modes. This implies a structure where all the silanol groups involved are hydrogen bonded to some extent – the characteristic of vacancy defects/nests. Though bridged silanol cross peaks are comparably weak in the ZSM-5 samples studied, they are observable in the 2D-IR spectra, especially after sodium exchange and at high temperatures, which disrupts the nests (see ESI Section 7†).

What of the Brønsted sites hydrogen bonded to the framework 
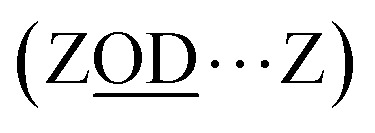
, and to water 
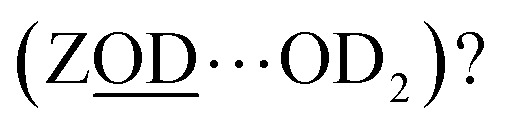
 Calculations by Windeck *et al.*^38^ show that it is unlikely for ZSM-5 frame-work hydrogen bonded Brønsted sites to absorb IR at (D-scaled) frequencies as high as 2600 cm^−1^ in ZSM-5. Their work, as well as earlier experimental work^2^ observes the ZOH⋯Z band's frequency to be centred at ∼3250 cm^−1^ and therefore lying in the range of ∼2450 cm^−1^ when deuterated. For 
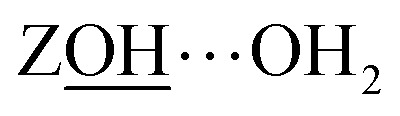
, at water loadings of *n* ∼ 1 per Brønsted site, a Brønsted site donating a hydrogen bond to water displays a remarkably broad and intense ‘doublet’ IR absorption spectrum.^39^ Hack *et al.* reveal a 2D-IR spectrum just as distinct in character.^18^ The spectrum of the deuterated version was unknown to us, so we conducted IR absorption measurements on ZSM-5 encapsulated in CaF_2_ (ESI Section 2†) revealing the equivalent *ν*(OD)-stretch band to peak at 2200 cm^−1^. Once frequency scaled, we observe the 
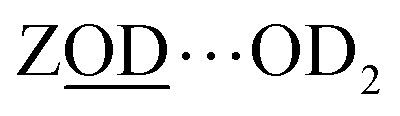
 Brønsted stretch band to be similar in shape and width to the OH version. The wings of this band are seen in the 2D-IR spectrum of *n* ∼ 1 loadings of D_2_O in ZSM-5 at the earliest waiting times (*e.g.*Fig. 4(a)). The band disappears on heating, as expected for a feature associated with water.

## 4. 2D-IR spectroscopy of silanol nests in ZSM-5 and silicalite

To further explore the assignment of the ∼2600 cm^−1^*ν*(OD) band in the 2D-IR spectrum of ZSM-5 as silanol nests we examine silicalite-1, which has same MFI-type framework as ZSM-5 but contains no aluminium within the framework (ESI Section 1†). Silicalite contains a high density of internally hydrogen bonded vacancy defect silanol nests,^21^ giving rise to a broad *ν*(OH) IR absorption feature at ∼3450 cm^−1^, which for *ν*(OD) corresponds to ∼2600 cm^−1^. Given the potentially different makeup of the defects in silicalite and ZSM-5, we explore their degree of similarity. This required careful control of temperature and humidity. Anhydrous conditions were achieved using CaF_2_ sandwiches of sample as described in the methods section. The *ν*(OH/D) bands of the nests, Brønsted sites and free silanol shift, broaden and weaken as a function of temperature (ESI Section 8†). At temperatures below ∼50 °C, nitrogen weakly hydrogen bonds to the ZSM-5 Brønsted acid sites and generates a band overlapping with the 2600 cm^−1^ nest band. The presence of water has the effect of broadening the nest band at lower temperatures and making ZSM-5 and silicalite bandshapes differ. It is well known that high temperature calcination may generate defects through steam induced dealumination of the zeolite framework.^1^ High temperatures (humid or dry) also accelerate nest loss.^40^ We observed silicalite to undergo irreversible loss of nest band on rapidly cycling the temperature from 40 to 520 °C and back. The ZSM-5 samples were already calcined to proton form at 600 °C. Upon further temperature cycling, irreversible loss of the ZSM-5 nest band occurred to a lesser degree over longer timescales. These effects are described in ESI Section 8.†

For IR absorption spectroscopy in transmission, it can be difficult to deduce shapes of broad bands when they overlap with positive apparent absorption caused by frequency dependent scattering. As well as localising the optical scatter on-diagonal, scatter in 2D-IR spectra is also in-principle subtractable through a measurement at a long negative waiting-time. In this respect, 2D-IR bandshapes reflect genuine absorption differences more reliably compared with conventional IR spectroscopy. Diffuse reflectance IR spectroscopy (DRIFTS) is very common for determining IR spectra of powdered materials, but with the potential for specular reflection and requirement of determining a separate background to the sample studied from a standard powder, DRIFTS does not always measure accurate absorbance and bandshapes. 2D-IR does not require separate measurement of a background sample or empty cell to calculate a spectrum – all data is collected on the sample.

**Fig. 3:**
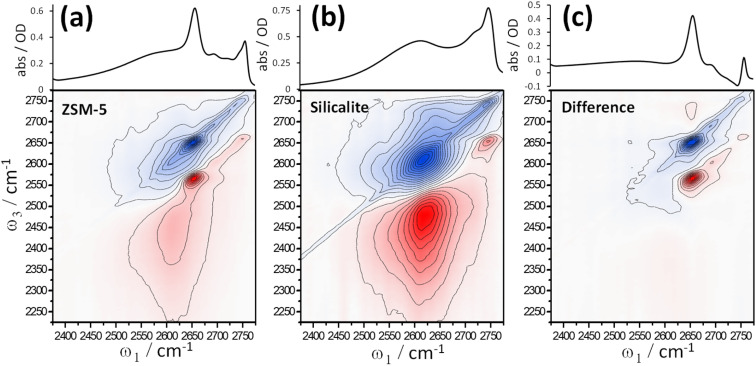
IR absorption and 2D-IR spectra of deuterated pellets of ZSM-5 (a) and silicalite (b) held at ∼210 °C between CaF_2_ plates in an atmosphere of dry N_2_. Crossed polarisation 〈XXYY〉 and a waiting time of *t*_2_ = 1 ps were used. Subtractions of the silicalite from the ZSM-5 2D-IR and IR spectra are shown in (c). The IR absorption spectra are baseline corrected using a ‘best-guess’ curve for the scatter contribution. A scale factor of ∼0.5 applied to the silicalite spectrum best subtracts the nest band in the ZSM-5 2D-IR and IR spectra.

Fig. 3 shows 2D-IR and scatter-baseline corrected IR absorption spectra of (a) deuterated ZSM-5 and (b) silicalite at ∼210 °C in an atmosphere of dry N_2_. The 2D-IR spectra are at a waiting time *t*_2_ = 1 ps, eliminating the diagonal spectral artefact caused by inter-laser pulse thermal grating effects.^19,33^ Other waiting times collected over a range of temperatures are shown in ESI Section 9.† In Fig. 3, the similarities between ZSM-5 and silicalite IR absorption and 2D-IR spectra are clear. This is further explored by subtracting scaled spectra of silicalite from spectra of ZSM-5 (Fig. 3(c)). For both IR absorption and 2D-IR spectra, a scale factor of ×0.5 applied to the silicalite spectrum removes the 2D-IR signal and ∼80% of the band's IR absorption. Given that the 2D-IR subtraction does not use baseline correction and has additional constraints of anharmonicity and 2D bandshape, Fig. 3(c) is a strong statement of the similarity of the two samples. The residuals are the Brønsted site and some of the higher frequency silanol defect bands stronger and therefore more abundant in the silicalite. At *ω*_1_ ∼ 2650 cm^−1^ and *ω*_3_ ∼ 2725 cm^−1^ the weak positive difference band in Fig. 3(c) is related to the Brønsted site. ESI Section 10† shows further examples of ZSM-5 – silicalite spectral differences.

In the *t*_2_ = 1 ps spectra of Fig. 3, silicalite shows a clear cross peak in the vicinity of *ω*_1_ ∼ 2625 cm^−1^ and *ω*_3_ ∼ 2710 cm^−1^ (Fig. 3(b)). It is not as evident at *t*_2_ < 0.25 ps and grows as a function of waiting time. Involving the nest band and a slightly weaker hydrogen bonded band, we observe this feature to be weak/absent in lower temperature 2D-IR spectra (ESI Sections 9 and 10†). The cross peak is also present in the ZSM-5 data, though harder to see in Fig. 3. ESI Section 10† shows data for *t*_2_ = 10 ps, where the cross peak is clearer in ZSM-5. The successful 2D-IR subtraction of the silicalite and ZSM-5 spectra in Fig. 3(c) and ESI Section 10† shows the cross peak is common to the two samples. The temperature dependence and ‘grow-in’ with waiting time points to dynamic structure effects such as nest chemical exchange or hydrogen bond weakening on vibrational relaxation. We will show in Section 6 using more direct forms of 2D-IR dynamics measurements that the nest band is indeed structurally dynamic on picosecond timescales. Next, we explore another feature of 2D-IR: its ability to make IR analysis quantitative.

## 5. Estimating silanol nest concentration relative to Brønsted site concentration

Being able to quantify the amount of nest hydroxyl groups observed by 2D-IR in zeolite samples would make IR analysis more powerful, and more complementary to NMR and XRF analysis. IR and 2D-IR spectral intensities only relate to chemical composition of a sample once calibrated for absorption coefficient, unlike XRF (ESI Section 1†), NMR^23^ or neutron techniques,^41^ where the relation of signals to concentration is direct. Under hydrogen bonding, the IR absorption coefficient of a band is difficult to experimentally deduce when the concentration of the species generating the band isn't known. Hydroxyl-stretch absorption coefficient and homogeneous IR bandwidth both increase with increased levels of hydrogen bonding. Though counteracting each other, this nevertheless leads to uncertain IR absorption strength and 2D-IR signal size.

Examples circumventing the problem of unknown IR absorption coefficient for hydroxyl analysis in ZSM-5 include (i) referencing the IR bands to solid state NMR spectra,^23^ (ii) titration with pyridine^42^ (for Brønsted sites) and (iii) use of empirical relationships between transition strength and wavenumber (for estimating levels of water coordination).^17^ 2D-IR measurements offer a direct, titration and model-free route to concentration determination.^26^ The approach is based on the fact that IR absorption *A* and 2D-IR diagonal bleach signals *S* are fundamentally related, and scale as the square and fourth power of the IR transition strength respectively. From these relationships, using the following simple formula, the concentrations *c* of sample components *a* and *b* can be determined without knowledge of the absorption coefficients or sample pathlength:^26^1
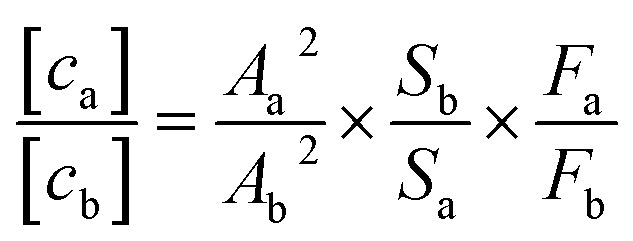


Measurement of the IR absorbance and 2D-IR signal size of diagonal bleach or excited state absorption bands from the same sample allows determination of the concentration ratio. The factors *F* are corrections to the 2D-IR signal accounting for effects such as overlap of diagonal bleach and ESA signal, pump laser attenuation through the sample, pump spectral intensity variations, waiting time dependent signal relaxation and inhomogeneous broadening.

Of the bands in the ZSM-5 spectrum, the Brønsted site ZOD stretch is the most suitable for calculating a relative amount of nest. Silanol nest quantification is a relatively challenging application of eqn (1). With no definitive structural model, nests comprise between 2 and 4 hydroxyl groups (Fig. 1). They comprise at least two overlapped IR bands and it is uncertain to what extent transition dipole (excitonic) coupling of the hydroxyl groups affect the form of the spectrum. Further complicating factors from the pelleted samples are the strong diagonal scatter and the thermal artefacts at early waiting times. Bearing these issues in mind, we nevertheless proceed with the idea that application of eqn (1) provides a reasonable measure of the ratio of main nest-band hydroxyl groups to Brønsted hydroxyl groups.

We applied eqn (1) [*c*_nestOD_/*c*_ZOD_] concentration ratio calculations to the Zeolyst ZSM-5 sample over several different temperatures and measurement sessions (ESI Section 15†). We observe the ratio to be consistently in the region of ∼1 ± 0.3 meaning that ZSM-5 IR and 2D-IR spectra such as in Fig. 2 are indicative of silanol nest hydroxyls that are as numerous as the Brønsted acid groups. The samples examined had been exposed to N_2_/D_2_O vapor at elevated temperatures for long periods of time, resulting in some nest loss and Brønsted site loss (ESI Section 8†). Using a transition dipole square ratio calculation,^26^ we calculate the transition dipole ratio *μ*_nestOD_/*μ*_ZOD_ to be ∼1.5. The Zeolyst ZSM-5 sample was observed by XRF to have a total Si : Al ratio of 24 and by NMR to have a framework Si : Al ratio of ∼32 (ESI Section 1†), meaning ∼3% of the Si lattice have Brønsted sites. It follows that nests might therefore be present at levels in the range of 1.5% (if diad) or 0.75% (if tetrad).

Recalling that the nest bands are totally absent from the 2D-IR spectrum of dehydrated ferrierite in Fig. 2(b), we also collected 2D-IR spectra from a different-source of ZSM-5 with similar Si : Al ratio to that described above (‘Mobil’ ZSM-5, ESI Section 7†). By 2D-IR, this ZSM-5 shows an almost complete absence of silanol nests. At high temperatures, the bridged silanol cross peak is weakly visible, but dwarfed in size by the ZOD acid site 2D-IR signal. Removal of the Brønsted site by Na^+^ exchange leaves a 2D-IR spectrum comprising diagonal and cross peak features from bridged pairs of silanol groups, but no nests. These comparisons confirm that the presence of silanol nests depends not only on the structure of a zeolite but also on its synthesis/treatment history.

## 6. The ZSM-5 silanol nest 2D-IR bands show distinctive ultrafast dynamics

**Fig. 4:**
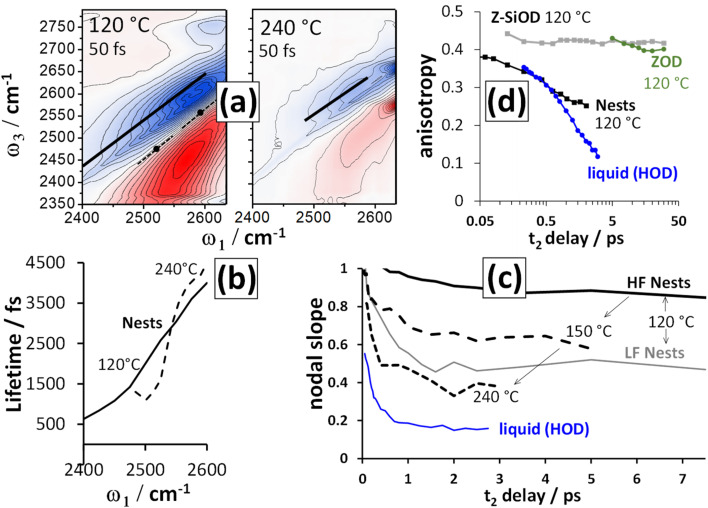
Lifetime, spectral diffusion and anisotropy decays of the ZSM-5 silanol nests. (a) 2D-IR spectra of ZSM-5 in flowing N_2_/D_2_O vapour were collected at 120 °C and 240 °C (〈XXYY〉 polarisation, *t*_2_ ∼ 50 fs). (b) The spectra in (a) are analysed as a function of waiting time *t*_2_ by single exponential fitting. (c) Spectral diffusion (nodal slope) around two different *ω*_1_ values at three temperatures. (d) anisotropy decays calculated from the positive ESA bands for the 120 °C measurement using both 〈XXXX〉 and 〈XXYY〉 data. In (a), the positions where lifetimes and nodal slopes were determined are indicated by solid and dotted lines. Shown for comparison in (c) and (d) are measurements of room temperature liquid HOD in H_2_O and (d) free silanol and the Brønsted site.

2D-IR spectroscopy is intrinsically a time resolved technique. As a function of waiting time (*t*_2_), the 2D-IR signal amplitude reports on a vibrational mode's lifetime. The changes in the slope of the 2D-IR diagonal bands reports on structural dynamics^13^ and the anisotropy^43^ calculated from polarisation dependent measurements reports on rotational motions and excitation hopping. In this section, we will show that the silanol nest diagonal 2D-IR bands of ZSM-5 and silicalite display distinctive behaviour for all three of these properties, as summarised in Fig. 4 for ZSM-5. The vibrational lifetime measurements point to there being two overlapping nest bands. 2D-IR slope and anisotropy measurements point to the nest bands being structurally dynamic in ways that other framework hydroxyl groups are not. These new insights into silanol nests indicate that further 2D-IR studies may be used to help in understanding what the structures are.

Fig. 4(a) shows 2D-IR spectra centred on the silanol nest band of ZSM-5 in flowing N_2_/D_2_O vapour at 120 and 240 °C and recorded at an early waiting time of 50 fs. The instrument response was ∼80 fs. Without cell windows in the beam focus, free-standing pellets show minimal disturbance from optical Kerr signal around *t*_2_ = 0 ps. The 120 °C spectrum shows a cross peak due to water discussed in the next section, though this does not affect the overall conclusions. The 120 °C spectrum also displays stronger bleach intensity than the 240 °C spectrum between *ω*_1_ ∼ 2400–2500 cm^−1^. This additional signal has a very short lifetime and is due to the Brønsted sites that donate a hydrogen bond to a single D_2_O molecule 
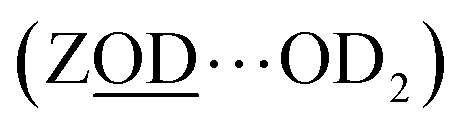
.

The frequency dependent vibrational lifetimes of the 2D-IR bleach signal at positions across the silanol nest band (black diagonal lines, Fig. 4(a)) were estimated by single exponential fitting. The lifetimes are shown as a function of pump frequency *ω*_1_ in Fig. 4(b). At both temperatures, the fitted lifetimes show a strong frequency dependence. The isotropic bleach signal (constructed from polarisation dependent spectra to eliminate rotational motions) and ESA show this same behaviour (ESI Section 11†). Even though the silanol nest IR absorption and 2D-IR bleach appear as one continuous band at these temperatures (Fig. 2(a)), the sloped character of the lifetime *vs. ω*_1_ plot in Fig. 4(b) implies two overlapping bands with different lifetimes, similar to earlier observations of vibrational relaxation of anions in ionic liquids.^44^ In the regions of spectral overlap, biexponential decay of the signal occurs (see ESI Section 11†). We estimate that the ∼2550 cm^−1^ band has a lifetime of ∼1.5–2 ps and the ∼2600 cm^−1^ band a lifetime of ∼5–6 ps. The lifetimes are not measurably sensitive to temperature between ∼100 and 300 °C. Cooling a dried sample below 100 °C to the absolute exclusion of water, the nest band of both ZSM-5 and silicalite does become structured, with features at ∼2600 and ∼2550 cm^−1^ indeed indicating two kinds of nests (ESI Sections 9 and 10†). Given the ambiguity in the composition of the nest band in the IR and 2D-IR spectra of Fig. 2 and 3, vibrational relaxation clearly provides important constraints for multiple-component-fits of more complex systems of overlapped nest bands, such as those reported recently by Cruchade *et al.* for ion exchanged, hydrothermally processed silicalite.^45^

A free silanol (SiOD) or Brønsted ZOD stretch has a temperature dependent lifetime of ∼50–100 ps, as observed in some of the earliest picosecond IR pump–probe measurements.^35^ When any R–OD/H group increases strength of participation in hydrogen bonding, or interacts with a greater number of neighbours through hydrogen bonding, the rate of vibrational relaxation increases.^35,46,47^ This is also seen in 2D-IR observations of hydroxyl molecular chains^48^ and anions in protic ionic liquids.^44^ The two types of silanol nest bands identified in Fig. 4 vary by a factor of ∼3 in lifetime, the lower frequency nest therefore possessing stronger/more hydrogen bonds. From the available data it is not yet possible to make an assignment from the possibilities in Fig. 1(f). For reference, the lifetime of a hydrogen bonded silanol group in a bridging silanol dimer/chain (Fig. 1(d)) is observed to be in the 5–7 ps range;^20^ similar but slightly higher than that of the 2600 cm^−1^ nest band. In the liquid state, lifetimes of ∼1.5 ps (21 °C) and ∼2 ps (70 °C) are observed for the OD stretch of HOD in H_2_O,^49^ comparable to the lifetime of the 2550 cm^−1^ nest band.

The narrow anti-diagonal width of the 2D-IR spectra of Fig. 4(a) at the early waiting time of 50 fs shows that the silanol nest bleach and ESA bands are initially inhomogeneously broadened. This means that for each nest band there are a distribution of structures possessing unique absorption frequencies. These structures are dynamic, as evidenced by a rapid evolution to a broader anti-diagonal width. This evolution of the diagonal 2D-IR bandshape is called spectral diffusion and is the result of structural motions causing fluctuations in the absorption frequency during the waiting time *t*_2_ – a common occurrence for liquid-phase hydrogen bonded species. For zeolite pellets, the most common measure of inhomogeneity, the bleach band centre-line-slope, is contaminated with pump scatter and thermal signal. The nodal slope^13^ (line of zero signal) between the interfering negative bleach and positive ESA bands is not affected and therefore the more practical measure of inhomogeneity here. A nodal slope value of 1 on the timescale of the measurement (full correlation between pump and probe frequencies) corresponds to the distribution of nest structures and their absorption frequencies being distinct across the band (inhomogeneous broadening). A slope value of zero is (i) the result of structural motion occurring such that the absorption frequencies randomise, or (ii) the result of the lifetime of the vibrations being short (homogeneous broadening).

The ZSM-5 2D-IR nodal slope decay plots in Fig. 4(c) were determined from the 〈XXYY〉 perpendicular polarised 2D-IR spectra as a function of waiting time around two pump frequency (*ω*_1_) ranges and at three temperatures. Room temperature liquid HOD/H_2_O hydrogen bond dynamics are shown for comparison. The nodal slope decays are pump-frequency and temperature dependent. These trends are shown in Fig. 4(c). At 120 °C, measuring the nodal slope ±40 cm^−1^ around the lower 2520 cm^−1^ pump frequency (‘LF’), we isolate a slight, fast, ∼100 fs slope decay discernible from the instrument response followed by ∼1 ps decay to an offset of 0.5. At ±30 cm^−1^ around the higher value of 2580 cm^−1^ (‘HF’), the slope decays by only 0.1 over 3 ps, but, in this same frequency range at 240 °C, the nodal slope decays in ∼1 ps to an offset of ∼0.4. These slope decays are a signature of structural dynamics of the two nest bands identified from Fig. 4(b). To further confirm this, 2D-IR spectroscopy offers an additional type of dynamics measurement: anisotropy, which we discuss next.

Anisotropy is a measure of molecular alignment and in time-resolved vibrational spectroscopy can access a functional group's rotational dynamics. For time resolved IR techniques such as 2D-IR, an anisotropy measurement is made by exciting a vibrational band with the pump laser, then measuring transient spectra of the excited molecules in both parallel and perpendicular probe polarisations. The anisotropy is calculated as (*S*_para_ − *S*_perp_)/(*S*_para_ + 2*S*_perp_).^43^ Exciting an isotropic sample such as a liquid or disordered solid with polarised light, for diagonal 2D-IR bands, an anisotropy of 0.4 represents the maximum alignment of vibrationally excited molecules achievable. Loss of rotational order of the excited vibrational modes occurs over time through random rotational motions. An anisotropy of zero corresponds to loss of all excitation alignment induced by the pump laser.

For determination of anisotropy in 2D-IR spectroscopy, the diagonal bands of interest must be strong relative to other overlapping modes or cross peaks and must not contain any scattered pump light. In the bright probe experimental arrangement used,^19^ a well averaged pair of parallel (〈XXXX〉) and perpendicular (〈XXYY〉) 2D-IR measurements were found to localise the scatter to a region smaller than the total anti-diagonal width of the ZSM-5 nest bleach, allowing anisotropies to be reliably calculated outside this region. Anisotropy values for the 1 → 2 diagonal ESA band and all other off-diagonal regions were also readily observable. That dynamic 2D-IR anisotropies can be measured from such optically scattering pelleted samples is a significant finding. Examples of the 2D-IR spectra measured in both polarisations are shown in ESI Sections 5 and 12.†

The anisotropy of the 120 °C ZSM-5 2600 cm^−1^ silanol nest band measured near to the centre of its 2D-IR ESA feature is shown in Fig. 4(d). It is observed to decay exponentially with *t*_2_ to an offset of 0.25 with time constant of ∼900 fs. This is a characteristic of constrained rotational motion. For reference, the anisotropy decay of 25 °C liquid HOD in H_2_O at bleach-band centre is shown, decaying to an offset of 0.1 with a 2.2 ps time constant. The dynamical motion of the silanol nest modes suggested by both nodal slope and anisotropy decays stands in contrast to the behaviour of other zeolite framework vibrational modes. In Fig. 7(d), the long-lived free silanol SiOD ESA feature of ZSM-5 at *ω*_1_ ∼ 2760 cm^−1^ clearly does not undergo any rotational motion over the lifetime of the excited vibrations, maintaining an anisotropy of ∼0.4 for >30 ps. For the other framework modes above *ω*_1_ ∼ 2600 cm^−1^, once overlapping bound-water signals have decayed by around 12 ps, the ESA signals of the remaining Brønsted OD stretch (ZOD), extra-framework aluminium (AlOD) and other weakly hydrogen bonded silanol absorption in the ZSM-5 2D-IR spectrum also show time invariant anisotropy values of ∼0.4. This is because the hydroxyl oxygen's near-tetrahedral sp^3^ bond configuration forces the OH groups into fixed orientations relative to the framework.

In assessing anisotropy decay, excitation hopping mechanisms similar to Förster resonance energy transfer also occur in concentrated extended hydroxyl systems. The orientation of one excited donor hydroxyl group might be different to that of a neighbouring acceptor, so the anisotropy will decay under excitation hopping.^50^ A means of switching off this effect is to perform H/D isotope dilution. Though we show that the isotope diluted nest 2D-IR spectrum of ZSM-5 is similar in appearance to that at 100% deuteration (ESI Section 3†), we did not yet study the isotope-diluted nest anisotropy decay. To further confirm that the anisotropy decay is mostly structural dynamics, we instead turn to silicalite. As well as being easier to keep dehydrated and free of bound N_2_ over a wider range of temperatures, the higher density of silanol defects gives stronger 2D-IR signals without contamination from the strong Brønsted OD stretch band. Importantly, the silicalite isotropic and anisotropic signal decays are observed to be practically identical to ZSM-5 (ESI Section 13†).

**Fig. 5:**
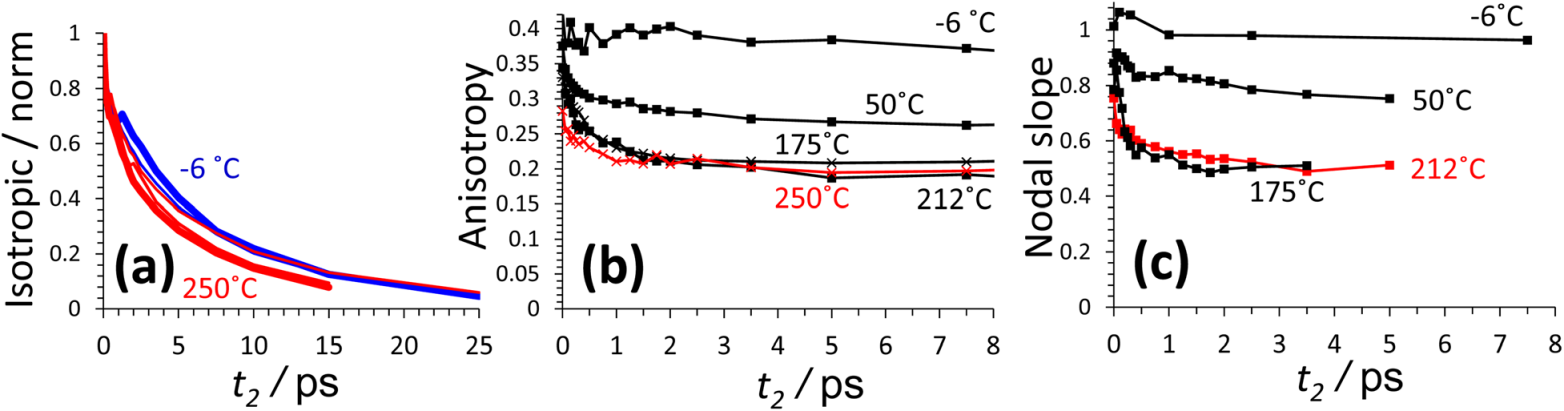
Temperature dependence of the silicalite 2D-IR normalised isotropic signal (a), anisotropy (b) and nodal slope (c) for the 2600 cm^−1^ silanol nest band of silicalite. The overlapped isotropic signals in (a) comprise five temperatures: −6, 50, (both in blue) and 175, 212 and 250 °C (in red). The two extrema are plotted with thicker lines. Anisotropy was obtained by averaging five 2D-IR data points in a 16 × 16 cm^−1^ square around the peak of the excited state absorption (ESA) 1 → 2 signal. The anisotropy decays have been plotted to the same 8 ps final *t*_2_ as the nodal slope value (latest reliable waiting time), but extend out to 30 ps (ESI Section 13†).

Fig. 5(a)–(c) shows silicalite's normalised ESA isotropic signal (reporting on vibrational lifetime), anisotropy and nodal slope decays around the peak of the *ω*_1_ ∼ 2600 cm^−1^ silanol nest band as a function of temperature. The temperature range of the five isotropic signal decay measurements spans −6 to 250 °C and though the highest temperature decays slightly faster than the lowest, the temperature dependence of the decay rate is relatively weak (∼10%). In contrast, for liquid water, owing to weakening of hydrogen bonds, the relaxation rate for the OD stretch of HOD decreases with temperature by ∼30% from 4 to 70 °C.^49^

Whereas the silicalite isotropic signal decay hardly changes with temperature, the anisotropy decay varies significantly between −6 °C and 175 °C (Fig. 5(b)), but changes less from 175 to 250 °C. The nodal slope decays show the exact same trends (Fig. 5(c)). It is significant that at low temperatures the structural dynamics ceases but vibrational relaxation is unaltered. Vibrational relaxation rate and vibration-hopping rate are both very sensitive to hydrogen bonding strength.^51^ If the anisotropy decay were only due to excitation hopping, we would expect a corresponding variation of the isotropic signal decay with temperature, as well as no nodal slope decay. There could be some hopping contribution to the anisotropy decay, however Fig. 5 shows that the nodal slope and anisotropy are sensing fast structural motions of the nests. The dynamic cross peak discussed in relation to Fig. 3 is further evidence of mobility. These dynamics are possible because each nest hydroxyl (deuteroxyl) group is attached at the point of the connectivity defect to a different silicon tetrahedron in the framework. It is plausible that thermal motions of the framework then cause the nest hydroxyl structures to fluctuate. Indeed, zeolite Y classical MD simulations report a larger amount of motion for the nest SiOH groups than the Brønsted ZOH groups.^52^ We also analysed the anisotropy decay of the lower frequency nest band at around *ω*_1_ = 2500 cm^−1^ and observe faster decays to a lower offset and similar temperature dependence (ESI Section 14†). This could be the stronger hydrogen bonding leading to more rapid constrained motion, as has been observed by IR anisotropy measurements on nano-confined water in metal–organic-frameworks^53^ and Nafion.^51^

## 7. 2D-IR spectra of water in zeolites

In Section 3, we discussed how bound water in zeolite 2D-IR spectra can be identified through its cross peaks, using this fact to discount bound water as the origin of the 2600 cm^−1^ band in Fig. 2(a). As water is of fundamental importance in many areas of zeolite research, in this final section we show several different cases of hydration water 2D-IR spectra, the new data hinting at some of the opportunities that 2D-IR can provide for studying hydration water in zeolites and other solids.

**Fig. 6:**
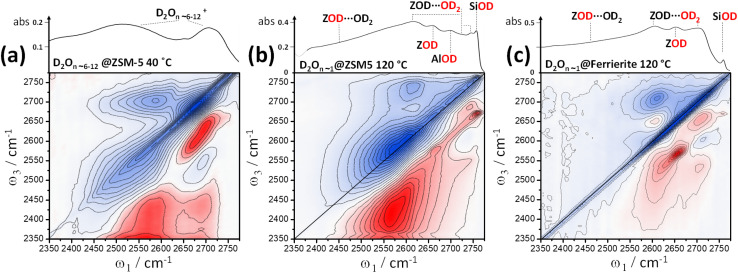
Water in zeolites. The strong anharmonic coupling of each water molecule's two stretch modes results in intense 2D-IR cross peaks. (a) Water clusters dominate the 2D-IR and IR absorption spectra of zeolites at 40 °C. (b) At an elevated temperature (120 °C), the clusters are lost, and ZSM-5 water cross peaks take on a distinct form for single water molecules (*n* ∼ 1 per acid site), with the other framework hydroxyls also visible. (c) Ferrierite under similar conditions to (b). The 2D-IR spectra were recorded at a waiting time *t*_2_ ∼ 400 fs with crossed pump–probe polarisation 〈XXYY〉. In (a), the ZSM-5 sample is 5× thinner than in (b) and (c), held tightly between CaF_2_ windows and after drying and H–D exchange, cooled to ∼40 °C and equilibrated in a stream of N_2_/D_2_O vapour.

Unlike in the liquid state, adsorbed water in zeolites gives richly structured IR spectra. This manifests also in the 2D-IR spectra. Fig. 6 shows three examples. The key characteristics are distinct cross-peaks in the off-diagonal parts of the 2D-IR spectrum arising from (i) the anharmonic (intramolecular) coupling of each water molecule's two stretch modes for isolated molecules, and from (ii) intermolecular coupling effects for clusters and chains. These effects are obscured in liquid water,^54^ but in zeolites and other hydrated solids, we observe that they provide a powerful means of distinguishing water of hydration from framework hydroxyl bands.

At <80 °C temperatures, water forms branched, protonated clusters in ZSM-5 comprising a heterogeneous distribution of weakly hydrogen bonded water (higher frequency region), and strongly more hydrogen bonded water (lower frequency region). Hack *et al.*^17^ observed the 2D-IR cross peak reporting on strong-weak water coupling to extend across these two regions to the lowest frequency proton continuum band. Our 2D-IR measurement of protonated clustered D_2_O in ZSM-5 is shown in Fig. 6(a). From the IR absorption spectrum and loading behaviour, we expect the water cluster sizes in this sample to be *n* ∼ 6–12 ‘per’ Brønsted site (ESI Section 3†). Water molecules complexing the excess proton absorb at frequencies red-shifted outside of the plotted spectral range, so Fig. 6(a) is showing the non-proton solvating water. The intense cross peaks arise from a combination of strong intra-molecular and weaker inter-molecular vibrational coupling across the clusters. Two overlapped higher frequency ‘weak’ hydrogen bonded diagonal peaks display a narrow anti-diagonal bandwidth. Though the inhomogeneous broadening is greater in the lower frequency ‘strong’ hydrogen bonded band, its anti-diagonal width is also greater from increased lifetime broadening and perhaps spectral diffusion. Our observations of protonated water clusters in the nano-confining pores of Nafion show cross peaks that are much less resolved: an indication of greater disorder and fewer unterminated hydrogen bonds for water in Nafion.^51^ In ESI Section 3,† we show further isotope diluted ZSM-5 HOD/H_2_O 2D-IR spectra and waiting time dependences of the different water loading 2D-IR spectra.

Lower water loadings are shown in Fig. 6(b) and (c) for ZSM-5 and ferrierite in a regime of *n* ∼ 1 molecule per Brønsted site. Similar to IR spectroscopy,^55^ loading is determined by grow-in of the free Brønsted site ZOD (*n* ∼ 0) and 
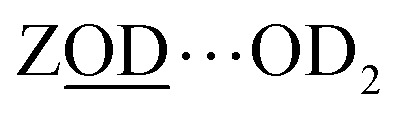
 (*n* ∼ 1) complex bands in the 2D-IR spectra. Though we expect these to indicate the *n* ∼ 1 regime, loading was not controlled to the extent of ruling out a small proportion of Brønsted sites having *n* ∼ 2. In ESI Section 4,† the loss of the cross peaks in ZSM-5 and correlation to the appearance of the free Brønsted site as a function of increasing temperature is quantitatively demonstrated by 2D-IR and IR pump–probe temperature-ramp spectroscopy.

In Fig. 6(b) and (c), the strong 
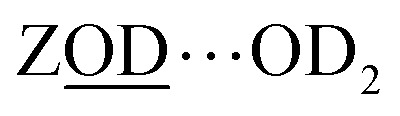
 band (Fig. 4(a)) has decayed at the *t*_2_ value of 400 fs displayed, giving a clearer view of the remaining water cross peaks and framework modes. Single water molecules likely accept a hydrogen bond from the Brønsted site and form single hydrogen bonds to a framework oxide ion, with one hydrogen bonded and one non-hydrogen bonded stretch mode therefore present. These are known to absorb at around 2620 and 2730 cm^−1^ (H–D scaled^56^) and correlate well with the strongest cross peak bleach features in the 2D-IR spectra 6(b) and (c). From the peak–peak separation of the cross peak bleach and ESA, we see that the two stretch mode ‘single’ water molecule anharmonic coupling is ∼60–70 cm^−1^. This value increases with hydrogen bond strength, so the apparent value of ∼150 cm^−1^ for the hydrogen bonded ZSM-5 clusters (better visible below-diagonal, Fig. 6(a)), is plausible. An example of even stronger intramolecular coupling is ice, ∼200 cm^−1^.^57^

Whereas ferrierite has a channel dimensionality of two, ZSM-5 is three dimensional and has a greater diversity of aluminium substitutions. The ZSM-5 pore intersections key to its catalytic properties have increased space for water than the more confining channels. Owing to these differences, the ferrierite 2D-IR spectrum in Fig. 6 has more uniform cross peak shapes. The more complex, extended cross peaks in ZSM-5 indicate that the water has a more heterogeneous character in comparison to the more limiting/confining ferrierite.

**Fig. 7:**
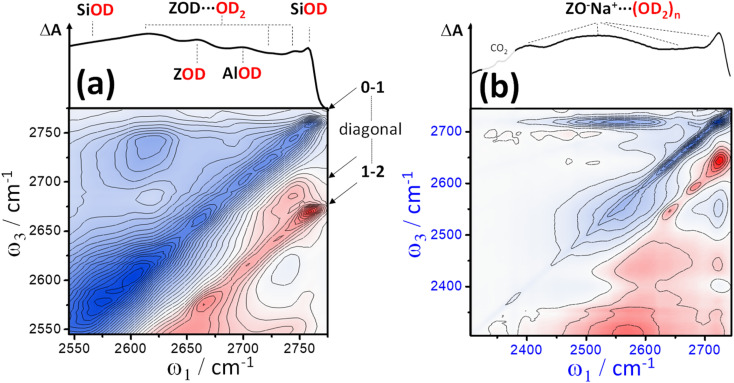
(a) A zoom-in of the Fig. 6(b) ZSM-5 2D-IR spectrum reveals multiple water *ν*(OD) stretch bands contributing to the complex cross peak shapes. Some of these modes are observable along the diagonal bleach line (0 → 1). Owing to their different relative anharmonic shifts, for the positive diagonal ESA (1 → 2) signal, several water modes separate clearly from the narrow inhomogeneously broadened line of SiOD, AlOD and ZOD bands. (b) IR absorption and 2D-IR spectrum of D_2_O in sodium zeolite X at ∼220 °C (*t*_2_ ∼ 400 fs, 〈XXYY〉 polarisation).

Fig. 7 shows two further examples of zeolite-bound water 2D-IR spectra. 7(a) is a zoom-in of the *n* ∼ 1 water loading ZSM-5 spectrum of Fig. 6(b). Cross-peak bleaching spreads across the upper part of the spectrum, hinting at multiple water binding configurations. There is a remarkable level of detail in the contours of the diagonal 1 → 2 ESA features (indicated by arrows). Around *ω*_1_ ∼ 2730–2775 cm^−1^, three types of water stretch bands are visible (two very narrow in 2D-width and one broad). The broader component has the character of the corresponding cross peak for water in the smaller-pore ferrierite and could therefore be a signature of channel water in ZSM-5. In the IR absorption spectrum these overlap with the ZSM-5 framework hydroxyl groups. In the 2D-IR spectrum the water bands are clearly separated because their diagonal 1 → 2 anharmonicity is smaller than the framework groups, which in Fig. 7(a) comprise a strikingly uniform diagonal ESA line spanning the highest frequency SiOD group at 2760 cm^−1^ and the Brønsted site at 2650 cm^−1^. The property of identical anharmonicities for non-hydrogen bonded zeolite hydroxyl groups (*e.g.* ZOH *vs.* SiOH) so clearly displayed in the 2D-IR spectrum was described in anharmonic frequency calculations of Merkel.^58^ Identification of at least three unique water modes suggests that further study might support distinction between different water binding configurations^59^ and binding at different T-sites.

The intramolecular coupling cross peaks of single-bound water molecule 2D-IR spectra are simpler to understand than those of water clusters, where intermolecular coupling also occurs. The 2D-IR spectrum in Fig. 7(b) for the high aluminium content non-acidic Na^+^ zeolite X (Si : Al ratio of ∼1.2) illustrates this. Zeolite X contains a dense network of Na^+^ counter-ions throughout its supercages and sodalite cages and no framework hydroxyl species at all. Under hydration, extended chains of water molecules form,^60^ stable up to temperatures of ∼250 °C. Fig. 7(b) is a 2D-IR spectrum of strongly bound water chains in Na^+^ zeolite X at 220 °C. In comparison to the 2D-IR spectrum of ZSM-5 water clusters (Fig. 6(a)), the water-chain spectrum of zeolite X is more structured. For the ZSM-5 sample, NMR suggests a framework aluminium content of ∼32 (ESI Section 1†), corresponding to slightly less than 1 acid site per channel intersection. Only at temperatures <∼100 °C can small water clusters (*n* ∼ 4–7 (ref. 39)) form directly around these sites in the ZSM-5. The multiple distinct IR bands observed from the zeolite X water chains blur or disappear for the more disordered protonated water clusters of ZSM-5. Despite zeolite X water showing the same clear signatures of intermolecular coupling, in contrast to the ZSM-5 water clusters, the relaxation time of the lower frequency band (*ω*_1_ ∼ 2520 cm^−1^) zeolite X water diagonal signal of Fig. 7(b) is ∼5 ps, compared with ∼1.2 ps for the ZSM-5 water clusters (Fig. 6(a)). This is likely a reflection of ZSM-5 water's coupling to the stronger hydrogen bonded water and lower frequency proton complex modes^17^ not present in zeolite X water.

**Fig. 8:**
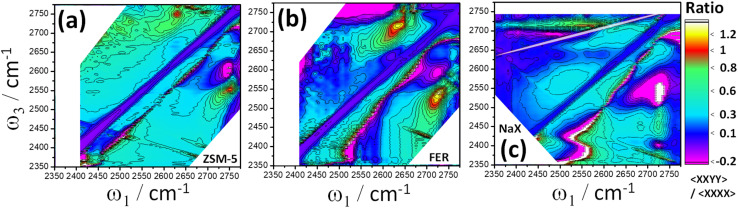
Polarisation ratio 2D-IR spectra of water (D_2_O) in ZSM-5 (120 °C, (a)), ferrierite (120 °C, (b)) and Na^+^ zeolite X (∼220 °C, (c)). All samples were held in flowing D_2_O/N_2_ vapor and recorded with *t*_2_ = 100 fs. Baseline corrections were applied to the below-diagonal side to remove artefacts (ESI Section 6†). The extra diagonal line (white, *ω*_3_ > 2675 cm^−1^) in (c) is a pump scatter and pulse-shaper artefact.

We demonstrated in Section 6 that measurement in parallel 〈XXXX〉 polarisation from scattering pelleted samples is feasible. This enables calculation of polarisation ratio 2D plots (or similarly, anisotropy plots). Cross peaks from vibrational modes with ∼90° relative transition moment angles (such water) theoretically give a maximal 〈XXYY〉/〈XXXX〉 polarisation ratio strength of 1.17. The weaker cross peaks often overlap with the stronger diagonal features in a 2D-IR spectrum, which, involving parallel transition moments, have a polarisation ratio of 0.33. Polarisation ratio 2D plots therefore serve to enhance the contrast of water cross peaks against the diagonal features. The three examples shown in Fig. 8 are the *n* ∼ 1 water cross peaks of Fig. 6 (ZSM-5 (a) and ferrierite (b)), and water chains of Fig. 7 (zeolite X (c)). ESI Sections 5 and 6† explain in greater detail theory and modelling of 2D polarisation ratio plots, show the raw parallel and perpendicular 2D-IR spectra and show further examples of water in partially sodium-exchanged ZSM-5.

The Fig. 8 ZSM-5 and ferrierite polarisation ratio 2D spectra have ratios of ∼0.8 in the cross peak regions, with ferrierite showing a maximum of 1.2. Owing to reducing the impact of the diagonal 1 → 2 ESA band, the below-diagonal cross peaks are clearer in the contours of the polarisation ratio spectra than the 〈XXYY〉 spectrum. The diagonal slope is a signature of the two stretch mode frequencies being correlated and inhomogeneously broadened, highlighting the structural heterogeneity of the bound water. Looking at the spectrum along either the *ω*_1_ or *ω*_3_ axes at ∼2750 cm^−1^, very narrow non-hydrogen bonded water cross peak features are reproducibly observed above and below the diagonal in both ZSM-5 and ferrierite polarisation ratio spectra. These show coherence oscillations as a function of waiting time confirming the features as genuine cross peaks, as described previously for pyrogenic silica.^20^ Whether these are non-hydrogen bonded water molecules oxide-bound to ZSM-5 or weakly-coupled silanol groups are interesting possibilities for further investigation.

In the zeolite X 〈XXYY〉 and polarisation ratio spectra (Fig. 7(b) and 8(c)), there is a striking asymmetry in the cross peaks above and below diagonal. In addition to the greater interference of the broad diagonal ESA band with the cross peaks below-diagonal, this is also due to substantial differences in cross peak ESA and bleach interference that occurs under asymmetric cross peaks and weak coupling.^20^ Similar to ZSM-5 water clusters, above diagonal, the zeolite X 2D-IR spectrum prominently displays the intense (narrower) bleach cross peak between the highest frequency non-hydrogen bonded band and the broader lower frequency hydrogen bonded band. In the 〈XXYY〉 spectrum, the expected ESA component(s) of the strongly coupled water modes for this cross peak are hard to make-out. The hint of cross-peak ESA ∼25 cm^−1^ red-shifted along the probe axis from the bleach cross peak is made very clear in the polarisation ratio spectrum. This pattern is similar in character to the 2D-IR spectrum of transition dipole-coupled bridging silanol groups,^20^ an indication that intermolecular as well as intramolecular coupling of the water molecules is playing a role in determining the form of the cross peaks. Below diagonal, two cross peak bleaches and their corresponding ESA bands are clear at *ω*_3_ ∼ 2550 and 2400 cm^−1^ with apparent anharmonic separations of ∼100 cm^−1^. However, for water intermolecular coupling in the 2D-IR spectrum of ice polymorphs,^57^ the vibrational energy states deviate significantly from the type of simple coupled 2-level system explaining the single-water 2D-IR spectra of ZSM-5 and ferrierite. The first excited state is thought to be delocalised by intermolecular coupling and the second excited state to be localised by the intramolecular coupling. To proceed with understanding zeolite X water chain 2D-IR spectra, an equivalent energy state model is required from the published structures.^60^ By varying the framework, temperature and humidity, zeolite studies allow for considerable control of the water chain sizes. Compared with ice, developing an understanding of water chain 2D-IR spectra in zeolites appears feasible.

The Zeolite X polarisation ratio of Fig. 8(c) reveals a weak ESA cross peak parallel to the pump axis cutting across the diagonal bleach of the broad hydrogen bonded water band (*ω*_3_ ∼ 2640 cm^−1^) and correlating directly with the strongest diagonal ESA feature. This feature may provide a clue to the missing strong-coupling ESA cross peak above diagonal. It is also reminiscent of a feature observed in transition dipole-coupled bridging silanol groups of pyrogenic silica^20^ thought to be a fifth-order cross peak, serving as a reminder to confirm such effects in any further analysis.

2D-IR spectra and polarisation ratio spectra clearly have potential for determining different hydration states of zeolites and other solids. With the ability to measure parallel 〈XXXX〉 spectra under strong scatter, an alternative laser polarisation scheme for suppressing the diagonal features ‘*in situ*’ is also made possible.^61^ The values of polarisation ratios and anisotropies of cross-peaks can be used for quantifying the angles between the water molecule's coupled transition dipoles.^17,54^ Might this combine with theory to better deduce the water binding structures? We explore this in ESI Section 5† and conclude that because of spectral congestion and backgrounds, current measurement of these angles are limited at present to ∼±15° accuracy. In ESI Section 6,† we use simulations to explore the effects of these backgrounds and spectral congestion on 2D-IR polarisation ratio values, demonstrating the striking artefacts they cause in 2D polarisation ratio plots.

## 8. Conclusions

We have shown that 2D-IR measurement of vibrational spectra of zeolites rewards IR investigations with structural and dynamical information not accessible to conventional IR absorption or NMR spectroscopy. In examining just some of the different kinds of hydroxyl groups present in ZSM-5, ferrierite, silicalite and zeolite X, we see that 2D-IR spectra show the acid sites, the silanols and the bound water of hydration with a higher degree of separation than conventional IR absorption spectroscopy. We see from 2D-IR Brønsted site bandshapes that owing to increased pore confinement, the inhomogeneous distributions of ferrierite acid site hydroxyl frequencies are wider. The sensitivity of 2D-IR to bandshape origins and anharmonicity is illustrated by the almost perfect subtraction of the silicalite silanol nest 2D-IR spectrum from the ZSM-5 2D-IR spectrum, supporting a conclusion that the nest bands in these two samples are exactly the same. Owing to the more complex 2D bandshapes, the confidence in such subtractions are greater than for IR spectra, which are more directly affected by the backgrounds caused by scattering. The observation of identical 2D-IR dynamical behaviour (vibrational lifetime, anisotropy and spectral diffusion) of the silicalite and ZSM-5 samples is equally strong evidence. The distinction between nest bands and bridged silanols through the characteristic cross peaks that the latter show is another powerful feature of 2D-IR not observable in a conventional IR absorption measurement.

An important benefit of 2D-IR is the ability to perform concentration ratio quantifications. In the particular commercial ZSM-5 sample studied we find that the nest hydroxyls are present in similar proportions to the Brønsted hydroxyls in the Zeolyst ZSM-5 studied.

That it is possible in the presence of vast amounts of scattered pump laser light to observe nodal slope spectral diffusion, polarisation ratio 2D-IR spectra and anisotropies for observing rotational dynamics was unexpected. This opens up these powerful dynamical measurements to a range of applications in solid state chemistry and catalysis. From the available 2D-IR data, quite unlike other framework hydroxyl groups, the nest hydroxyls appear to be structurally dynamic on a timescale of picoseconds. Demonstration of picosecond timescale anisotropy and spectral diffusion measurements of the silanol nest band are a powerful example of 2D-IR accessing dynamical information from zeolites not readily deducible by other experimental methods. The anisotropy decay and spectral diffusion^43^ extracted from 2D-IR spectra are simpler to relate to molecular dynamics simulation than a vibrational spectrum. With the growing use of *ab initio*/machine learning molecular dynamics simulations for predicting the catalytic behaviour of zeolites,^62^ 2D-IR can therefore provide valuable data for training and benchmarking simulation models.

In the presence of water, the IR absorption spectrum of zeolites becomes very crowded. We find that the difference in bond anharmonicities of water and framework modes separates them out in 2D-IR and that inter and intra-molecular couplings of the water molecules generate richly structured cross peaks which we hope in future can be put-to-use in deciphering how and where water is binding in these materials.

We have presented 2D-IR spectra of deuterated zeolites with a breadth and depth which we hope will stimulate further applications of the technique. Much of the data discussed are presented for the first time following years of investigation made possible by technical developments enabling 2D-IR studies of pelleted solids.^19^ The comprehensive ESI section† supplied aims to show the reader that the work presented here is reinforced by thousands of 2D-IR and IR measurements. It also shows that we have developed the 2D-IR technique to the point that measurements on zeolites and similar materials can now be routinely performed at the national facility of the UK's Rutherford Appleton Laboratory,^63^ where the data were collected. Here, 2D-IR spectroscopy is about to undergo step change improvements in spectral bandwidth, signal-to-noise and set-up time through new instruments incorporating Yb:KGW laser technology^64^ and designed with *in operando* study of heterogeneous catalysts such as zeolites in-mind.

## Data availability

Datasets supporting this paper are available from eData at the STFC Research Data repository https://edata.stfc.ac.uk/handle/edata/970.

## Author contributions

Paul Donaldson (investigation, writing – original draft, writing – review and editing, formal analysis, visualization, data curation, methodology, project administration, supervision, funding acquisition, conceptualisation). Alexander Hawkins (investigation, data curation), Russell Howe (writing – review and editing, funding acquisition, conceptualisation).

## Conflicts of interest

There are no conflicts to declare.

## Supplementary Material

SC-OLF-D4SC08093A-s001
