# Supplementary material for: Distinctive signatures and ultrafast dynamics of Brønsted sites, silanol nests and adsorbed water in zeolites revealed by 2D-IR spectroscopy
Source: Chem Sci. 2025 Mar 25;16(16):6688–704. doi: 10.1039/d4sc08093a (PMC11935784; doi:10.1039/d4sc08093a)
Supplement: SC-016-D4SC08093A-s001 [file SC-016-D4SC08093A-s001.pdf]

Supplementary Information (SI) for Chemical Science.
This journal is © The Royal Society of Chemistry 2025

Distinctive signatures and ultrafast dynamics of Bronsted sites, silanol nests and adsorbed water in zeolites revealed by 2D-IR spectroscopy

Supplementary information

Paul M. Donaldson,[1*] Alexander P. Hawkins,[1] Russell F. Howe,[2]

[1]Central Laser Facility, Research Complex at Harwell, STFC Rutherford Appleton Laboratory, Harwell Science and Innovation Campus, Didcot, OX11 0QX, UK

[2]Department of Chemistry, University of Aberdeen, Aberdeen AB24 3UE, United Kingdom

*paul.donaldson@stfc.ac.uk

Table of contents

	Section	Page
1	X-Ray Fluorescence and NMR analysis of the zeolites and silicalite	2
2	Observing the OD stretch of the deuterated ZSM-5 Bronsted sites hydrogen bonded to D_2O	5
3	2D-IR of water in ZSM-5: sample conditions, HOD/H_2O and D_2O 2D-IR spectra and waiting time dependence	7
4	The 2D-IR cross peaks from ZSM-5 bound water disappear as a function of temperature	11
5	Exploring zeolite water cross peaks using 2D-IR polarisation ratios	12
6	Factors to consider when interpreting 2D-IR polarisation ratio spectra	15
7	2D-IR signatures of bridged silanols in ZSM-5 samples	19
8	Temperature dependence of the silicalite and ZSM-5 nest band	20
9	ZSM-5 and silicalite 2D-IR spectra as a function of temperature and waiting time	24
10	Further difference spectra of ZSM-5 and silicalite	26
11	The decays of 2D-IR amplitude (vibrational lifetimes) reveal two silanol nest features	28
12	Parallel and perpendicular polarisation ZSM-5 2D-IR measurements used to calculate the anisotropy values of Figure 4(d) (main text)	29
13	Silicalite vs ZSM-5, anisotropy decays and lifetimes	29
14	The lower frequency silanol nest band has a faster anisotropy decay than the ~2600 cm-1 silanol nest band	31
15	Silanol nest / Bronsted site concentration ratio calculations	31
16	Bibliography	35

1. X-Ray Fluorescence and NMR analysis of the zeolites and silicalite

Zeolite	Weight %			Atomic Ratios	
	SiO_2	Al_2O_3	NaO	Si:Al	Na:Al
**H-ZSM5-Z-50**	96.4 ± 0.1	3.4 ± 0.1	0.0	23.9 ± 0.9	-
**Na^+-ZSM5-Z-50 (Part Exchange)**	95.7 ± 0.1	3.3 ± 0.1	0.8 ± 0.1	24.6 ± 0.5	0.4±0.1
**Na^+-ZSM5-Z-50 (Full Exchange)**	91.1 ± 0.1	3.6 ± 0.03	2.1 ± 0.01	21.6 ± 0.1	0.9± 0.01
**H-ZSM5-M**	96.3 ± 0.3	3.5 ± 0.3	0.00	23.7 ± 2.1	-
**Na-ZSM5-M**	93.8	4.1	1.7	19.5	0.7
**H-FER-T**	91.3	8.6	0.00	9.1	-
**H-Silicalite**	98.7 ± 0.2	1.3 ± 0.2	0.00	66.9 ± 11.6	-
**Na-Silicalite**	89.0	1.1	2.1	68.7	Na:Si = 0.05
**Na^+ X**	45.8 ± .1	30.8 ± 0.8	21.7 ± 0.4	1.3	1.2

Table 1. X-Ray fluorescence composition analysis of proton and sodium exchanged samples explored in the main text and ESI. ZSM5-Z-50 is the Si:Al ratio 24 Zeolyst sample. ZSM-5-M is a sample originating from Mobil. Fer-T is ferrierite from Tosoh.

The elemental composition of the zeolites and silicalite was determined by X-ray fluorescence (XRF) analysis on a PANalytical Epsilon3 XL spectrometer. Samples were measured as powders using thin-film sample pots. In order to extract the relative proportions of each elemental component, the resulting fluorescence spectra were fitted to reference spectra of SiO_2, Al_2O_3 and various potential contaminants using software and spectral libraries supplied with the instrument. Where reported, the uncertainties are the calculated standard deviations from each sample distributed across 5 - 6 distinct sample pot measurements. Values without standard deviations were performed from a single pot (due to lack of sample). Results are shown in **Table 1**, confirming the overall Si:Al ratios of ZSM-5 and ferrierite and confirming Na^+ ion exchange efficiency. Of particular importance to us was the detection of aluminium in the silicalite sample. We show next with NMR that this aluminium is an Al_2O_3 contaminant and not present within the framework of the silicalite. Note also that the Na:Si ratio of the Na^+ exchanged silicalite is comparable in magnitude with the fraction of Si atoms associated with silanol defects (7%) determined from the ^{29}Si NMR.

NMR spectra of the zeolite samples studied by 2D-IR in the main text were recorded on a Bruker 600 MHz Avance III spectrometer at the University of St Andrews by PhD student Cameron Rice and NMR Facility Manager Dr Daniel Dawson, using a Bruker 4mm low gamma probe. MAS was performed at 14 kHz. Single pulse ^{27}Al spectra were recorded with a 1 μs pulse and referenced to aluminium acetyl

acetonate. Single pulse ^{29}Si spectra were recorded with a 3 μs 90 degree pulse and referenced to Q8M8. ^{1}H cross polarized ^{29}Si spectra were obtained with typically a 5 ms contact time. **Figure S1** shows the ^{27}Al spectra of the ZSM-5 silicalite and ferrierite samples.

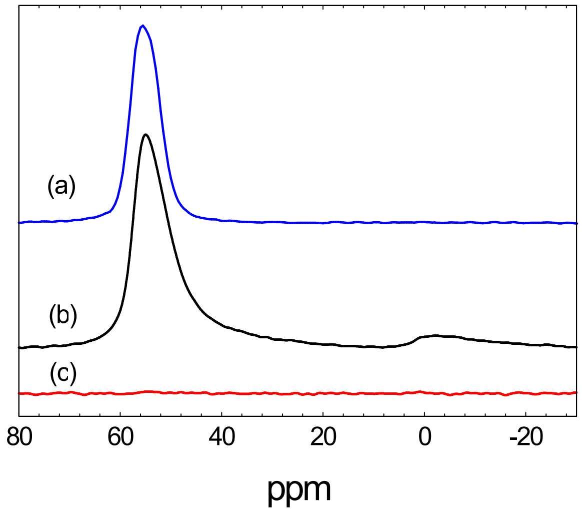

Figure S1 ^{27}Al NMR spectra of the samples studied in the main text. (a) Zeolyst ZSM-5, (b) ferrierite and (c) silicalite.

The ZSM-5 sample shows a single signal characteristic of tetrahedral aluminium at ~55 ppm. In the ferrierite sample there is a very weak signal characteristic of octahedral aluminium at ~0 ppm, indicating the presence of traces only of octahedral extra-framework aluminium. No ^{27}Al signal could be detected from the silicalite sample, indicating the complete absence of both framework (tetrahedral) and octahedral extra-framework aluminium. Although the one pulse ^{27}Al measurement may not be able to detect any aluminium in a lower symmetry environment, the silicalite spectrum nevertheless shows clearly that the silicalite contains no lattice aluminium and therefore has no Bronsted acid sites, as expected. The aluminium detected by XRF in the silicalite sample must therefore be associated with a non-zeolitic alumina impurity.

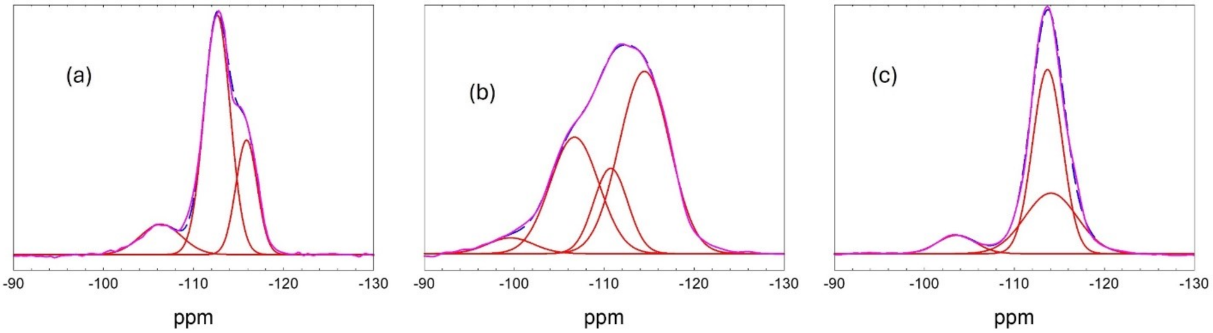

Figure S2. ^{29}Si one pulse spectra of (a) Zeolyst ZSM-5 (b) ferrierite, and (c) silicalite samples. In each figure the purple trace is the measured spectrum, the red traces show the deconvoluted components and the blue dashed trace is the sum of the fitted components.

The one-pulse ^{29}Si spectra are shown in **Figure S2**. These are quantitative (i.e., signal strengths are proportional to composition), and an estimate of the framework Si:Al ratio can be made following Englehardt[1] by fitting the spectra with contributions from silicon atoms coordinated through oxygen to different numbers of silicon, aluminium or hydrogen atoms. For ZSM-5, (**Figure S2(a)**), a satisfactory fit could be achieved with the three components shown. The signal at -106.6 is assigned to $Si(OSi)_3(OAl)$ and the signals at -112.6 and -115.9 are assigned to $Si(OSi)_4$. From the integrated intensities of these signals, the framework Si:Al ratio is estimated to be about 32. The proton cross-

polarised spectrum in **Figure S3(a)** shows a strong signal at -102 ppm which is assigned to silanol defects ($Si(OSi)_3(OH)$). The cross-polarisation measurement enhances all silicon signals in proximity to protons but is not quantitative. The -102 ppm silanol defect signal will also be present in the one-pulse spectrum in Figure S1(a), but cannot be resolved in the available data. Since it overlaps with the -106.6 ppm signal the true framework Si:Al ratio may be higher than the value of 32 estimated above.[2]

We conclude from the NMR measurements that the framework aluminium content and hence the number of Bronsted acid sites present in the ZSM-5 zeolite is certainly lower than that calculated from the XRF analysis (and the Zeolyst specification) of total aluminium content, and no higher than 3 per unit cell.

The one-pulse ^{29}Si spectrum of the ferrierite can be fitted with 4 components (**Figure S2(b)**), at -99.6, -106.7, -110.7 and -114.5 ppm. The assignment of these is somewhat ambiguous. Sarv *et al.* fitted the ^{29}Si spectrum of a ferrierite zeolite with Si:Al = 8.4 to 6 components.[3] A signal at -98.8 ppm was assigned to $Si(OSi)_2(OAl)_2$, at -101.8 ppm to either $Si(OSi)_3(OH)$ or $Si(OSi)_3(OAl)$, at -105 ppm and -108.9 ppm to $Si(OSi)_3(OAl)$, and -110.9 ppm and -115.3 ppm to $Si(OSi)_4$. For a lower aluminium sample (Si:Al = 30) two main signals at -112.2 and -116.6 ppm were assigned to $Si(OSi)_4$ and 4 signals between -103.4 and -109 ppm to $Si(OSi)_3(OAl)$. On the other hand, Bonilla et al. assigned signals at -117.5 and -113.5 ppm to $Si(OSi)_4$, at -110.5 and -106.5 ppm to $Si(OSi)_3(OAl)$, and a signal at -104 ppm to $Si(OSi)_3(OH)$ in a ferrierite with Si:Al = 28.[4] For the ferrierite spectrum in **Figure S2(b)** the most obvious assignment is of the -110.7 and -114.5 ppm signals to $Si(OSi)_4$, the -106.7 ppm signal to $Si(OSi)_3(OAl)$, and the -99.6 ppm signal to $Si(OSi)_2(OAl)_2$. The cross polarised spectrum in **Figure S3(b)** shows no distinct signal due to silanol defects $Si(OSi)_3(OH)$ (unlike the ZSM-5). From the integrated intensities of the signals assigned in this way the framework Si:Al ratio for the ferrierite sample is estimated to be ~10.5, close to that measured by XRF and specified by the supplier.

The one-pulse ^{29}Si spectrum of the silicalite sample (**Figure S2(c)**) shows an intense signal at -114.1 ppm and a second at -103.5 ppm. Similar spectra have been reported for silicalite-1 samples synthesised at low temperatures and containing many structural defects by Palcic et al.[5] The signal at -103.5 ppm is due to $Si(OSi)_3(OH)$ groups associated with silanol defects, confirmed by the prominent signal at this chemical shift in the proton cross-polarised spectrum (**Figure S3(c)**), while the -114.1 signal is due to framework $Si(OSi)_4$ groups (this signal resolves into multiple components in less defective silicalite-1 samples). Curve fitting of the one-pulse spectrum indicates that about 7% of the silicon atoms in this particular sample are attached to an OH group.

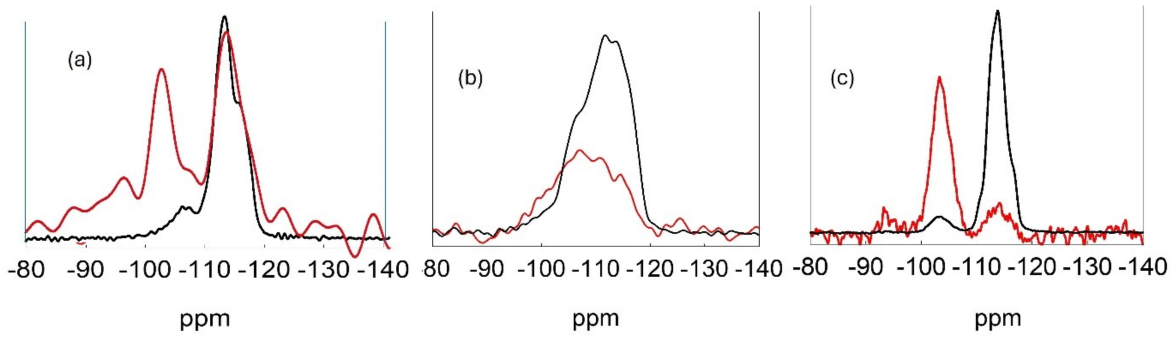

Figure S3. Comparison of single pulse ^{29}Si (black trace) and proton cross polarized ^{29}Si (red trace) NMR spectra of (a) ZSM-5), (b) FER and (c) silicalite.

2. Observing the OD stretch of the deuterated ZSM-5 Bronsted sites hydrogen bonded to D_2O

The zeolite Bronsted site ZOH hydroxyl stretch vibration exhibits striking changes in IR absorption when single water molecules hydrogen bond to the Bronsted site (a configuration referred to here as ZOH…OH_2). The narrow ZOH stretch band at 3600 cm^{-1} (~30 cm^{-1} FWHH) becomes extremely broad, but shows virtually no loss in absorption per unit frequency, as can be seen in **Figure S4(a)**, which shows the 100 ˚C IR absorption spectrum of the Zeolyst sample of ZSM-5 studied in the main text. The origins of the double-peaked structure have been a source of debate over the years, with Fermi resonance of the ZOH…H_2O hydrogen bonded hydroxyl stretch to the hydroxyl bend being the common explanation.[6–9] The recent 2D-IR study by Hack et al argues that the observed 2D-IR spectrum is inconsistent with Fermi resonance and consistent with a double-well potential for the proton coordinate.[10] In this ESI section, we show the form of the IR absorption band for a fully deuterated sample (ZOD…D_2O). It only reveals itself fully after subtraction of the overlapping zeolite lattice overtone bands from 1550-2050 cm^{-1}. We achieve this for a sample at ~120 ˚C using two background methods (1) using a hot, dehydrated zeolite as background and (2) using the 'pellet sandwich' method to obtain a dehydrated zeolite at 120 ˚C, then obtain the ZOD…OD_2 through slow ingress of D_2O.

Figure S4(b) shows IR absorption spectra of deuterated ZSM-5 in an atmosphere of D_2O at two temperatures – 130 ˚C and ~250 ˚C. The ZSM-5 sample was held tightly between CaF_2 windows in order to achieve a uniform temperature across the IR beam in the spectroscopy cell used. The spectra are from a temperature ramp (2 °C / min). We observed that the temperature at which single and no water loading occurred depended on the temperature ramp rate and exact water partial pressures used.

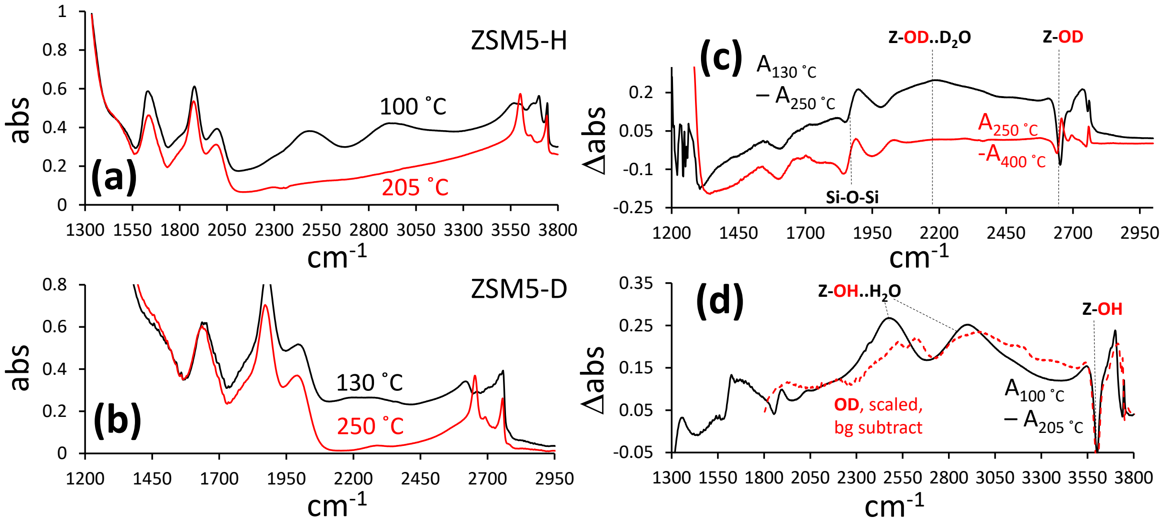

Figure S4. The hydrogen bonded Zeolite Bronsted site OD stretch is clearly observed by calculating the IR difference spectrum of ZSM-5 in N_2 / D_2O vapour at temperatures above the point of water cluster formation and above the point of single-water binding. (a) shows the case for the proton form of ZSM-5 and (b) for the deuteron form. (c) shows the temperature dependent IR difference spectrum for the deuterium case (black) and a 'baseline' difference spectrum recorded for two temperatures above the dehydration temperature of the ZSM-5. Subtracting these and scaling the ZOD…D_2O band to the OH stretch frequency, the ZOD…D_2O band is very similar to the ZOH…H_2O band (d).

In the raw **Figure S4(b)** IR absorption spectra of ZSM-5 in flowing D_2O vapour, although the 130 °C spectrum clearly displays a broad increase in absorption, there is no obvious double-peak structure visible as is the case for the H-stretch spectrum **Figure S4(a)**. The zeolite lattice (Si-O-Si) overtone modes at 1875 and 2000 cm^{-1} obscure the changes. A simple way to partially remove them is to take

a difference spectrum between two temperatures (**Figure S4(c)**, black). The ZOD…D_2O band is then observed to peak at around 2200 cm^{-1} and has a full-width at half maximum approaching 1000 cm^{-1}. Examining the form of the difference spectrum for ZSM-5 with no water at 250 and 400 °C (**Figure S4(c)**, red), we see that from 2000 cm^{-1} and lower, temperature dependent changes in the strong fundamental lattice bands cause a negative offset in the difference spectrum. The derivative features at 1650 cm^{-1} and 1950 cm^{-1} are from the temperature shifting of the lattice overtone modes. Subtracting these features (accounting for the temperature shift), and multiplication of the **S4(c)** frequency axis by 1.355 gives a match of the ZOD and ZOH bands, and we see in **Figure S4(d)** that the ZOH…H_2O and ZOD…D_2O difference spectra are comparable, with even the dip tentatively present.

To more accurately measure the lineshape of the ZOD…OD_2 band, a second method was investigated. This was to measure difference spectra between a dry sample and water-loaded sample at the same temperature. A common means to do this for H_2O loading is with a vacuum system.[8] For the ambient pressure sample cell used in the present studies however, the samples could only be dehydrated by heating. The rate of change in water loading at 1 atm for a pellet cooling to 130 ˚C and exposed directly to even 'dry' N_2 vapour was relatively rapid, preventing the recording of an IR absorption spectrum of dehydrated zeolite at temperatures of 130 ˚C and lower. Holding the zeolite pellet tightly between CaF_2 windows slows the rate of water ingress however, making it straightforward to capture a dehydrated ZSM-5 spectrum at the same temperature as hydrated. These are shown for ZOH…OH_2 and ZOD…OD_2 in **Figure S5(a)**. In both cases the thicknesses of sample are similar and the level of water loading for the difference spectrum chosen such that there remains a small amount of free Bronsted (ZOD/ZOH) absorption. The ZOD…OD_2 spectrum has been frequency scaled by a factor of 1.32, making the match between the hydrogen bonded ZOD…OD_2 and ZOH…OH_2 bands very clear. A match between H and D versions of the non-hydrogen bonded ZOD bands is obtained using a different frequency scaling of 1.355. Neither of these two scalings are what are predicted from the theoretical result obtained from OH and OD reduced masses (1.374).[11] This is presumably because the stretch vibrations involve some motion of the framework Si/Al and bound water.

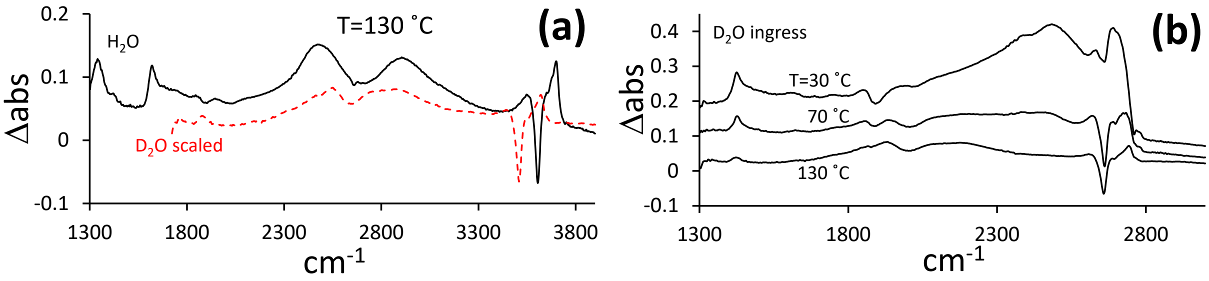

Figure S5. The hydrogen bonded Zeolite Bronsted site OD stretch is clearly and accurately observed by taking the IR difference spectrum of dehydrated and hydrated ZSM-5 at fixed temperature ((a), the D_2O frequency range has been scaled by x1.32). The approach is possible at 1 atm under high humidity when the ZSM-5 sample is held between CaF_2 windows, slowing the ingress of water. (b) Shows that the approach fails below the boiling point of water because clusters of D_2O immediately form on the edges of the pellet, as described in the text.

The success of slowing the ingress of water into ZSM-5 by holding pellets between CaF_2 to obtain **Figure S5(a)** motivated attempts at exploring the ZOD…OD_2 and ZOH…OH_2 bands at lower temperatures. Whereas at 130 ˚C, the ZOD…OD_2 and ZOH…OH_2 spectra were perfectly captured, at lower temperatures, after some minutes, absorption characteristic of protonated water clusters were observed,[8] as is shown in **Figure S5(b)**. We interpret this interesting effect as follows. A dehydrated ZSM-5 pellet ~6-9 x 6-9 mm in dimension, pressed between glass can only adsorb water from its edges. The IR spectrometer measures an area of sample *in the middle* of the pellet of ~4 mm diameter. Dropping the temperature of the ZSM-5 pellet to 30 ˚C and waiting for the water to appear in the

centre of the pellet, *only* protonated clusters were ever observed in the IR absorption spectrum. For the 30 ˚C spectrum in **Figure S5(b)**, though the ingress was gradual, the first signs of weak IR absorption were from protonated clusters reaching the dehydrated pellet centre about a minute after the sample reached 30 ˚C. The spectrum shown is after about 10 minutes. At no point were the doublet ZOD…OD_2 and ZOH…OH_2 bands observed. This implies that at 30 ˚C, when protonated water clusters immediately form at the edges of the pellet, they prevent diffusion of single water molecules further into the pellet, and progress into the pellet as clusters. At 70 ˚C, a mixture of cluster and ZOD…OD_2 IR absorption is observed in **Figure S5(b)**, indicating that water 'vapour' diffusion into the centre of the pellet still occurs.

3. 2D-IR of water in ZSM-5: sample conditions, HOD/H_2O and D_2O 2D-IR spectra and waiting time dependence

In an atmosphere of water and N_2, the transition from dehydrated zeolite to single water molecule per acid site loadings is easy to monitor by IR absorption spectroscopy, and easy to control through temperature and by slowing water ingress rate using CaF_2 encapsulation, as was shown in **ESI Section 2**. Lowering the zeolite temperature below ~120 °C results in protonated water clusters forming around each acid site. Sites at channel intersections accommodate the highest number of water molecules with the maximum number of molecules *n* per cluster estimated in these locations thought to be *n* ~ 4 - 9 for ZSM-5.[8] Defect-free silicates are relatively hydrophobic, and ZSM-5 ought to capture water only around its aluminium substitutions up to the limit imposed by their density and pore/intersection size. When ZSM-5 contains defects however, a sample will continue to absorb water well beyond this level. This regime is characterised by an indistinct hydroxyl stretch IR absorption band.

Cooling a dehydrated sample of ZSM-5 and simultaneously observing the IR absorption spectrum, the distinctive form at n ~ 1 loadings of the ZOH…OH_2 doublet is observed (**ESI Section 2**). The Bronsted site is deprotonated for n > 2 water molecules. Then, the doublet band no longer contributes to the spectrum at all. As a sample continues to cool, protonated clusters grow, displaying a spectrum with characteristic broad low and narrower high frequency bands. These intensify as water further adsorbs, finally giving way to an indistinct form at highest loadings. Following the interpretation of the IR absorption spectra of the protonated cluster regimes presented by Hack et al,[12] we loosely assign the loading of the main text **Figure 6(a)** IR absorption and 2D-IR spectra as n ~ 6 - 12 water molecules per acid site. At any higher loading than this, the high frequency band no longer appears prominently in the spectrum.

Clusters formed in the 100 μm thick free-standing pellet samples used to generate **Figures 6(b)** and **(c)** (main text) were highly IR absorbing (OD > 1.5). Interestingly, in the 2D-IR setup, laser heating a sample under these conditions would rapidly remove this water, consistently and stably maintaining D_2O loadings at ~1 molecule per acid site. In order to observe the protonated water clusters in ZSM-5 pellets by 2D-IR (**Figure 6(a)** main text), the sample optical density and laser heating needed to be reduced by preparing samples 4-6x thinner than those used for studies at lower hydration. This was achieved by twisting / pressing smaller amounts (1-2 mg) of zeolite directly between the CaF_2 windows of the spectroscopy cell. With care, a uniform spread of sample could be achieved. These samples were slightly more scattering than the hydraulically pressed pellets, but the lower optical density and direct thermal contact with CaF_2 resulted in less laser absorption and heating. **Figure S6** shows 2D-IR spectra of these thin samples of ZSM-5 containing respectively protonated D_2O (reproduced from the main text **Figure 6(a)**) and HOD/H_2O clusters at ~45 °C. The broad absorptions at ~2500 cm^{-1} and at ~2675-2700 cm^{-1} correspond to separate distributions of stronger/more and weaker/less hydrogen

bonded deuteroxyl groups. The question of why there is simply not a continuous absorption like in the bulk liquid[13] is interesting, and presumably due to the constraints to hydrogen bonding imposed by the zeolite.

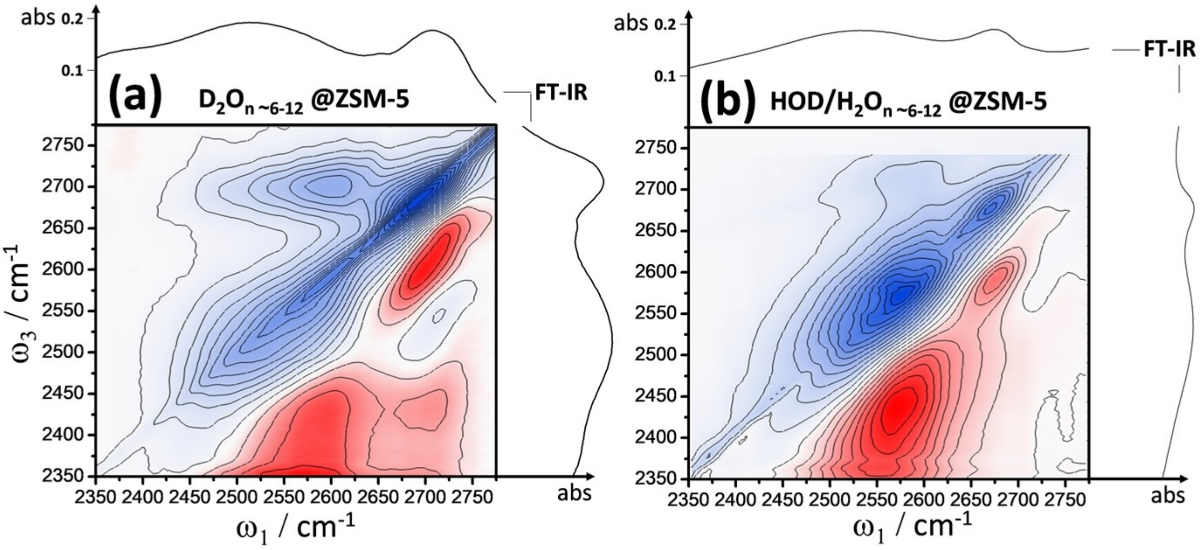

Figure S6. A comparison between ZSM-5 samples at 40 ˚C containing (a) pure D_2O (same figure from main text **Figure 6(a)**) and (b) 20% volume D_2O in H_2O. The samples were held tightly between CaF_2 windows and after drying and H-D exchange, cooled to ~40 °C and equilibrated in a stream of (a) N_2 / D_2O vapour and (b) N_2 / HOD / H_2O vapor. The 2D-IR spectra were recorded at a waiting time t_2 ~ 150 fs with crossed pump and probe polarisation <XXYY>.

In their interpretation of stretch mode ν(OH) ZSM-5 protonated water cluster 2D-IR spectra, Hack et al suggested that the small clusters are branched, and comprise a heterogeneous distribution of strongly coupled, non, or weakly hydrogen bonded (high frequency, HF) water, and singly and doubly hydrogen bonded water at lower frequency (LF). The strong cross peaking in the 2D-IR spectrum of **Figure S6(a)** must be due to strong intra-molecular and weaker inter-molecular vibrational coupling across the clusters. We examined a ZSM-5 sample with the D_2O replaced with HOD/H_2O (**Figure S6(b)**, 20% D_2O in H_2O) and observe again, LF and HF distributions. Interestingly a (weaker) direct coupling cross peak is present, even at early waiting time. This cannot be due to residual D_2O molecules, which for the 1:5 D_2O/H_2O mixture used would be 3% of the total water.[14] With the cluster sizes in the samples studied thought to be n~6-12, each cluster may therefore contain ~1-2 HOD molecules. It is possible that the weak cross peak in **Figure S6(b)** is therefore the coupling between two OD stretch groups across the network of hydrogen bonds within one cluster, as is observed in the isotopically pure case. Alternatively, it is perhaps very rapid H-bond interconversion (see below).

Figures S7, S8 and **S9** show waiting time dynamics for the ZSM-5 HOD/H_2O cluster (**S7**), D_2O cluster (**S8**) and single D_2O loading (120 ˚C, **S9**) 2D-IR spectra. **S8** and **S9** correspond to **Figure 6(a)** and **(b)** of the main text. Interestingly, for the HOD/H_2O cluster (**S7**) data, upon an increase in waiting time, the HOD cross peak shifts and appears to give rise to a new cross peak between the low and high frequency distributions which grows with waiting time. No such behaviour occurs for the D_2O cluster (**S8**) or single D_2O loading (**S10**) 2D-IR spectra. It is possible that the cross peak growth is dynamic structural exchange between the two distributions of hydrogen bonded modes. Further study is required to discount the alternative explanation of vibrational energy relaxation from the one distribution perturbing the other.[15]

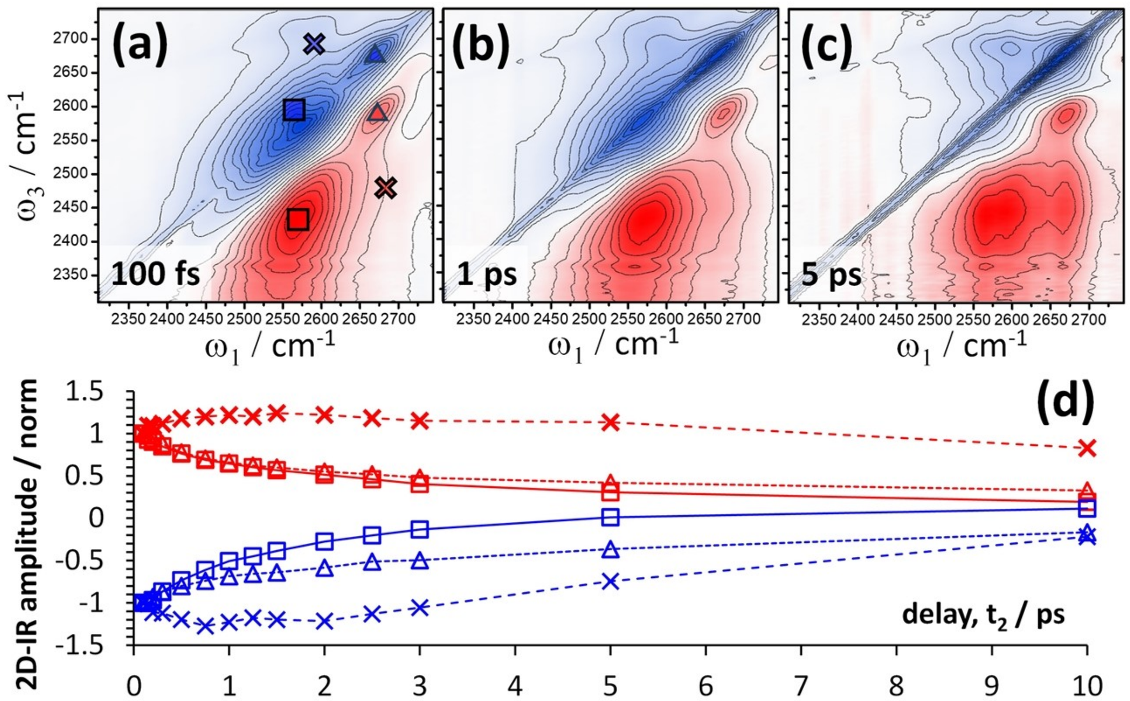

Figure S7. A thin ZSM-5 sample containing HOD/H_2O clusters at ~40 °C displays spectral diffusion and cross peak growth as a function of waiting time t_2. (a) - (c) show representative 2D-IR spectra at 100 fs, 1 ps and 5 ps waiting times. The amplitude dependence as a function of waiting time at positions in (a) marked by symbols (cross, circle and square) are shown in (d), normalised to the value at 0 ps.

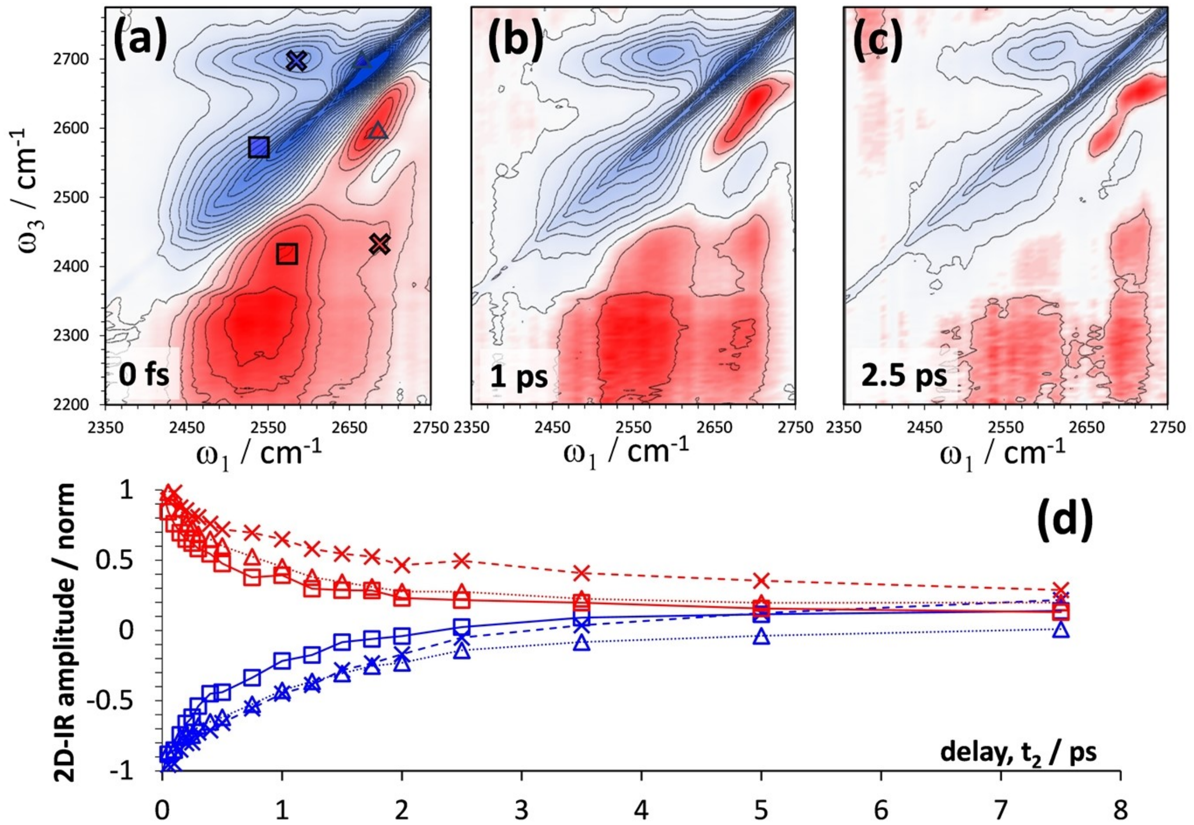

Figure S8. A thin ZSM-5 sample containing D_2O clusters at ~40 °C shows monotonic relaxation for on-diagonal and cross peak regions of the 2D-IR spectrum as a function of waiting time t_2. (a) - (c) show representative 2D-IR spectra at 0, 1 and 2.5 ps waiting times. The amplitude dependence as a function of waiting time at positions in (a) marked by symbols (cross, circle and square) are shown in (d), normalised to the value at 0 ps.

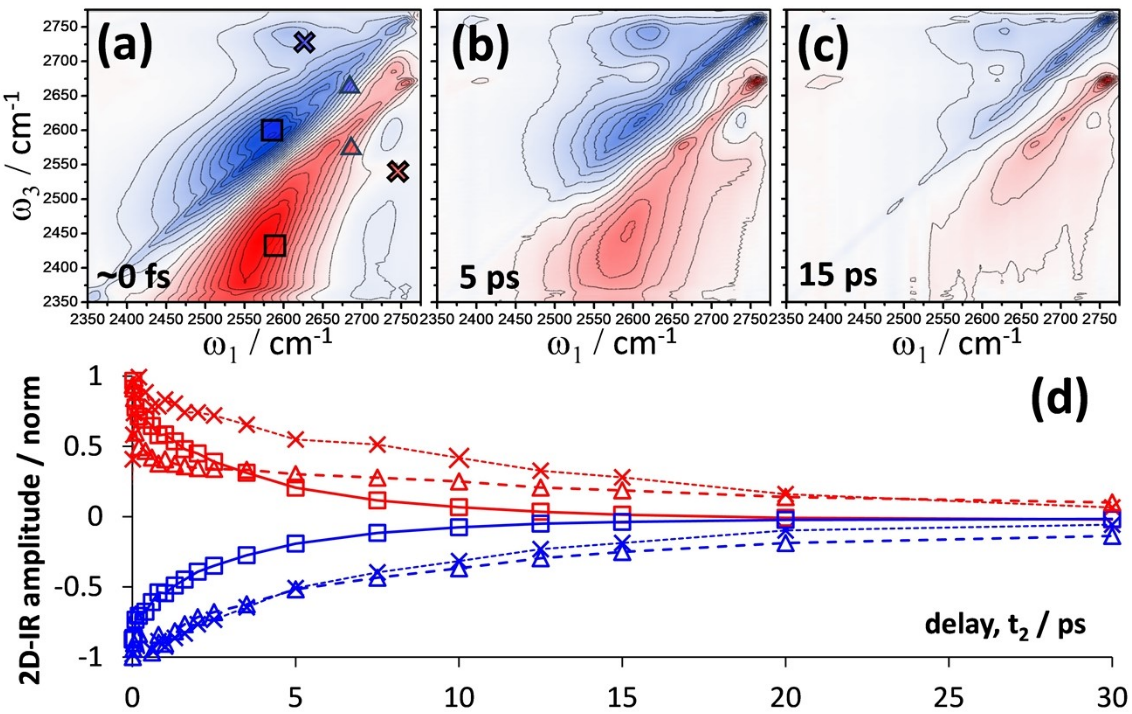

Figure S9. A ZSM-5 sample in a flow of N_2 / D_2O vapour at ~120 °C shows monotonic relaxation on-diagonal and in cross peak regions of the 2D-IR spectrum as a function of waiting time t_2. (a) - (c) show representative 2D-IR spectra at 400 fs, 5 and 15 ps waiting times. The amplitude dependence as a function of waiting time at positions in (a) marked by symbols (cross, circle and square) are shown in (d), normalised to the value at 0 ps.

In interpreting the 2D-IR spectra of **Figures S7-9** and their counterparts in the main text, it is important to note that the pump bandwidth is finite, and this affects the appearance of the spectra below 2500 cm^{-1}. In principle, the time zero 2D-IR spectra should contain considerable intensity from the Z<u>OD</u>…OD_2 or proton complex modes at low wavenumbers. Because of the limited pump bandwidth, the signal intensity drops to zero below 2400 cm^{-1}.

Fewer HOD/H_2O experiments than D_2O were performed on ZSM-5 at temperatures above that at which water cluster decomposition occurs. **Figure S10** shows a comparison of a ZSM-5 pellet in N_2/D_2O and N_2/HOD/H_2O (N_2 sparged through 20% D_2O in H_2O). The temperatures between the pure D and the H/D measurements are not the same. The data are also disturbed somewhat by atmospheric CO_2 absorption in the probe beam at 2375 cm^{-1}. We nevertheless conclude that the nest band at 100% D (**Figures S10(a)** and **(b)**) looks very similar to the nest band at 20% D (**Figures S10(c)** and **(d)**).

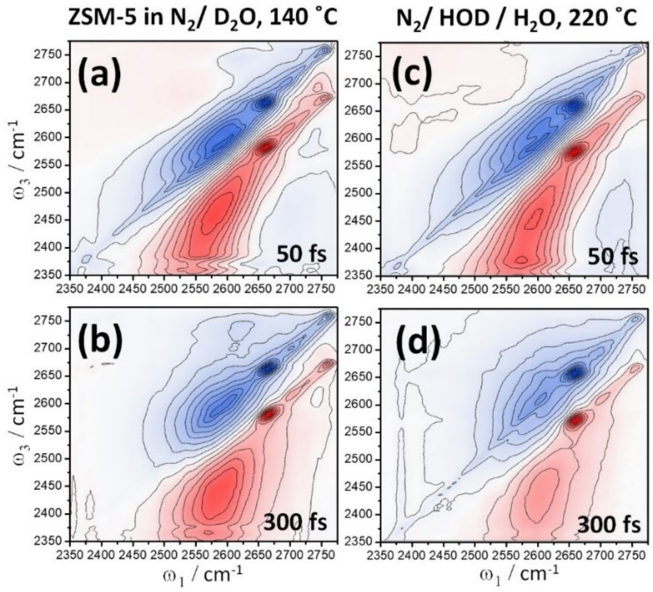

Figure S10. 2D-IR spectra of of ZSM-5 held at (1) ~140 ˚C in flowing D_2O / N_2 vapour (a) t_2 = 50 fs, (b) t_2 = 300 fs and (2) 220 ˚C in HOD / H_2O /N_2 vapour (c) t_2 = 0 fs, (d) t_2 = 300 fs. Crossed polarisation <XXYY> was used.

4. The 2D-IR cross peaks from ZSM-5 bound water disappear as a function of temperature

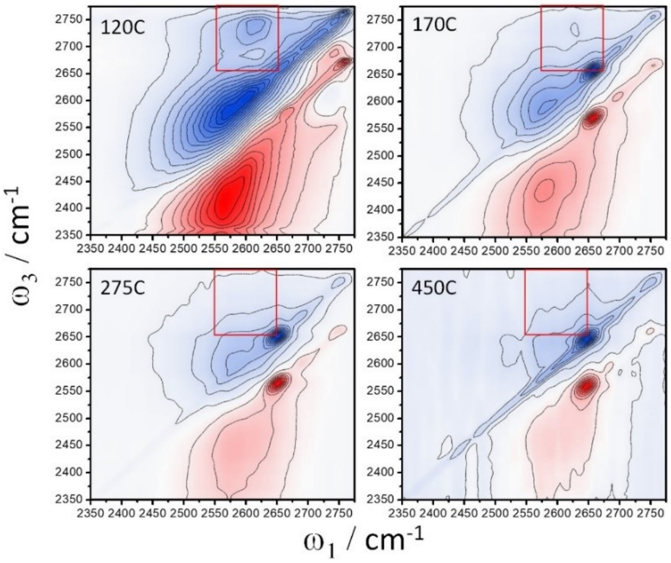

Figure S11. 2D-IR spectra of ZSM-5 in a flow of N_2 / D_2O at four temperatures. <XXYY> pump-probe polarisation, t_2~400 fs

Figure S11 shows spectra of ZSM-5 in a flow of N_2 / D_2O at four temperatures from 120 ˚C to 450 ˚C. The cross peaks clearly disappear with increasing temperature, strongly supporting the assignment of water. An obvious way of making the temperature dependence quantitative is to monitor the spectral changes at increased temperature resolution, as is typically done for temperature programmed desorption measurements. In **Figure S12**, we show such a measurement, but instead of using 2D-IR spectroscopy, we used IR pump-probe spectroscopy. IR pump-probe measurements are quicker to perform, and easily collected with no change to a 2D-IR setup. **Figure S12(a)** shows a series of pump-probe spectra (t_2=500 fs) plotted as a function of temperature. The cross peak bleach is clearest at 2725 cm^{-1}. Also clear are the ZOD bleach (2615 cm^{-1}), silanol nest bleach and ESA at 2625 cm^{-1} and 2400 cm^{-1} respectively. The ZOD ESA band is at 2560 cm^{-1}. Setting the signal at the edges of these

bands to zero (baselining) and plotting their integrated amplitude as a function of temperature, we obtain **Figure S12(b)**. On increasing temperature, the water cross peak amplitude decays in exact proportion to the growth of the free Bronsted ZOD feature at ~2650 cm^{-1}, as expected for the conversion of ZOD…OD_2 to ZOD. The silanol nest signal is only partially integrated across its full band width, which nevertheless gives a good measure of its intensity, which decreases as a function of temperature.

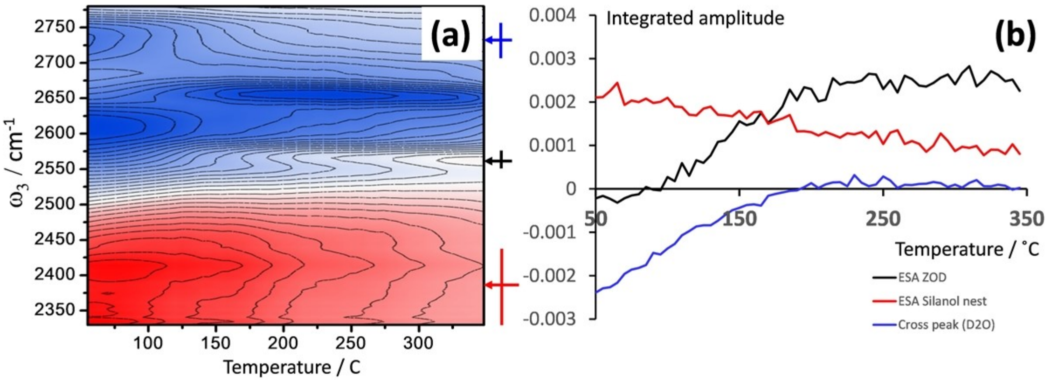

Figure S12. Pump-probe spectra (B, t_2=500fs) of ZSM-5 in flowing N_2/D_2O taken rapidly as a function of temperature (30 second average per spectrum, ramp rate of 10 ˚C / min). The integrated band intensities (baselined) as a function of temperature are shown in (b) for the acid site (ZOD, ESA), the water cross peak and silanol nest ESA) discussed in the text. In (a) blue=negative signal, Red=positive. The arrows and lines point to the regions integrated over.

5. Exploring zeolite water cross peaks using 2D-IR polarisation ratios

The anisotropy, or ratios of 2D-IR cross peak signals at perpendicular and parallel pump-probe polarisation depend on the angles between the contributing vibrational mode transition dipoles.[16] Previous applications of this property are the characterisation of the symmetric/asymmetric character of the OD stretch spectrum of liquid D_2O,[13] and of protonated water clusters in oil index matched mulls of ZSM-5 at room temperature.[12] The present 2D-IR experimental approach[17] enables surprisingly good quality 2D-IR spectra of pressed pellets to be collected under variable temperature and gas flow in both the perpendicular (<XXYY>) and the more problematic parallel geometry (<XXXX>), which is significantly affected by scattered pump light. We use the ratio of the perpendicular and parallel 2D-IR spectral amplitudes to highlight the location and shape of cross peaks, and also potentially gain information about transition dipole angles. Compared to other similar quantities such as the anisotropy, the perpendicular to parallel signal ratio has similar properties, but staring at live data in the lab, it is slightly simpler to quickly calculate a ratio, which ranges from 0.33 to 1.17. The anisotropy ranges from -0.2 to 0.4.

The largest theoretical polarised <XXYY>/<XXXX> signal ratio for a cross peak from two coupled vibrations is observed at a transition dipole angle of 90°, giving a 2D-IR cross peak amplitude ratio of 1.17. For reference, aligned transition dipole cross peaks and strong 2D-IR diagonal peaks (both having a zero degree relative angle between pumping and probing) display a polarisation ratio of 0.33. Dipoles oriented at the water geometric bond angle 104.5° would display a polarisation ratio of 1.03. If water modes are split as symmetric/asymmetric stretches (implying similar local mode frequencies and similar H-bond donation on both sides of the molecule), the transition moment angle will be 90˚. If the water molecule modes are bond-local (implying different local mode frequencies and different hydrogen bond strengths either side of the molecule), the angle may be closer to the water structural angle of 104˚. We will discuss later in this section to what extent this will be true, and whether it can be deduced from the available data.

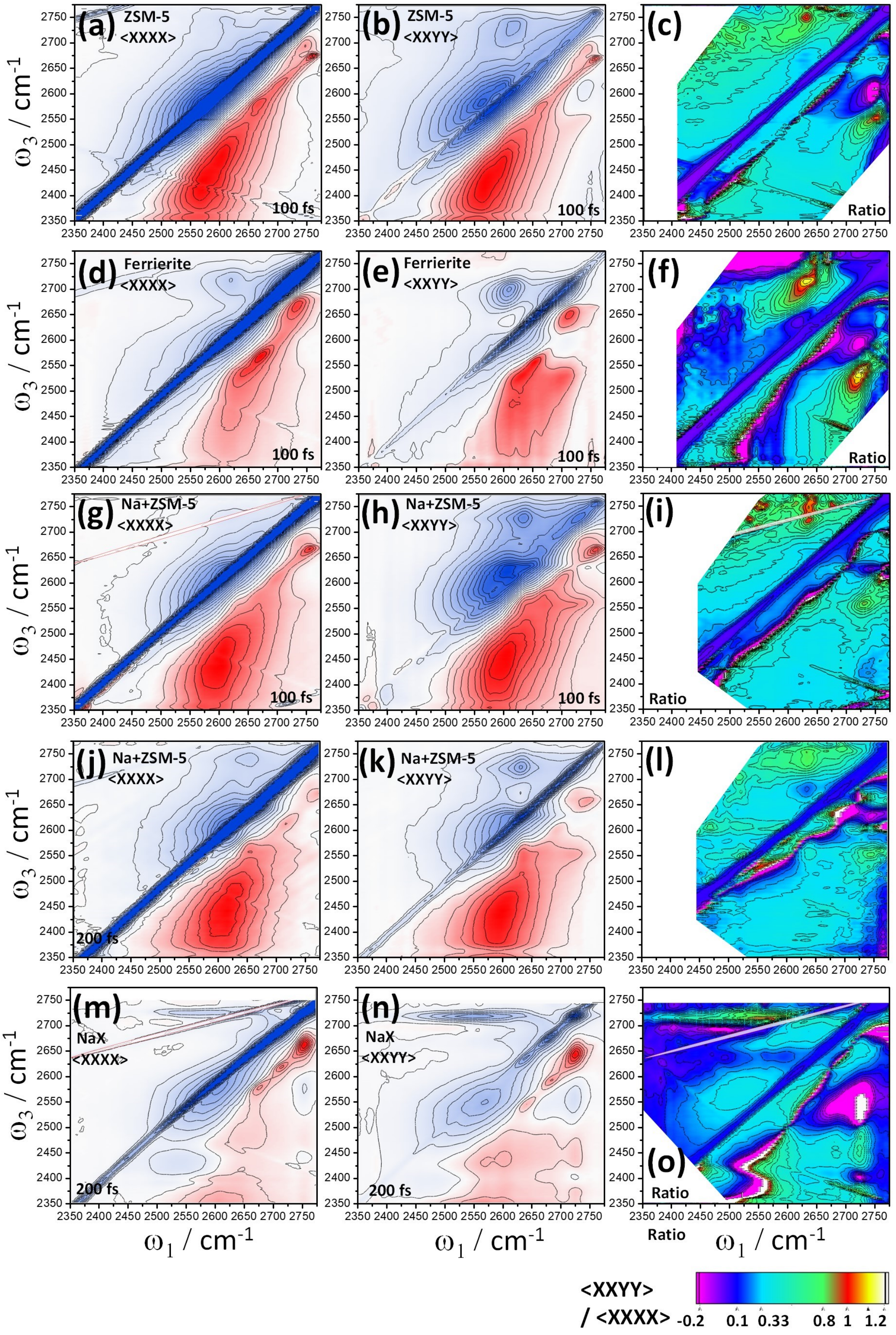

Figure S13. 2D-IR spectra measured in parallel (<XXXX>, left column, (a), (d), (g), (j), (m)) and perpendicular (<XXYY>, middle column, (b), (e), (h), (k), (n)) pump-probe polarisation, and their ratio (right column, (c), (f), (i), (l), (o)). The samples are deuteron form ZSM-5 (120 °C, (a)-(c)), ferrierite (120 °C, (d)-(f)), partially Na^+ exchanged ZSM-5 (220 °C, (g)-(i)) and (180 °C, (j)-(l)) and Na^+ zeolite X (~220 ˚C, (m)-(o)). All samples were held in flowing D_2O / N_2 vapor and recorded with t_2=100 fs or 200fs (as indicated). Baseline corrections were applied to the below-diagonal side polarisation ratios to correct for errors (for the raw data, see **Figure S15**). The extra diagonal line in (g) and (m) and their ratios is a combination of pump scatter and pulse-shaper artefact.

Polarisation ratio measurements are affected by spectral congestion and backgrounds at earliest waiting times (pulse overlap) and at longer waiting times by vibrational relaxation and thermalisation. These tend to reduce the numerical value of the cross peak signal ratio, and cause some striking looking artefacts to appear in the 2D ratio spectra. For example, a strong 2D-IR cross peak of opposite sign to a background of 2D-IR diagonal signal will have a negative ratio and potentially exhibit sudden sign changes. We discuss these effects in detail in the next section.

Polarisation dependent 2D-IR spectra and their ratios are shown in **Figure S13** for

(1) ~single loaded D_2O in ZSM-5 at 120 °C, **S13(a)-(c)**,

(2) ~single loaded D_2O in ferrierite at 120 °C, **S13(d)–(f)**

(3) two examples of D_2O in partially Na^+ exchanged ZSM-5 at 180 °C, **S13(g)-(i)** and at 230 °C **S13(j)-(l)**

(4) D_2O in Na^+ Zeolite X at 230 ˚C **S13(m)-(o)**.

S13(c), (f) and **(o)** are shown in **Figure 8** of the main text. All samples were held in a flow of N_2 / D_2O vapour. Despite the strong diagonal scatter, the cross peaks appear clearly in all 2D contour plots of the ratio. Cross peaks farthest from the diagonal exhibit the least disturbance in ratio value from the diagonal signals. In all cases, the cross peaks and cross peak ratios are clearest in the 'above-diagonal' section of the 2D-IR spectra. This is due to being farthest from the interfering on-diagonal 0->1 bleach-SE compared with the cross peaks below the diagonal, which are comparatively more overlapped with the broad, anharmonically shifted 1->2 ESA.

The 2D-IR polarisation ratio cross peaks of ZSM-5 and ferrierite in **Figures S13 (c), (f)** and **(i)** are intriguingly structured. Narrow structures are reproducibly observed exhibiting interstate coherence oscillations[18] as a function of waiting time. The fact that the small, narrow, peaks are symmetric about the diagonal also indicates that they are not artefacts. They align to the narrow peaks of non-hydrogen bonded modes on-diagonal. The 2D-IR polarisation ratio therefore provides contrast for observing these non-hydrogen bonded modes.

The structural diversity of ZSM-5 adsorbed water sensed by 2D-IR becomes more apparent upon change of cation to Na^+, as shown in **Figure S13 (g)-(i)** (220 °C sample) and **S13(l)-(k)** (180 °C sample recorded on a separate occasion). In these particular Na^+ exchanged samples, the temperature and exchange time were only sufficient to exchange Bronsted protons, and not the silanol nests (see **Table 1** in **ESI Section 1**). There are nevertheless reproducible changes in cross peak structure for both the <XXYY> and ratio spectra compared with the deuteron form of the ZSM-5.

The 2D-IR cross peaks and ratio signals are largest for ferrierite (**Figure S13 (d)**-**(f)**). The cross peak polarisation ratio maximum is around 1.2, the strongest indication that there might be a strong contribution from coupled modes with transition dipole angles oriented in the region of 90°. The problem with this conclusion is that the frequencies of the two modes contributing to the strong cross peak of ferrierite are consistent with the coordinating water molecule being only singly hydrogen bonded to the Zeolite framework. It is therefore tempting to designate the resulting ν_{LF} and ν_{HF} modes as having a local mode transition dipole angle equal to the water geometrical angle of 104.5°. However, the very existence of the strong cross peak in the 2D-IR spectrum indicates that the modes are relatively strongly coupled. Anharmonic vibrational coupling by definition causes mixing of the two bond-local modes and probably results in transition dipoles closer to orthogonal. Local mode mixing is qualitatively supported by anharmonic frequency calculations of water in ZSM-5.[19] Another factor of note is that the local mode hydrogen bonded OD stretch transition dipole of adsorbed water itself may not necessarily lie along the bond. Although the ferrierite cross peaks are very strong, and

therefore free from overlap with the diagonal bands, it is uncertain whether the ratio is slightly over/under-estimated, making it difficult to distinguish 90° and 104.5° transition dipole geometries. The values of the ratio in ZSM-5 are especially impacted by the presence of the strong, broad nest feature at $\omega_1 = \omega_3$ =2575 cm^{-1}. Simple simulations in the following **ESI section 6** shows that this lowers the apparent value of the ratio.

The following two questions therefore remain open: (1) are the two coupled modes of water orthogonal in transition dipole direction, as is the case for two identical frequency coupled water OD modes? Or, are the two coupled modes more 'bond-local', as would be the case for two local water OD modes with different frequencies? (2) Can we use these properties in structural studies? Or are both the theoretical limitations and experimental uncertainties too great?

6. Factors to consider when interpreting 2D-IR polarisation ratio spectra

Three different ratios of perpendicular <xxyy> and parallel <xxxx> 2D-IR signals *S* can be used to obtain estimates of cross peak transition dipole angles and make 2D plots. They are all related, and only 'exact' under the assumption of random molecular ordering, a clear separation of the cross peak of interest from diagonal signals, a clear separation of the negative and positive components of the cross peak, no rotational motion occurring over the timescale of the 2D-IR pulse sequence and no polarisation dependent vibrational energy relaxation effects.

The first common ratiometric quantity is:[16]

$$\frac{S_{xxxx}(\omega_1,\omega_3)}{3S_{xxyy}(\omega_1,\omega_3)} = \frac{4P_2(\theta_{ab}) + 5}{10 - P_2(\theta_{ab})}$$

Eq. S1

Here, θ_{ab} is the angle between the transition moments of two vibrational modes *a* and *b*. $P_2(\theta_{ab})$ is the second Legendre Polynomial and equal to $\frac{1}{2}(3\cos^2\theta_{ab} - 1)$.

Another common and related ratiometric quantity is the *anisotropy*:

$$\frac{S_{xxxx}(\omega_1,\omega_3) - S_{xxyy}(\omega_1,\omega_3)}{S_{xxxx}(\omega_1,\omega_3) + 2S_{xxxx}(\omega_1,\omega_3)} = \frac{2}{5}P_2(\theta_{ab})$$

Eq. S2

We find the simple ratio of perpendicular to parallel signal to be useful:

$$\frac{S_{xxyy}(\omega_1,\omega_3)}{S_{xxxx}(\omega_1,\omega_3)} = 3\frac{10 - P_2(\theta_{ab})}{4P_2(\theta_{ab}) + 5}$$

Eq. S3

Figure S14 shows **Eq S1. - Eq S3.** plotted as a function of θ_{ab} . The strong diagonal signals which typically dominate a 2D-IR spectrum are the result of pumping and probing the same vibrational mode, therefore $\theta_{ab} = 0$. The perpendicular to parallel ratio (**Eq. S3**) increases in value for cross peaks as θ_{ab} increases. The scaled reverse ratio (**Eq. S1**) and anisotropy (**Eq. S2**) reduce in value. The perpendicular to parallel ratio of **Eq S3.** is slightly more peaked as a function of angle at around 90˚. Although the differences are not dramatic, the perpendicular to parallel ratio of **Eq. S3** may be slightly more advantageous than both its inverse and anisotropy in terms of dynamic range and sensitivity to angle changes around 90˚. As **Eq. S1.** involves division by the weaker perpendicular spectrum, this ratio

exhibits more experimental noise in regions where the signal is approaching the noise floor. We will focus on understanding 2D-IR polarisation dependence of the cross peak signals using the ratio of perpendicular to parallel 2D-IR signal (**Eq. S3**).

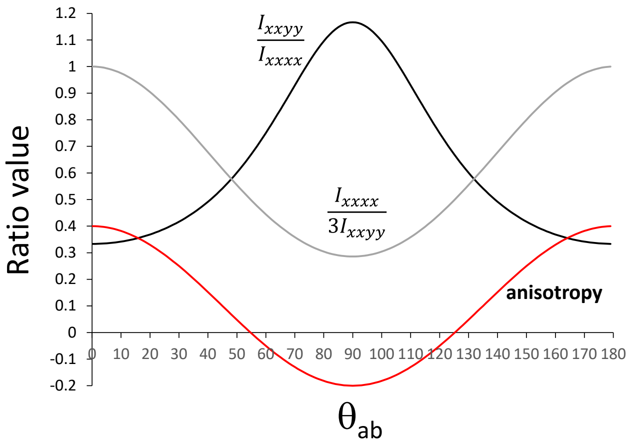

Figure S14. Theoretical values for three kinds of parallel and perpendicular 2D-IR signal ratios as a function of the angle θ_{ab} (in degrees) between a pumped vibrational transition dipole *a* and the probed transition dipole *b*.

In the measurements of **Figure S13**, the parallel and perpendicular polarised 2D-IR spectra were collected simultaneously on separate 128 pixel array detectors. The spectra were x2 interpolated and small frequency shifts were applied to the parallel array to ensure that all sharp features, such as scatter and silanol bands lined up in probe frequency in the parallel and perpendicular datasets. The 'raw' ratios calculated from each perpendicular polarisation 2D-IR signal pixel were divided by the corresponding parallel polarisation pixel. The examples of ZSM-5 and ferrierite n~1 water are shown in **Figure S15**. The curious node across the cross peaks in the lower right quadrants indicates an error. These errors occurred in the experimental data no matter which ratio (**Eq. S1. - S3.**) is used.

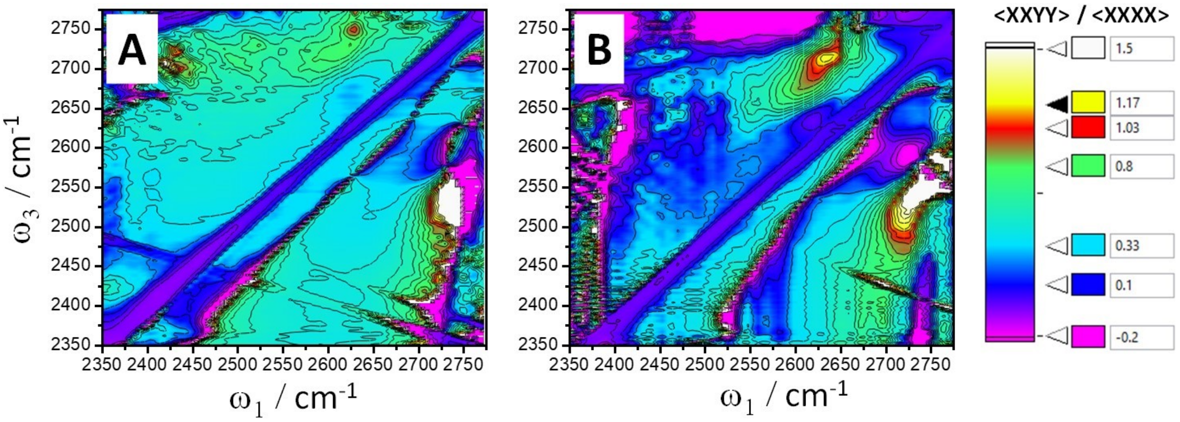

Figure S15 'Raw' polarisation ratios for ZSM-5 (A) and Ferrierite (B), revealing the appearance of an artefact in the lower quadrant. The 2D-IR polarisation ratios of Figure S13 have a small negative offset applied to the lower right quadrant to remove this.

To understand the essential features of the parallel and perpendicular ratio / anisotropy 2D-IR spectra, and the errors in **Figure S15**, we simulated parallel and perpendicular 2D-IR spectra using 2D Gaussians with adjustable amplitudes, widths, anharmonicity values and diagonal correlation coefficients. A total of 8 Gaussian functions were used to describe the 2D-IR spectrum of 2 coupled modes with parameters similar to the ferrierite ν_{LF} and ν_{HF} bands in main text **Figure 6** and **Figure S13** ferrierite 2D-IR spectra.

Figure S16 shows an example sequence of model simulations. The cross peaks are scaled to be x3 weaker (upper quadrant) and x7 times (lower quadrant) weaker than the diagonal bands for both polarisations. Between parallel and perpendicular polarisations, amplitude ratios of 0.33 for diagonal features and 1.17 for the cross peaks are enforced. For the simulations of **Figure S16(a)** and **(b)**, as the spectra are 'perfect' in a mathematical sense (the correct amplitude ratios are maintained out to infinite frequency), away from interference from the diagonal bands, the polarisation ratio value of 1.17 (coloured yellow) extends far from the cross peaks. The cross peak regions closest to the diagonal (upper left quadrant positive cross peak, lower right quadrant, negative cross peak) display a ratio value of 1.17 at-centre, but are altered by the opposite-signed diagonal signal elsewhere. From centre, the ratio rises above 1.17, then falls negative. Focussing on the upper left quadrant cross peak, the sign change happens here because the perpendicular signal cross peak amplitude is positive but the equivalent parallel signal is negative (this is because the positive 'ESA' cross peak signal in parallel polarisation is overlapped with proportionally stronger overlapping diagonal bleach). Making the diagonal bands 5x brighter (**Figure S16 (b)**) makes this effect more pronounced.

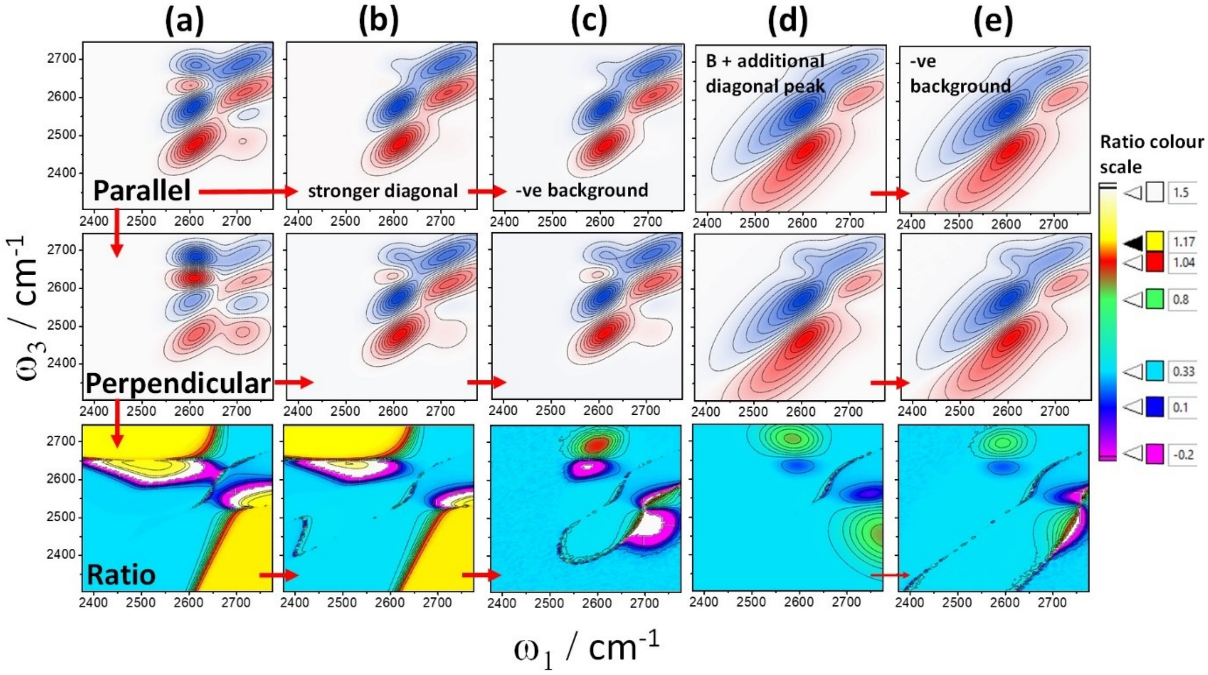

Figure S16. Simulating parallel and perpendicular 2D-IR spectra and their ratios using 2D Gaussian functions. The <XXYY>/<XXXX> ratios of the cross peaks have been set to 1.17 and the diagonal peaks 0.33. The approximate form of the Figure S13 parallel and perpendicular Ferrierite 2D-IR spectrum is mimicked in **(a)**. In **(b)**, the diagonal bands are made 5x stronger. In **(c)**, a uniform negative background 1.5% of the strongest bleach, and fluctuating by 10% has been added. In **(d)**, an additional, broad diagonal feature has been added to **(b)**. **(e)** is the sum of the effects of **(b), (c)** and **(d)**.

In a real measurement, backgrounds and noise will be present in 2D-IR spectra. One possible cause of backgrounds are when the waiting time t_2 is too small. Then, the 2D-IR spectra have contributions from the perturbed free-induction decay and nonresonant signal. This was observed in some of the experimental polarisation dependent 2D-IR spectra for $t_2 < 100$fs. Other backgrounds can be from the presence of extremely broad spectral features, such as proton continuum absorption bands, or from some proportion of the sample returning rapidly to a hot ground state. In the presence of any backgrounds, 2D-IR polarisation ratio values will only be valid for regions where the cross peak signals greatly exceed these backgrounds. To simulate this, **Figure S16(c)** uses the same peak parameters as simulation **Figure S16(b)**, but has an added, uniform negative signal at 1.5% of the amplitude of the strongest diagonal feature of **Figure S16(b)**. The background is anisotropic, and so 3x stronger for the

parallel case (a ratio of 0.33). To simulate the noise of real data in the simulation, both <XXXX> and <XXYY> polarisation 2D-IR spectra used to calculate the ratio have an added 10% random fluctuation in value.

Focussing first on the above-diagonal cross peak, several interesting effects can be seen in the simulated polarisation ratio 2D spectrum **Figure S16 (c)** that are also observed in the experimental data of **Figures S13** and **S15**. Firstly, by adding backgrounds, the polarisation ratios become more localised, and so more like the cross peaks of ordinary 2D-IR spectra. This is because the added background ratio of 0.33 dominates in regions far from the cross peaks. Secondly, the ratio of the bleach cross-peak is reduced from 1.17 to around 1, i.e. it is being 'pulled' closer to the background value of 0.33. Thirdly, the lower companion excited state absorption cross-peak no longer reflects the true amplitude ratio at all – it is ~-0.2! This is an extension of the effect observed in **S16 (a)** and **(b)** - the perpendicular 2D-IR signal is still positive on this cross peak, but the parallel signal, where the diagonal signal and background dominate the positive cross peak signal is negative, making the ratio small and negative, whilst still retaining the structure of the cross peak in shape.

The polarisation ratio 2D spectrum in **Figure S16 (c)** has a striking looking artefact because of the added background. A node appears along the lower right quadrant cross-peak. This is occurring at the point that the sign of the ratio flips. The sign flips are opposite for the bleach cross peak and excited state absorption cross peak. For the bleach cross peak, this is simply due to the overlapping positive diagonal signal being dominant in parallel polarisation near the diagonal, but eventually giving way to negative bleaching (where the sign flips). For the excited state absorption cross peak, traversing from left to right in pump frequency across the peak, the 2D-IR signal ratio's sign changes from positive to negative when the added negative background of the parallel polarisation becomes dominant in the ratio. A node across a cross peak in an anisotropy or polarisation ratio spectrum is therefore a clear experimental indicator that there are backgrounds in the 2D-IR spectra. Changing the sign of the background puts the node on the upper left quadrant cross peak, as shown in **Figure S17**. These errors were corrected in **Figure S13** by adding separate, negative offsets in the lower right and upper left quadrants of the 2D-IR spectrum.

Instead of an added uniform background, **Figure S16(d)** simulates the effect on the polarisation ratio 2D spectrum of the inclusion of a broad diagonal signal which *does not* have a corresponding cross peak, as is caused by the presence of the nest band in the ZSM-5 2D-IR spectra of **Figure 6(b)** main text and the ZSM-5 spectra of **Figure S13**. The added broad diagonal feature causes the cross peak ratios to 'localise', as occurred for **Figure S16(c)**. No nodal lines pass over the cross peaks in either quadrant of the **Figure S16(d)** ratio spectra. The cross peaks farthest from the diagonal are reduced in amplitude to around 0.8, which matches what is observed in the experimental data of **Figure S13**. The polarisation ratio of the near-diagonal cross peaks overlapped with the diagonal band are negative, and reduced in amplitude closer to zero. Increasing the strength of the added broad diagonal simply attenuates all cross peak ratios. Adding a constant negative background (**Figure S16(e)**), we again see the nodal lines appear on the lower right quadrant polarisation ratio cross peaks.

The simulated cross peaks are 'round', showing no correlation between ω_1 pump and ω_3 probe axes. The diagonal peaks are inhomogeneously broadened. Do the polarisation ratio peaks become sloped through the presence of the diagonal signal? In **Figure S16** or **S17** polarisation ratio cross peaks do distort a little. In the experimental polarisation ratio data of **Figure S13**, the proton form ZSM-5 and ferrierite show a clear diagonal slope to the cross peaks. The Na^+ form ZSM-5 water cross peaks do not. This is reasonable evidence for the effect being real.

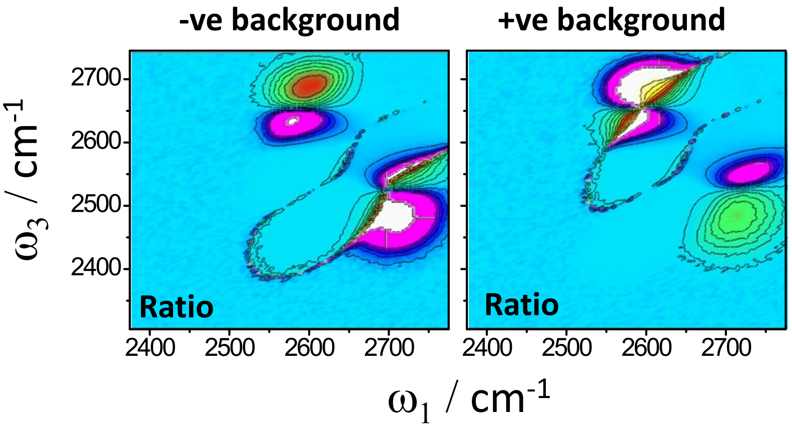

Figure S17 Nodal lines appear on cross peaks in simulated <XXYY>/<XXXX> polarisation ratio spectra when there are backgrounds present in the raw <XXXX> and <XXYY> data. The sign of the background determines which cross peak is affected. In this case, the ratio of the added background in parallel to perpendicular is x3. The effect occurs in the same way for anisotropy plots.

7. 2D-IR signatures of bridged silanols in ZSM-5 samples

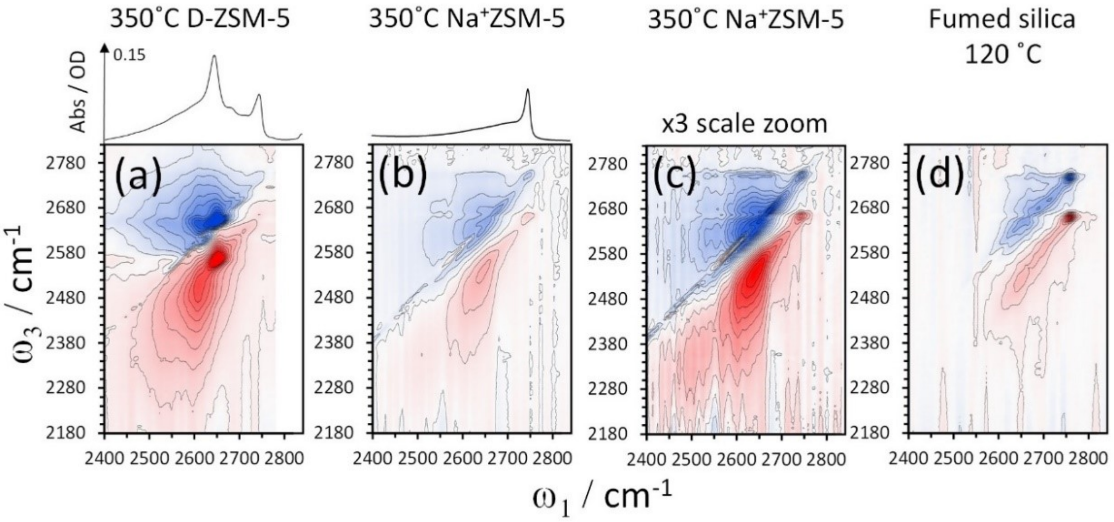

Figure S18. 2D-IR spectra of (a) ‘deuteron-form’ ZSM-5 at 350 ˚C, (b) the same ZSM-5 source as (a), but fully sodium exchanged, deuterated and held at 350 ˚C. (a) and (b) are from pellets of similar thickness and collected during the same set of experiments. The 2D-IR intensity colouring are the same. (c) is a 3x z-scale zoom of (b). (d) is a spectrum of deuterated pyrogenic silica at 120 ˚C. All spectra were collected with perpendicular polarisation (<XXYY>) and a 150 fs waiting time.

Signatures of hydrogen bonded ‘bridged’ silanol groups are observable in ZSM-5 2D-IR spectra, particularly at increased temperatures and / or after Na^+ exchange. They are identifiable by the characteristic cross peak between non-hydrogen bonded and hydrogen bonded bands, as observed previously in pyrogenic silica.[18] **Figure S18(a)** shows 2D-IR and IR absorption spectra of ZSM-5 in ‘deuteron’-form, and prominently displaying the Bronsted ZOD and nest bands. After complete sodium exchange (**Figure S18(b)**), the ZOD band has vanished, and the nest band is significantly attenuated. Evidently sodium disrupts the nests. The IR and 2D-IR spectra of **Figure S18(b)** are plotted on the same scale as **S18(a)** to make clear the loss in IR absorption and 2D-IR signal. The Na^+-ZSM-5 2D-IR spectrum shows the faint hint of a cross peak between the narrow non-hydrogen bonded SiOD bleach on the diagonal and the lower frequency hydrogen bonded SiOD band. Shown at 3x scaling of 2D-IR intensity, (**Figure S18(c)**), the cross peak is very clear. An example of the cross peak in a pyrogenic silica 2D-IR spectrum is shown for comparison in **Figure S18(d)**.

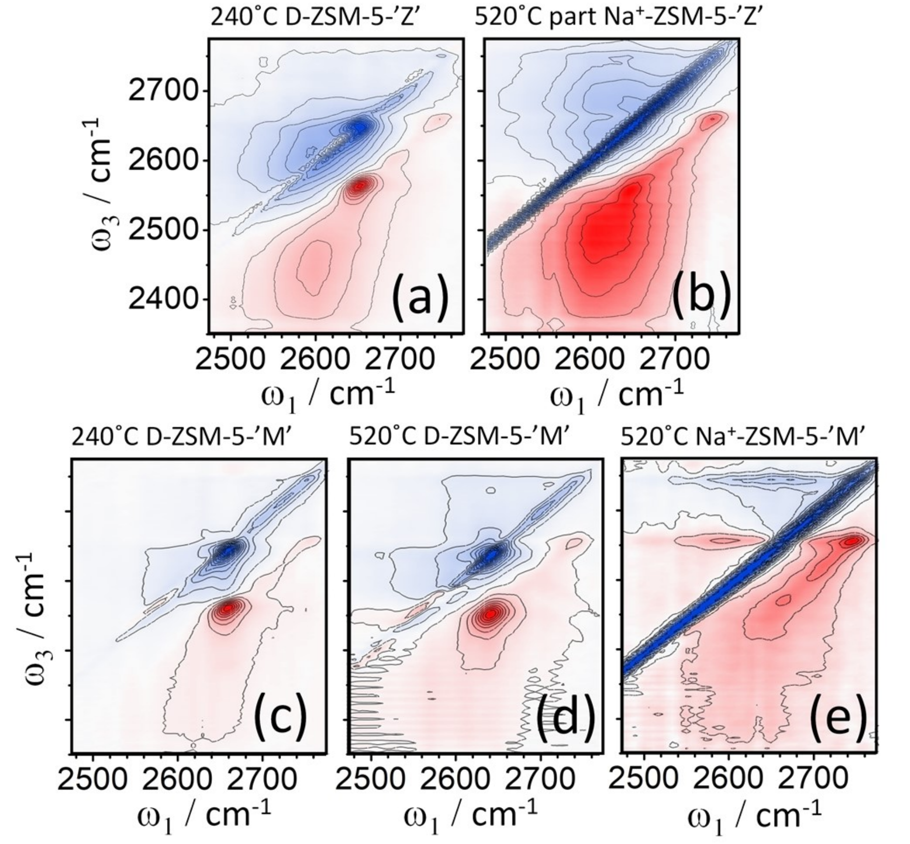

Figure S19. 2D-IR spectra of (a) deuterated Zeolyst ZSM-5 at 240 ˚C, (b) partially exchanged Na^+-form ZSM-5 at 520 ˚C, (c) deuterated ZSM-5 at 240 ˚C from Mobil (d) deuterated ZSM-5 at 520 ˚C from Mobil. (e) deuterated Na^+-ZSM-5 at 520 ˚C from Mobil. All spectra were collected with perpendicular polarisation (<XXYY>) and a ~200 fs waiting time.

Figure S19(a) and **(b)** shows the Zeolyst ZSM-5 sample in 'deuteron' form and partially sodium exchanged. **S19(a)** is for direct comparison with ZSM-5 synthesised by Mobil in the 1980s (labelled 'M'), as described in the materials and methods section of the main text and shown in **S19(c-e)**. Both samples have a Si:Al ratio 24, but the 'M' sample displays very little intrinsic silanol nest absorption. At the highest temperature of 520 °C, **S19(d)**, the bridged silanol cross peak is weakly visible, but dwarfed in size by the ZOD acid site 2D-IR signal. Removal of the Bronsted site by Na^+ exchange, we are left with a 2D-IR spectrum comprising diagonal and cross peak features from bridged silanol groups (**Figure S19(e)**). In the partially sodium exchanged zeolyst 'Z' zeolite sample at 520 °C, **S19(b)**, the nest band is still present, however the diagonal bleaching is wider along the ω_3 axis and the cross peak feature from bridged silanol groups is just-visible on top of this bleaching.

8. Temperature dependence of the silicalite and ZSM-5 nest band

In analysing vibrational spectra of any disordered silaceous material, one has to be aware of sample changes over time, over the course of heat treatment and / or humidification. A proportion of silanol nest bands in silicalite are known to be lost upon heating.[20] The silicalite sample studied in the main text was synthesised and dried under conditions of <120 ˚C. For the 2D-IR measurements, pelleted samples were H-D exchanged at 220-300 ˚C, a temperature at which we observe some irreversible loss of nest band intensity. This is demonstrated by IR absorption spectroscopy as follows. **Figure S20** shows OH-stretch IR absorption spectra taken during a temperature ramp of the silicalite sample (dry N_2 atmosphere) up to 520 ˚C. For this ramp, a substantial amount of nest band absorption is lost by 200 ˚C (>50%). An important issue is how much this is (i) irreversible loss, (ii) reversible loss and (iii) the temperature dependence of the absorption coefficient / bandwidth.

We explored the irreversibility of this process by heating a fresh silicalite sample to 300 ˚C, holding for several minutes and cooling it back down again, and doing the same on a new sample up to 520 ˚C. This is shown in **Figure S21** for a 100 ˚C start and return temperature. The silicalite sample heated to 300 ˚C and cooled back to 100 ˚C irreversibly loses 15% of its nest band intensity (**S21(a)**). Therefore some of the 50% loss of nest band intensity in **Figure S20** up to 300 ˚C is reversible. The silicalite sample heated to 520 ˚C irreversibly loses 50% nest band intensity.

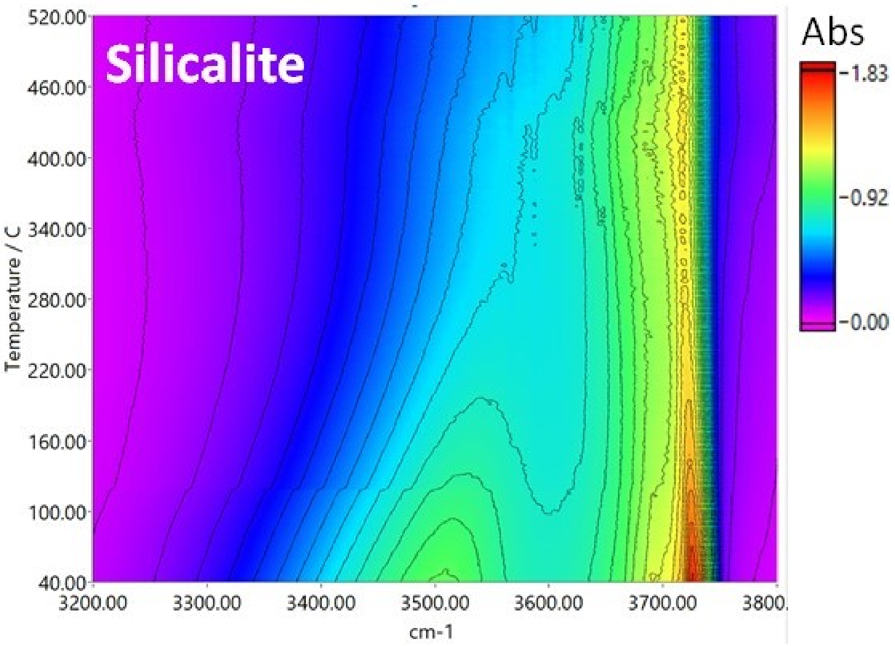

Figure S20. IR absorption spectra of a silicalite pellet in dry N_2 from 40 – 520 C at a rate of 25 ˚C per minute and 10 spectra per minute.

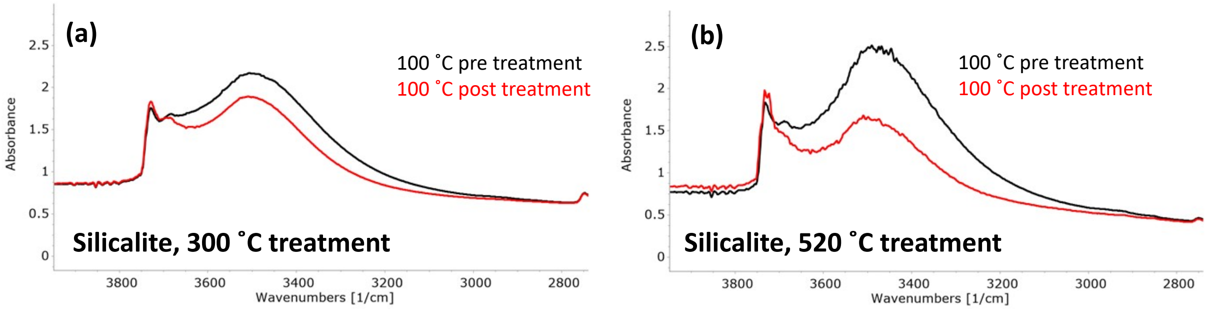

Figure S21. IR absorption spectra of two fresh silicalite pellets at 100 ˚C after (a) several minute treatment at 300 ˚C and (b) several minute treatment at 520 ˚C.

Some of the nests in silicalite are clearly destroyed on heating. Several portions of zeolyst ZSM-5 sample studied (from the same batch) were calcined at 600 ˚C for 12 hours in 2019 and 2020 and stored in vials for use in the experiments reported here (2019-2023). The resulting pelleted samples showed no nest loss on brief temperature cycling. **Figure S22** shows proton form ZSM-5 IR absorption spectra taken during a temperature ramp up to 500 ˚C. The silanol nest band weakens by ~30% from 170 ˚C to 500 ˚C, as does the Bronsted site absorption (**Figure S23**). The free silanol OH stretch at ~3750 cm-1 also shifts and weakens with increasing temperature. In dry N_2 for short periods these changes are reversible. For longer periods at elevated temperature in humid conditions, nest loss occurs. We observed the ZSM-5 nest band to irreversibly weaken by 25% when held for 12 hrs in an atmosphere of D_2O humidified N_2 vapor for a slow temperature ramp to 520 ˚C. This is shown in **Figure**

S24. Here there is also a 10% loss of Bronsted ZOD absorption caused by ‘steaming’ (water induced dealumination of the zeolite, a process which actually generates nests).

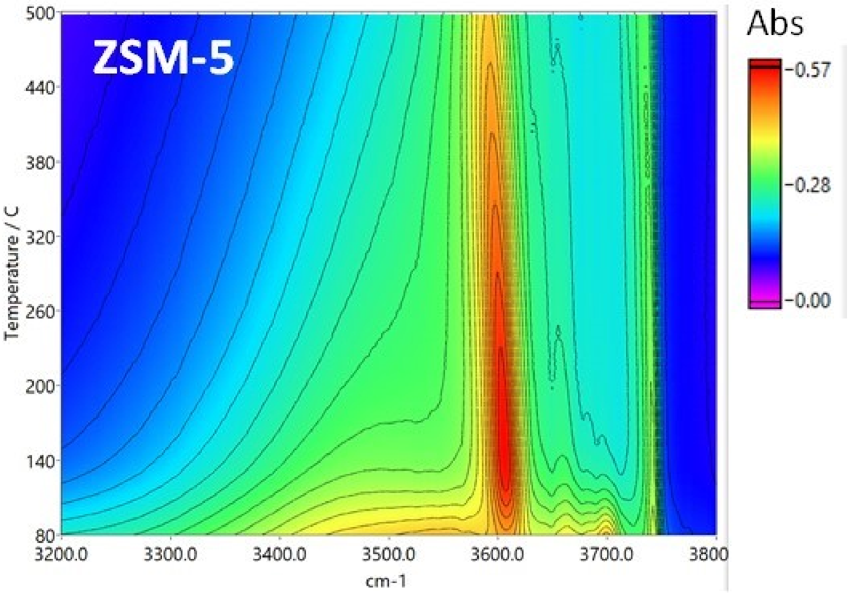

Figure S22. IR spectra of a fresh ZSM-5 pellet in dry N_2 heated from 40 – 500 ˚C at a rate of 5 ˚C per minute and 2.5 spectra per minute.

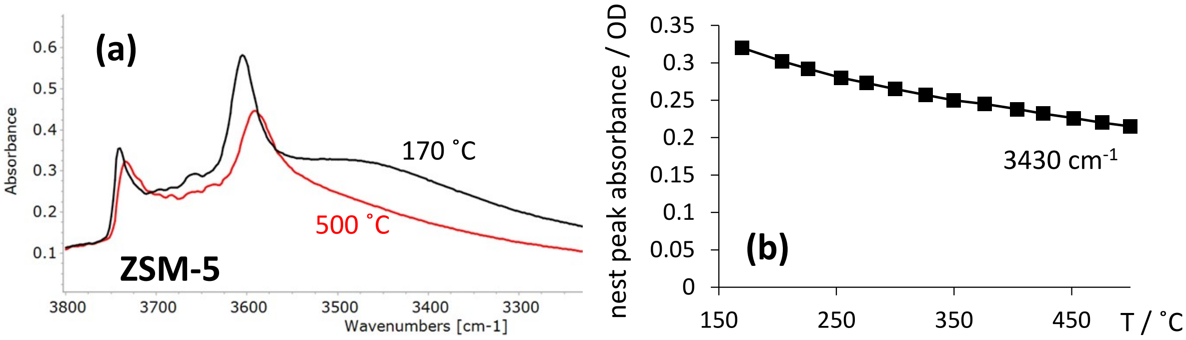

Figure S23. (a) IR absorption spectra of a fresh ZSM-5 pellet in dry N_2 at 170 ˚C and 500 ˚C. (b) Absorbance of the nest band at peak (3430 cm^{-1}) as a function of temperature. The changes observed in (a) and (b) were reversible.

After the 12 hour temperature ramp described by **Figure S24**, the zeolite samples were studied at five different temperatures over the shorter total time of two hours. 2D-IR spectra collected are shown in **Figure S25**. Corresponding IR absorption spectra are shown in **Figure S26**. Apart from a small amount of nest absorption loss between 240 and 310 ˚C, the nest band does not further reduce noticeably in the FT-IR data, though the 2D-IR peak signal drops by a further 45% up to 520 ˚C. This is possibly an effect of the bandwidth and transition strength changing slightly.

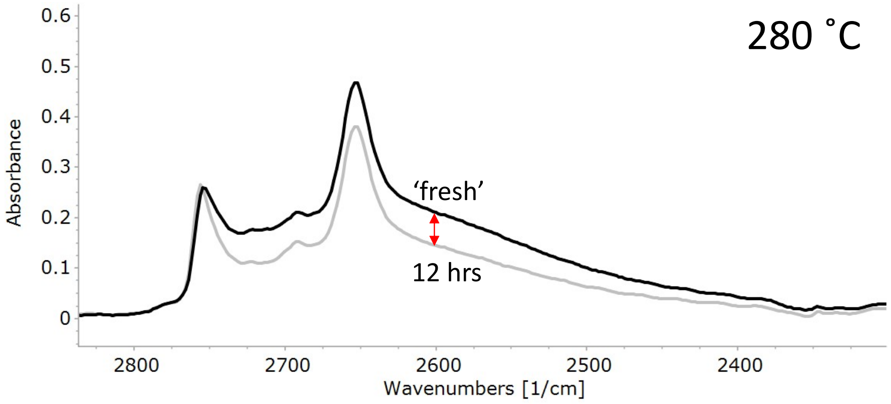

Figure S24. IR absorption spectra of a fresh ZSM-5 pellet sandwiched between CaF_2 in D_2O saturated N_2 at 280 ˚C. The 'fresh' sample was taken through a 12 hour temperature ramp from 100 ˚C to 520 ˚C.

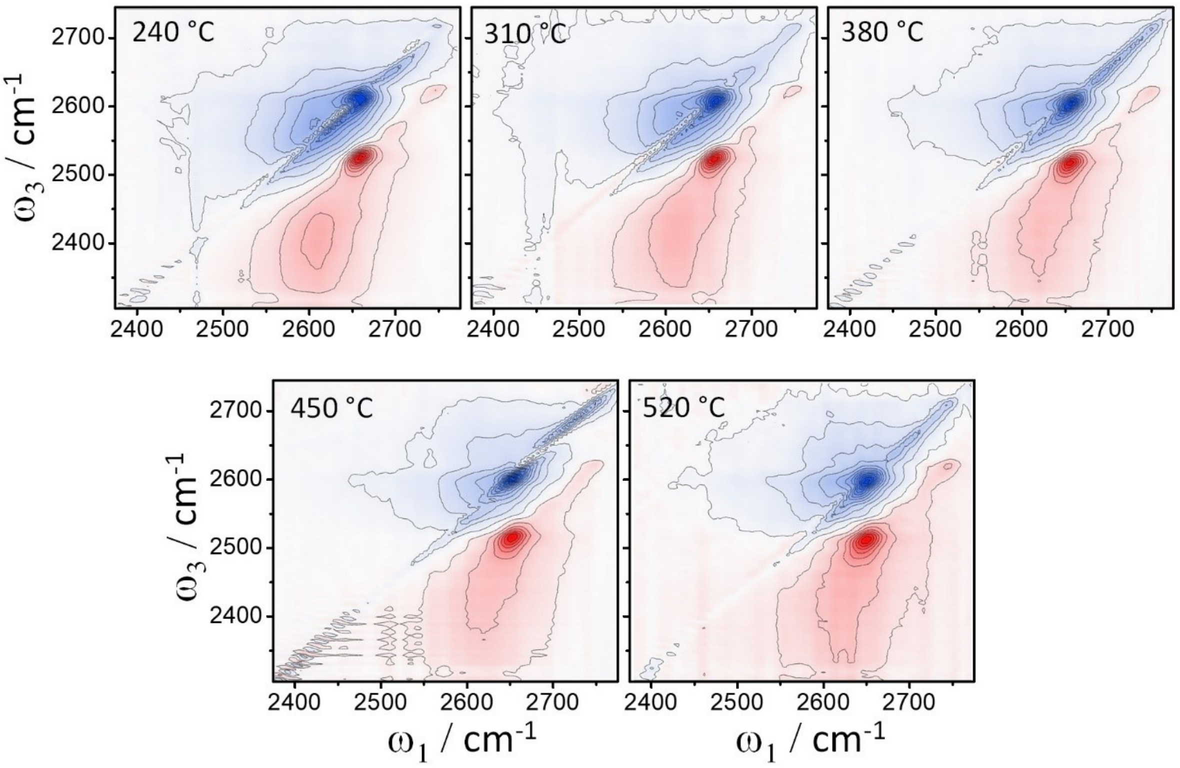

Figure S25. Temperature dependent 2D-IR spectra of deuterated pellets of ZSM-5 held tightly between CaF2 windows in an atmosphere of D_2O in flowing N_2 on a 12 hr temperature ramp to 520 ˚C prior to measurement. Crossed polarisation <XXYY> and a waiting time of t_2 = 300 fs were used. These spectra have had their corresponding negative waiting time (t2 = -100 ps) subtracted. Each spectrum was collected 20 minutes apart.

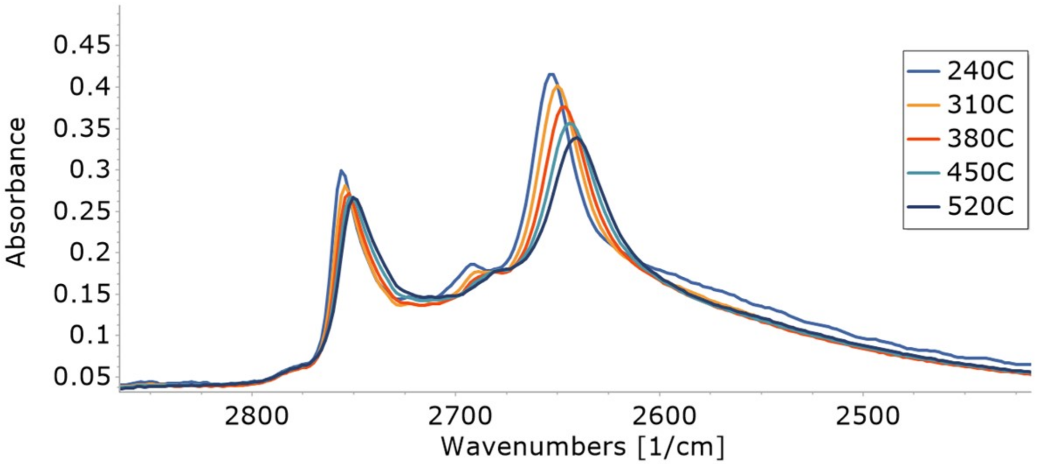

Figure S26. Temperature dependent FT-IR spectra corresponding to each sample measured by 2D-IR in **Figure S25**

9. ZSM-5 and silicalite 2D-IR spectra as a function of temperature and waiting time

2D-IR spectra of ZSM-5 and silicalite collected on a single experimental run are shown in **Figures S27-29**. Thus, all laser and spectrometer parameters were near-identical. It was not possible to cool the ZSM-5 below 50 ˚C without N_2 adsorbing onto the Bronsted sites. From these datasets, in combination with the parallel measurements (not shown), the anisotropic and isotropic signals in **ESI Section 13** and **14** were calculated.

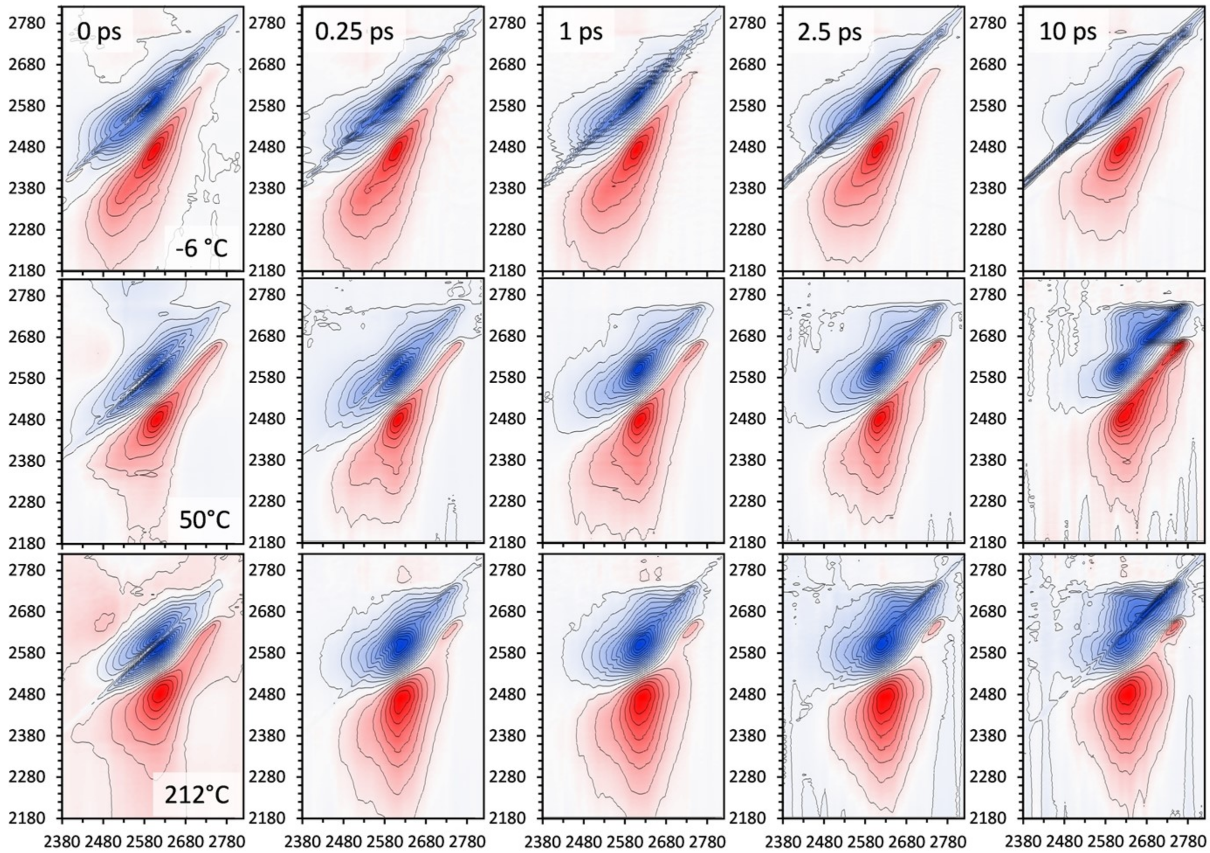

Figure S27. 2D-IR spectra of silicalite kept dry after H-D exchange at ~250 °C by holding pellet tightly between CaF_2 windows in an atmosphere of dry N_2. From left to right is population time. From top to bottom is temperature. The 2D-IR spectra are <XXYY> and plotted with ω_1 on the x axis and ω_3 on the y axis, both in cm^{-1}.

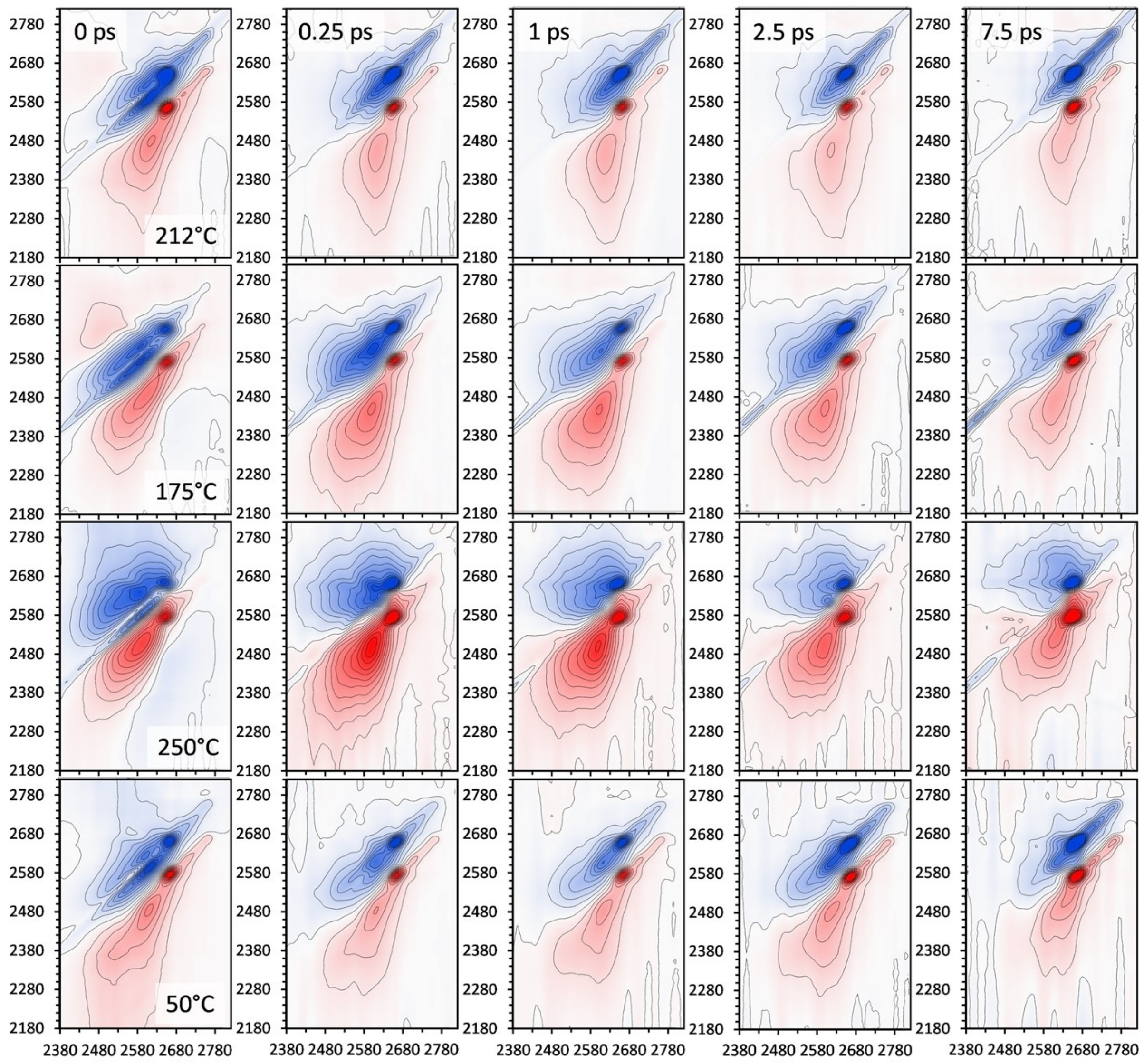

Figure S28. 2D-IR spectra of ZSM-5 in held in a vapour stream of D_2O diluted in N_2. The 50 °C sample was kept dry after exchange and dehydration by holding pellet tightly between CaF_2 windows. From left to right is population time. From top to bottom is temperature. The 2D-IR spectra are <XXYY> and plotted with ω_1 on the x axis and ω_3 on the y axis, in units of cm^{-1}. The z-scale has been limited in order to reveal the weaker features.

Figures S27 and **28** were recorded under dry conditions. The spectra in **Figure 29** were recorded in a wet vapour stream. When recorded in wet vapour streams, clean subtractions of silicalite from ZSM-5 2D-IR spectra were not possible, regardless of the scaling used.

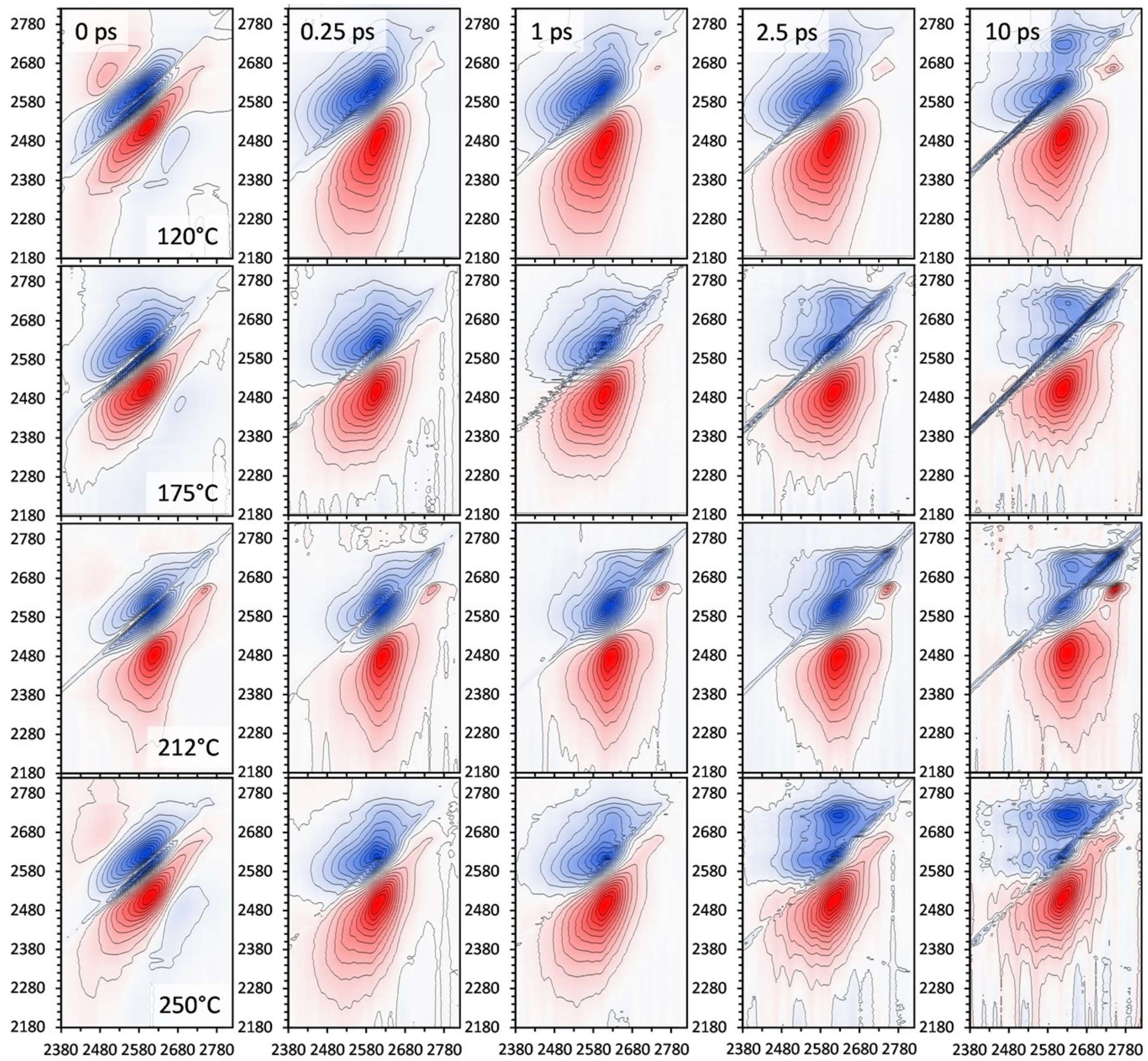

Figure S29. 2D-IR spectra of silicalite held in a vapour stream of D_2O diluted in N_2. After exchange at ~220 °C. From left to right is population time. From top to bottom is temperature. The 2D-IR spectra are <XXYY> and plotted with ω_1 on the x axis and ω_3 on the y axis, both in cm^{-1}.

10. Further difference spectra of ZSM-5 and silicalite

The lowest possible temperature to cool dehydrated ZSM-5 to without adsorption of nitrogen at the Bronsted sites was ~50 ˚C. Difference spectra at these temperatures are shown in **Figure S30** for waiting times of t_2 = 0.1 ps and 1 ps. These extra data reinforce three important points. (1) That the differencing of the spectra is reliable at early waiting times, (2) that the cross peak observed in the main text does not feature so prominently at either waiting time at this temperature and (3), that the 2D-IR spectra of ZSM-5 and silicalite clearly show two nest bands.

As described in the main text, the cross peak between the nest band and higher frequency modes (ω_3~2710 cm^{-1}) of ZSM-5 and silicalite grows as a function of waiting time. The peak is observed clearly in ZSM-5 in **Figure S31(a)** for the long waiting time of 10 ps. The cross peak currently escapes exact explanation. Possible origins are a dynamic structural exchange effect or a heating signal ('hot-ground-state'). It is not evident at earliest waiting times or in the lower temperature silicalite 2D-IR spectra (see **ESI section 9**). It is perfectly subtracted in the scaled difference spectrum of **S31(c)**, reinforcing

that the peak is common to both ZSM-5 and silicalite samples. The remaining positive absorption in **S31(c)** is a different feature from ZSM-5 and visible in **S31(a)**.

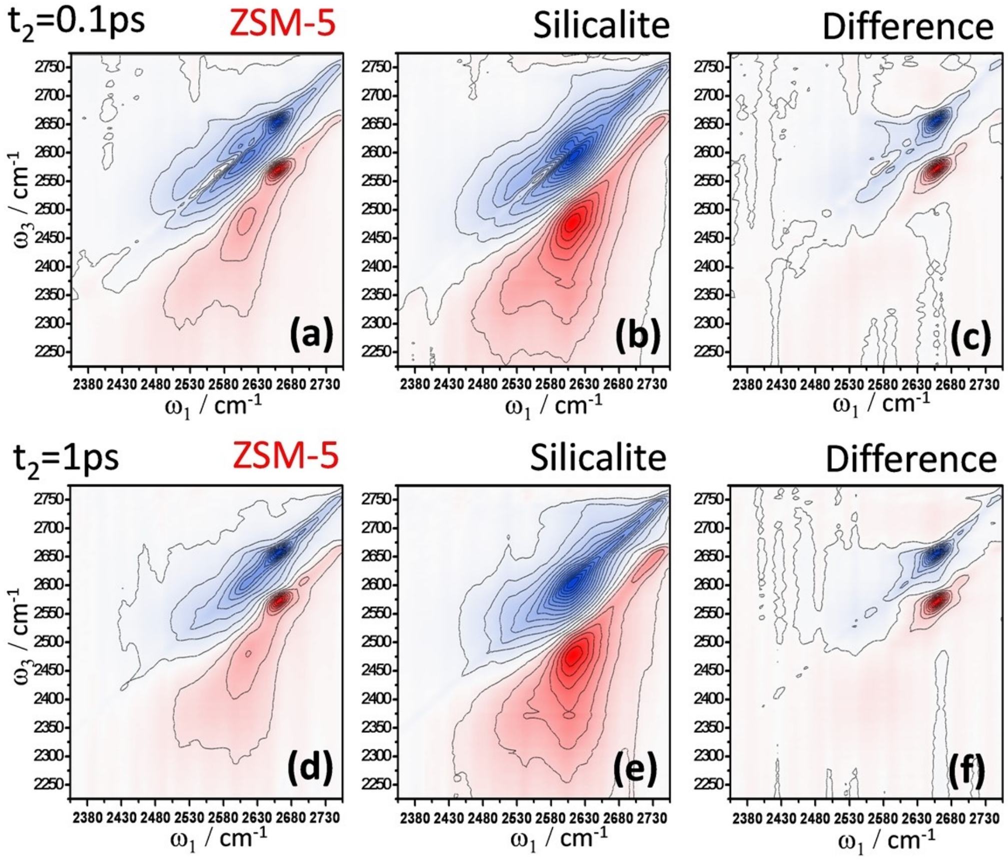

Figure S30. 2D-IR spectra of deuterated pellets of ZSM-5 (a,d) and silicalite (b,e) held at ~50 ˚C between CaF_2 plates in an atmosphere of dry N_2. Crossed polarisation <XXYY> and a waiting time of t_2 = 0.1 and 1 ps were used as indicated. Differences of the zeolite and silicalite 2D-IR are shown in (c) and (f). For these samples, a scale factor applied to the silicalite spectrum of ~0.45 best subtracted the broad feature in the ZSM-5 2D-IR spectrum.

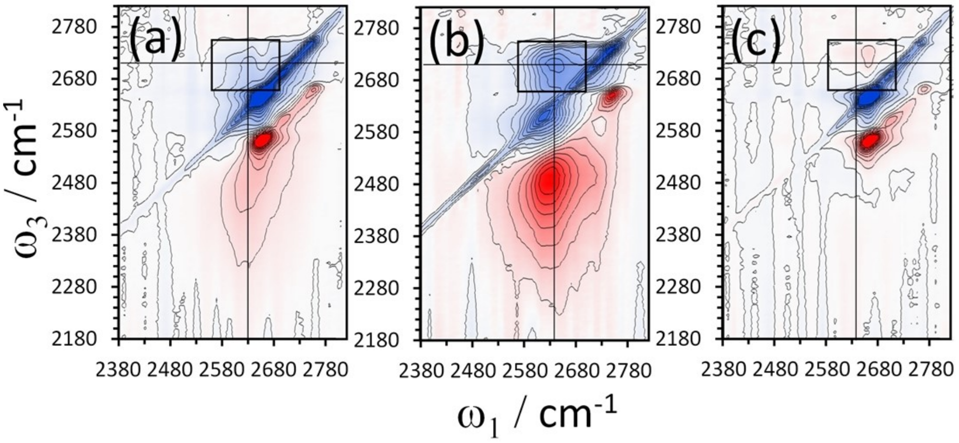

Figure S31. 2D-IR spectra of deuterated pellets of ZSM-5 (a) and silicalite (b) held at ~212 °C an atmosphere of flowing N_2 / D_2O. Crossed polarisation <XXYY> and a waiting time of t_2 = 10 ps was used. Differences between the zeolite and silicalite 2D-IR spectra are shown in (c). The data are part of the same waiting time measurement series as Figure 3 in the main text. A scale factor applied to the silicalite spectrum of ~0.5 best subtracts the broad feature in the ZSM-5 spectrum. The z-scale of the ZSM-5 spectrum and the difference spectrum has been limited in order to reveal the weaker features.

11. The decays of 2D-IR amplitude (vibrational lifetimes) reveal two silanol nest features

For 2D-IR spectral amplitude fitting as a function of waiting time t_2 in the main text, the simple and crude approach of single exponential fits was invoked, despite the 2D-IR data potentially comprising overlapping bands and backgrounds, each potentially with multiple relaxation timescales. In **Figure S32(a)**, examples of experimental 2D-IR amplitude vs waiting time (t_2) data for the 120 °C ZSM-5 measurement described in **Figure 4** of the main text are shown, alongside the exponential fits to the data. The fits give amplitudes systematically too small at long times, with fit errors of 10-15%. When this same process is run through the isotropic spectrum calculated from <XXYY> and <XXXX> spectra (raw data shown in **ESI section 12**), or applied to the ESA band, we obtain very similar results (**Figure S32 (c)**). As we will show shortly, the decays are bi-exponential. Though it is obviously possible to go through the whole fitting process using bi-exponential decays, we instead continue using the simple single exponential fit simply as a tool to reveal the origins of the frequency dependent lifetimes.

Although by appearance, at raised temperature, the nest band appears to be just a single band, at temperatures of ~ < 75 ˚C, 2D-IR spectra of silicalite and ZSM-5 suggests there are in fact two bands: one at ~2550 cm^{-1} and one at ~2600 cm^{-1} (see **ESI section 10**). In all data examined, despite the coalescence of the two bands at higher temperatures, the lower frequency band relaxes faster than the higher frequency band. This being the case, we make a simple model to rationalise how this manifests in the simplified single exponential 2D-IR lifetime analysis.

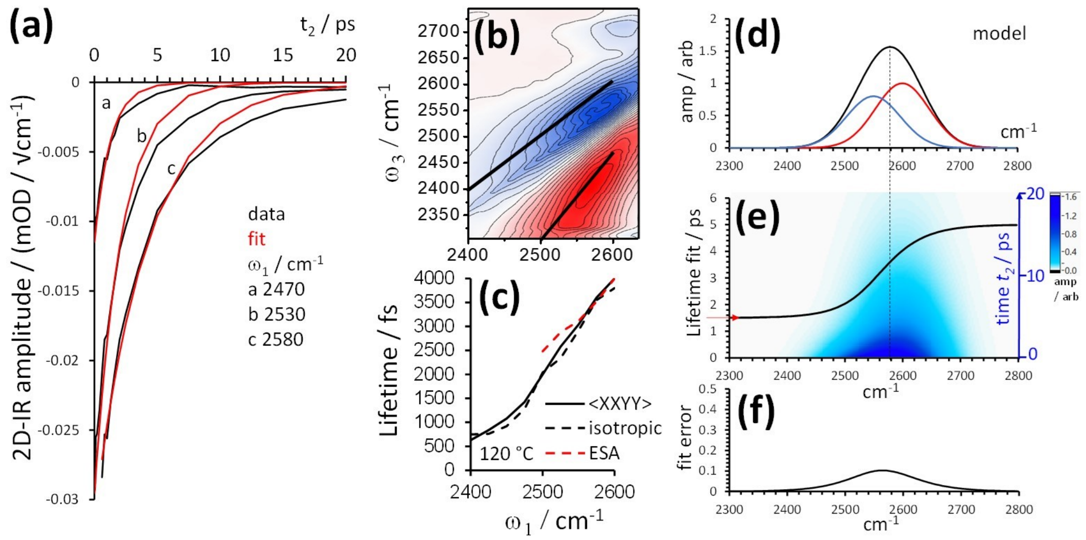

Figure S32. (a) Examples of 2D-IR signal amplitude data and exponential fits as a function of waiting time t_2 and ω_1 for the 120 °C ZSM-5 2D-IR spectrum discussed in the main text. The 2D-IR spectrum in (b) is the 50 fs, 120 °C ZSM-5 spectrum from Figure 4(a) in the main text, but includes a line on the ESA feature showing where exponential fitting was also conducted. The lifetimes from the fits of the ESA, the isotropic signal calculated from polarisation dependent data and the original <XXYY> amplitude data are shown together in (c). A simple two-band, two-lifetimes model (d) with a band at 2550 cm^{-1} (lifetime 1.5 ps) and a band at 2600 cm-1 (lifetime 5 ps) gives the amplitude vs time vs cm^{-1} plot shown in (e). The fitted exponential lifetime is superposed (black line), showing the same behaviour as the experimental data. In (f), the fit error (95% confidence) for single exponential fitting of this model is plotted.

Figure S32(d) shows a simple model IR spectrum comprising two Gaussians of 110 cm^{-1} FWHM. With only 50 cm^{-1} separation of the two bands, at this width it is not possible to see by eye that there are two bands at all, and it is doubtful that any fit of an experimental IR absorption spectrum could be confidently used to assign two bands for such a spectrum. This spectrum resembles the IR absorption spectrum of the nest band at T > 75 °C and the diagonal 2D-IR bleach signal. The two bands were

assigned lifetimes of 1.5 and 5 ps. The simulated signal amplitude versus time versus frequency (cm^{-1}) is plotted in **Figure S32(e)**. Lifetimes from single exponential fits to the simulated data as a function of frequency (cm^{-1}) are overlaid in **Figure S32(e)**. Very similar behaviour to the experimental data of **Figure S32(c)** and **Figure 4(b)** (main text) is observed: the fitted lifetime varies rapidly across the centre of the band. The 2D-IR spectral amplitude is obviously varying bi-exponentially in this region of spectral overlap. **Figure S32(e)**) shows that the error of the single exponential fit is largest (10%) in this region. This error is comparable to that observed in the experimental data, however we may often see a 10% error on single exponential fit lifetimes from instrument response effects, growth of hot ground-state bands, backgrounds and experimental noise.

12. Parallel and perpendicular polarisation ZSM-5 2D-IR measurements used to calculate the anisotropy values of Figure 4(d)

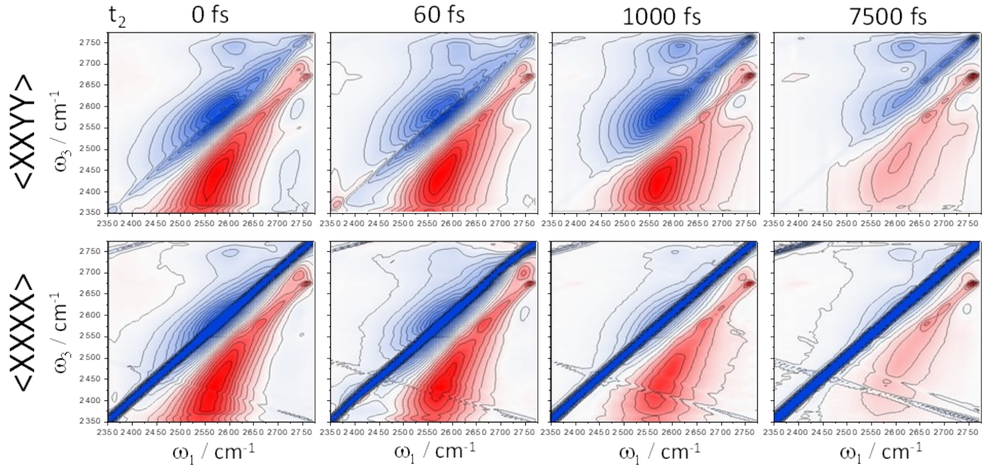

Figure S33. Some of the perpendicular (<XXYY>) and parallel (<XXXX>) components of the measurements from Figure 6(b) and Figure 4(a) (main text), and Figure S6 (ZSM-5 sample in a flow of N_2 / D_2O vapour at ~120 °C). This data was used to calculate anisotropies in the main text Figure 4(d).

13. Silicalite vs ZSM-5, anisotropy decays and lifetimes

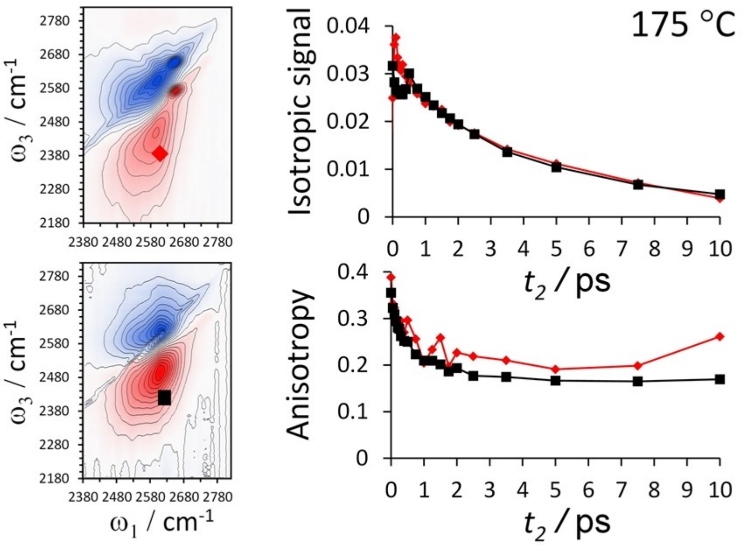

Figure S34. Isotropic and anisotropic signal derived from ZSM-5 (red) and Silicalite (black) 2D-IR spectra from points indicated by the marker shapes in the 2D-IR spectra. The Silicalite isotropic signal has been scaled by ~3x to give the match with ZSM-5. The deuterated pellets of ZSM-5 (a) and silicalite (b) held at ~175 ˚C in an atmosphere of D_2O diluted in N_2. The 2D-IR spectra shown are crossed polarisation <XXYY>.

In this section, we demonstrate that the isotropic and anisotropic 2D-IR signal decay behaviour of ZSM-5 and silicalite nest bands are identical. A representative example is shown in **Figure S34** for measurements performed 'back-to-back' during the same measurement session at 175 ˚C. The points on the 2D-IR spectrum at which the anisotropy and isotropic signal are calculated are shown in the 2D-IR spectra on the left. The isotropic signal decay from silicalite has been scaled down by x3. The match between ZSM-5 and silicalite is perfect for the isotropic signal, and good for the anisotropy (though there is clearly more noise in the ZSM-5 plot). This occurs when small amounts of heterodyne scatter is present in the parallel measurement contributing to the anisotropy calculation. Averaging over 5-6 multiple points of the 2D-IR spectrum spaced 16 cm^{-1} around the central point can reduce this and was used in the main text to give the best averages.

Figure S35 shows another example of isotropic signal and anisotropic signal decay plotted from two different points of the diagonal ESA band spaced ~80 cm^{-1} apart for ZSM-5 and silicalite samples at 50 ˚C. The isotropic signal decay from silicalite has been scaled down by x3 and again matches perfectly that of ZSM-5 at both marked positions on the ESA. The ZSM-5 anisotropy decay has more noise from heterodyne scatter, but the ZSM-5 and silicalite samples nevertheless follow similar trends at the two different probe wavelengths. Moving away from the diagonal, the anisotropy decay speeds up / decays to a slightly lower offset.

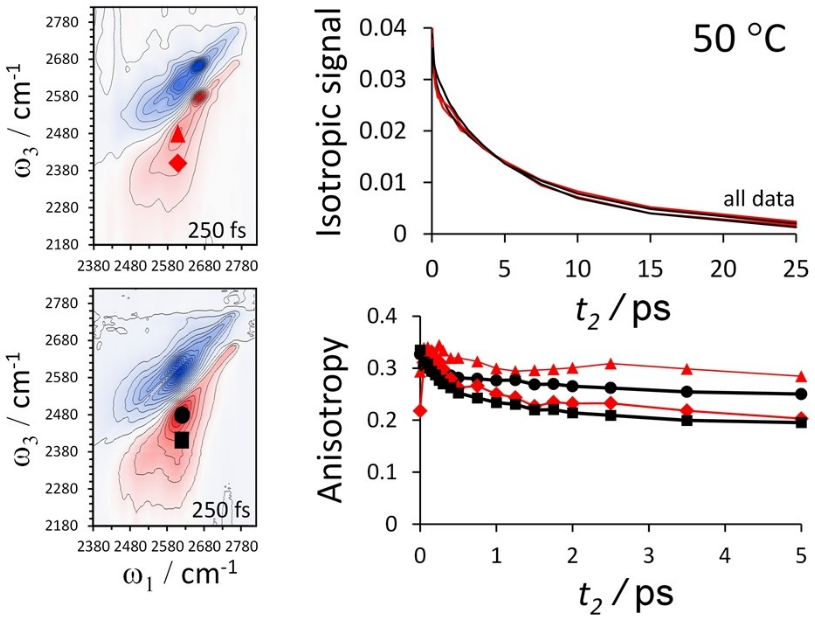

Figure S35. Isotropic and anisotropic signal derived from ZSM-5 (red) and Silicalite (black) 2D-IR spectra at points indicated by the marker shapes in the 2D-IR spectra. The Silicalite isotropic signal has been scaled by 3x to give the match with ZSM-5. The deuterated pellets of ZSM-5 (a) and silicalite (b) both held at ~50 ˚C between CaF2 plates in an atmosphere of dry N2. The 2D-IR spectra shown are crossed polarisation <XXYY>.

In the main text, the silicalite anisotropy decays of **Figure 5** were truncated at 8 ps for comparison with the nodal slope decays. The full range is reproduced below in **Figure S36**.

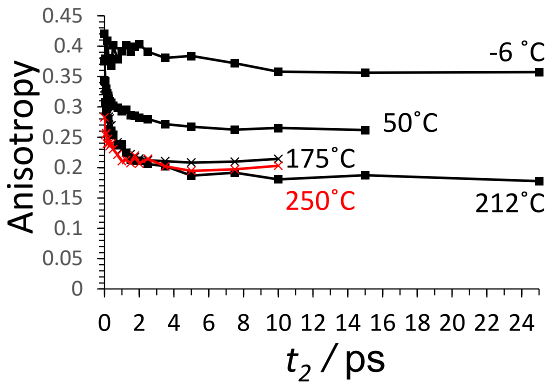

Figure S36. Temperature dependence of the anisotropic signal from Figure 5 main text plotted across the full experimentally accessible range.

14. The lower frequency silanol nest band has a faster anisotropy decay than the ~2600 cm^{-1} silanol nest band

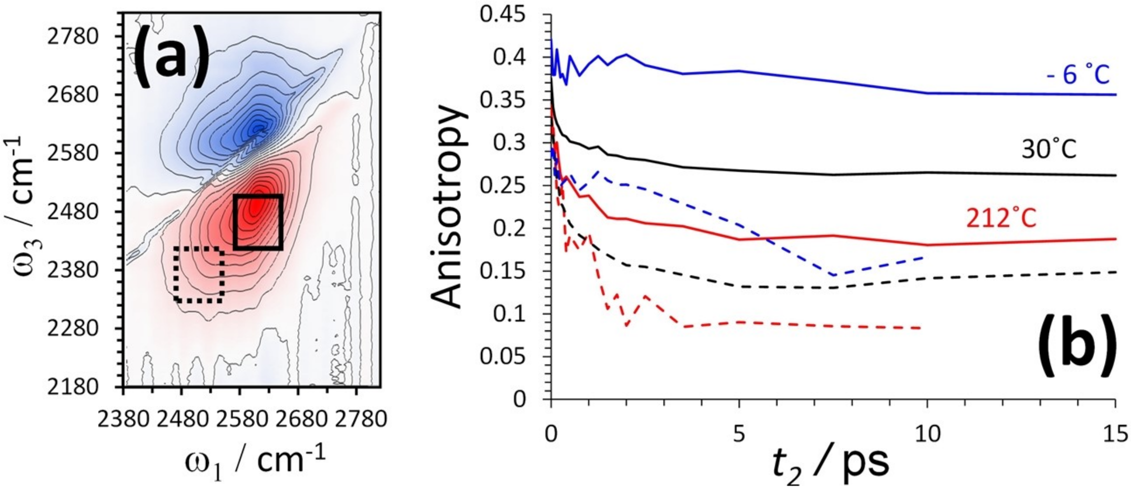

Figure S37. Temperature dependence of the anisotropic signal decays of the low and high frequency nest bands of silicalite averaged over points within the boxes as indicated in the 2D-IR spectrum (a). In the anisotropy decay plots (b), the high frequency band anisotropy is shown with solid lines. The low frequency box anisotropy decay is shown with dashed lines. The three sample temperatures follow the trend of slower to faster for cooler to hotter (blue (-6C), black (+30C), red (212C)).

15. Silanol nest / Bronsted site concentration ratio calculations

The concentration ratio equation is:[21]

$$\frac{[c_a]}{[c_b]} = \frac{A_a^2}{A_b^2} \cdot \frac{S_b}{S_a} \cdot \frac{F_a}{F_b}$$

Eq. S4

In order to arrive at an estimate of the ratio of nest deuteroxyl groups to non-hydrogen bonded Bronsted (ZOD) sites in the ZSM-5 samples studied, we need to determine the following from the same sample under the same conditions.

(1) A_{ZOD}, the peak IR absorbance of the ZOD Bronsted stretch band

(2) A_{nest}, the peak IR absorbance of the nest band

(3) S_{ZOD}, the peak 2D-IR diagonal bleach signal from the ZOD Bronsted stretch band

(4) S_{nest}, the peak 2D-IR diagonal bleach signal from the nest band

(5) F_{ZOD} and F_{nest} : the 2D-IR correction factors[21]

The factors reducing the IR absorbance measurement accuracy are spectral congestion and the uncertainties between intrinsic sample absorption and scattering. The challenge in determining 2D-IR diagonal bleach signals are the required bleach signal overlapping with diagonal scatter and thermal artefact signal. Uncertainties in the lineshape affect the estimation of the correction factor. It is advantageous to use the diagonal ESA bands of the 2D-IR spectrum as a measure the desired bleach signal strength, as the ESA is always free-from scatter and thermal transient signal. Studying a number of ZSM-5 2D-IR measurements recorded in the temperature range of 150 ˚C – 300 ˚C as a function of t_2 with varying amounts of scatter and thermal artefact across the bleach diagonal, we observe the peak diagonal ground state-bleach strength and Excited State Absorption (ESA) strength to be similar for both the nest band and the ZOD stretch around 0 – 200 fs waiting time.

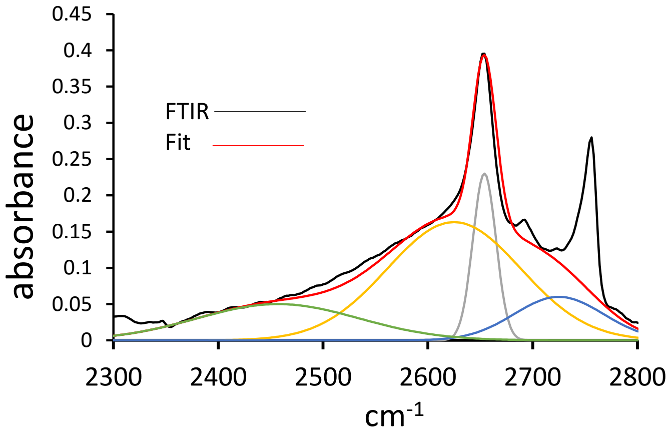

Figure S38. 4-peak Gaussian fits of the IR spectra are used for gaining a measure the absorption of a sample's nest band at ~2600-2620 cm^{-1}. This IR spectrum is for measurement (A) in Table S2 (240 °C).

For the IR absorption measurements, Gaussian fitting is used as means of modelling the IR spectrum, as shown in **Figure S38**. The 4-peak fit superficially captures the form of the spectrum but should not be considered a proper decomposition of the spectrum, which evidently contains more bands. The fit is simply to estimate the peak band absorption sizes. As the fitting is somewhat approximate and the baseline of the ZSM-5 absorption uncertain, owing to the square of A_{sample} in **Eq. S4** the resulting uncertainty affects the final uncertainty more than the uncertainty in the 2D-IR signal measurement. The 2D-IR spectrum can provide useful constraints by defining the centre wavelength and width of the nest band. On the present setup, this was limited by pump bandwidth, pump calibration uncertainties, temperature uncertainties and the need to make IR and 2D-IR measurements on separate instruments.

For 2D-IR signal measurements, values are read-out from cuts as shown in **Figure S39**. The ZOD stretch IR absorption and 2D-IR signals overlap with the nest band, with the latter therefore subtracted in the measurement.

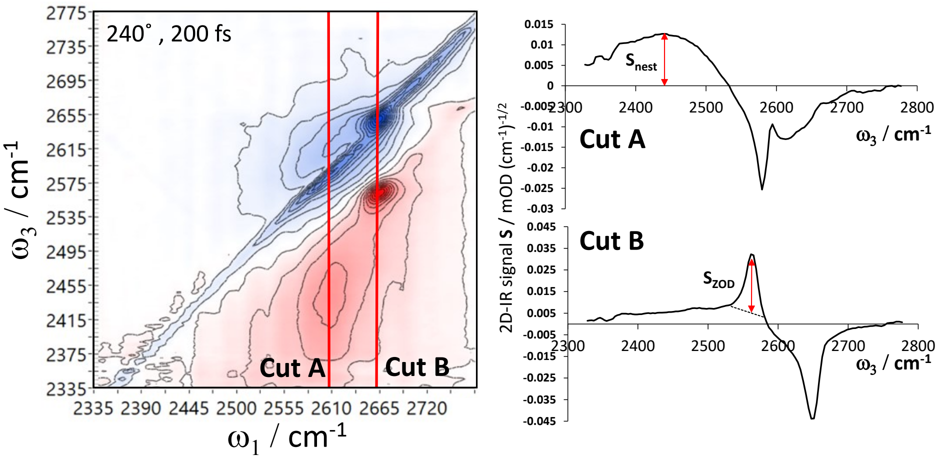

Figure S39. Cuts of 2D-IR spectra at t_2 = 200 fs are used to determine the diagonal 1->2 ESA signal strengths as estimates of the bleach strength required for the concentration ratio calculation. Shown here is the 2D-IR spectrum giving the signals for measurement (A) in Table S2. This spectrum was acquired with 100s signal averaging and collected using crossed polarisation <XXYY>.

	A_{nest}±0.025	A_{ZOD}±0.025	S_{nest}±0.001	S_{ZOD}±0.003	F_{nest}	F_{ZOD}	C_{nest}/C_{ZOD}
**A.** 240 °C	0.16	0.23	0.012	0.027	0.43	0.43	1.1 ± 0.3
**B.** 310 °C	0.15	0.23	0.01	0.026	0.43	0.43	1.1 ± 0.3
**C.** 380 °C	0.14	0.2	0.007	0.019	0.44	0.46	1.4 ± 0.4
**D.** 450 °C	0.14	0.19	0.005	0.016	0.45	0.47	1.8 ± 0.5
**E.** 520 °C	0.14	0.18	0.003	0.006	0.45	0.47	1.3 ± 0.8
**F.** 175 °C	0.3	0.34	0.012	0.019	0.38	0.35	1.1 ± 0.3
**G.** 212 °C	0.28	0.33	0.015	0.019	0.39	0.35	0.8 ± 0.3
**H.** 250 °C	0.28	0.33	0.009	0.024	0.39	0.35	1.7 ± 0.3

Table S2. A concentration ratio calculation for the Zeolyst ZSM-5 sample (Si to Al ratio of 24) at different temperatures. Listed are values of the ZOD and nest peak absorbance, peak bleach-SE 2D-IR signals (mOD / $(cm^{-1})^{1/2}$) and correction factors *F* used to calculate the concentration ratio $C_{ZOD…Z}/C_{ZOD}$ at two temperatures. The uncertainties in *A*, *S* and *F* values are used to calculate the uncertainty in the concentration ratio. The uncertainties in *F* values are described in Table S3. Uncertainties in IR absorption are due to the uncertainty in the zero of absorption due to scattering. Uncertainties in the 2D-IR signal measurement arise from a nonzero instrument response, thermal artefact and scattering backgrounds. An additional uncertainty in S_{ZOD} arises from a fast signal drop at around time-zero that is hard to quantify. (A)-(B) are the same sample measured consecutively from 2D-IR acquisitions of 100s averaging time, each with an FT-IR spectrum measured directly after. (F)-(H) are fresh pellet samples each time.

The *A*, *S* and *F* values estimated for two ZSM-5 samples at multiple temperatures are shown in **Table S2** along with the calculated nest hydroxyl to Bronsted hydroxyl concentration ratios. Further discussion of how the *F* values were arrived at follow in the next paragraphs. First, we remark that the concentration ratios are reasonably consistent with one another. The ratio of calculated *F* factors are also ~1. The samples were all exposed to N_2 / D_2O vapor at high temperature for deuteration and other investigations in the 2D-IR setup prior to execution of the measurements for **Table S2**. Both the nest and Bronsted total amounts will therefore have degraded by some tens of percent relative to the freshly calcined sample (see **Figure S24** in **ESI section 8**).

The measurements point to the ratio of nest OD groups and Bronsted OD groups being approximately equal in number. We are more confident of these numbers at < 300 ˚C temperatures. At the higher temperatures, the nest and Bronsted bands broaden. With the quality of the available data it becomes difficult to estimate the diagonal and anti-diagonal widths of the two bands in the 2D-IR spectra. Although it is clear the lineshapes slightly change with temperature, in **Tables S2** and **S3**, we assume

that the bands remain inhomogeneously broadened by a factor 2, but reduce the confidence in the measure by increasing the uncertainty.

	$\Delta\omega_{nest}^{hom}$	$\Delta\omega_{ZOD}^{hom}$	$\Delta\omega_{nest}^{inhom}$	$\Delta\omega_{ZOD}^{inhom}$	B_{nest}	B_{ZOD}	η_{nest}	η_{ZOD}
**A.** 240 °C	80	20	150	30	0.53±0.05	0.67±0.05	0.81	0.64
**B.** 310 °C	"	"	"	"	" ±0.05	" ±0.05	0.83	0.65
**C.** 380 °C	"	"	"	"	" ±0.07	" ±0.07	0.83	0.7
**D.** 450 °C	"	"	"	"	" ±0.1	" ±0.1	0.83	0.7
**E.** 520 °C	"	"	"	"	" ±0.15	" ±0.15	0.85	0.7
**F.** 175 °C	"	"	"	"	" ±0.05	" ±0.05	0.72	0.52
**G.** 212 °C	"	"	"	"	" ±0.05	" ±0.05	0.73	0.53
**H.** 250 °C	"	"	"	"	" ±0.05	" ±0.05	0.73	0.53

Table S3. Calculation of inhomogeneous broadening correction factors *B* and 2D-IR signal attenuation factors η (caused by linear absorption variations between the nest and ZOD bands). The B values are derived from estimates of width $\Delta\omega$ in cm^{-1} deduced from the 2D-IR spectra. The η accounting for pump laser attenuation across the sample are calculated from the IR absorption spectra as described previously.[21]

Correction factors contributing to *F* values

Laser spectral intensity variation: There is no need to correct for laser intensity variations. The peak of the nest band and the ZOD band are within 50 cm^{-1} of one another. With a laser intensity FWHH of 250 cm^{-1} the quantities[21] $I_{ZOD..Z}$ ~ 1 and I_{ZOD} ~ 1.

Relaxation factor: As mentioned above, we observe the nest band signal strength to remain constant between 0 fs and 200 fs. The ZOD band exhibits a 10-20% drop. We cannot confidently distinguish between instrument response and relaxation effects, however the former seems more likely for this >50 ps lifetime band, so we take the 200 fs value. The increased uncertainty in the measurement is reflected in the error used.

Inhomogeneous broadening: The full-width at half-height (FWHH) values of the ZOD and nest band along the diagonal and anti-diagonal are readily estimated simply by counting equal-spaced contours of a 2D-IR spectrum. It is observed that at t_2 = 0, both ZOD and nest bands are inhomogeneously broadened by a factor of ~2. Although the nest band shows rapid spectral diffusion, the ZOD band remains inhomogeneously broadened out to long (>10 ps) times on both the bleach/SE and ESA. As mentioned above, our confidence in this measurement degrades at higher temperatures, so we increase the uncertainty in the broadening factors '*B*' used in the calculation of *F.*

Anharmonicity factor: There is no need to correct for small anharmonicity and subsequent bleach-ESA overlap affecting signal size. The homogeneous linewidth of the ZOD and nest bands are 30 and 80 cm^{-1}. The bleach/SE and ESA peak separations are ~70 cm^{-1} and ~80 cm-1 respectively. Under these circumstances, the ZOD bleach/SE and ESA bands are not attenuating one another and the nest attenuation will be minimal, therefore the corrections due to anharmonicity are $\chi_{ZOD..Z}$ ~ 1, χ_{ZOD} ~ 1.

Absorption of pump light by sample: The corrections for pump light attenuation to the 2D-IR signal 'η_{ZOD} and η_{ZOD}' were calculated from the IR spectra of the samples, as described previously.[21] The calculated values are shown in **Table S3** and were not very sensitive to the uncertainty in absorption The uncertainties in η are therefore neglected (they much smaller than the uncertainty in lineshape factor *B*).

Orientation effects: Although single crystals of ZSM-5 show an anisotropic distribution of acid site orientations,[22] as for the case of molecules in solution, the pressed pellets of sub-micron sized crystals (and acid site / nest transition dipole vectors) are assumed to be randomly oriented.

Measurements (A)-(H) in **Table S1** were conducted in a spectroscopy cell where the sample was placed over the aperture of a heater block (Linkam cell). Some of the 2D-IR spectra collected for this paper were for free-standing pellets, which results in a temperature gradient across the sample placed over the aperture, and for the 2D-IR, higher levels of laser heating, both factors lowering the accuracy of IR and 2D-IR ratioing.[17] The spectroscopy measurements specifically for determining a concentration ratio were therefore conducted with the sample held between two thin (0.5 mm thickness) pieces of CaF_2 and mounted on the heating block. This reduces the temperature gradient across the aperture, giving a more accurate IR spectrum.

The IR and 2D-IR measurements, though performed on the same sample are conducted separately, with the IR beam probing an area of sample ~ 2 mm in area and the 2D-IR lasers ~ 50 μm. Variations in pellet thickness sometimes occurring during the pellet pressing process might therefore degrade the comparison. This could explain why sample (D) in table 2 appears to be an outlier (though it does not explain (H)). It may be possible in future to use the 2D-IR probe beam to measure the IR absorption, eliminating this problem.

16. Bibliography

(1) Engelhardt G; Michel D. *High Resolution Solid State NMR of Silicates and Zeolites*; John Wiley & Sons, Ltd, 1988. https://doi.org/https://doi.org/10.1002/bbpc.198800267.

(2) Medeiros-Costa, I. C.; Dib, E.; Nesterenko, N.; Dath, J. P.; Gilson, J. P.; Mintova, S. Silanol Defect Engineering and Healing in Zeolites: Opportunities to Fine-Tune Their Properties and Performances. *Chem Soc Rev* **2021**, *50* (19), 11156–11179. https://doi.org/10.1039/d1cs00395j.

(3) Sarv, P.; Wichterlová, B.; Čejka, J. Multinuclear MQMAS NMR Study of NH4/Na-Ferrierites. *J Phys Chem B* **1998**, *102* (8), 1372–1378. https://doi.org/10.1021/jp973028e.

(4) Bonilla, A.; Baudouin, D.; Pérez-Ramírez, J. Desilication of Ferrierite Zeolite for Porosity Generation and Improved Effectiveness in Polyethylene Pyrolysis. *J Catal* **2009**, *265* (2), 170–180. https://doi.org/https://doi.org/10.1016/j.jcat.2009.04.022.

(5) Palčić, A.; Moldovan, S.; El Siblani, H.; Vicente, A.; Valtchev, V. Defect Sites in Zeolites: Origin and Healing. *Advanced Science* **2022**, *9* (4), 2104414. https://doi.org/https://doi.org/10.1002/advs.202104414.

(6) Pelmenschikov, A. G.; Van Wolput, J. M. C.; Janchen, J.; Van Santen, R. A. (A,B,C) Triplet of Infrared OH Bands of Zeolitic H-Complexes. *J. Phys. Chem* **1995**, *99*, 3612–3617.

(7) Pelmenschikov, A. G.; Van Santen, R. A. Water Adsorption on Zeolites: Ab-Initio Interpretation of IR Data. *Journal of Physical Chemistry* **1993**, *97* (41), 10678–10680. https://doi.org/10.1021/j100143a025.

(8) Zecchina, A.; Geobaldo, F.; Spoto, G.; Bordiga, S.; Ricchiardi, G.; Buzzoni, R.; Petrini, G. FTIR Investigation of the Formation of Neutral and Ionic Hydrogen-Bonded Complexes by Interaction of H-ZSM-5 and H-Mordenite with CH3CN and H2O: Comparison with the H-

NAFION Superacidic System. *Journal of Physical Chemistry* **1996**, *100* (41), 16584–16599. https://doi.org/10.1021/jp960433h.

(9) Bordiga, S.; Lamberti, C.; Bonino, F.; Travert, A.; Thibault-Starzyk, F. Probing Zeolites by Vibrational Spectroscopies. *Chemical Society Reviews*. The Royal Society of Chemistry October 5, 2015, pp 7262–7341. https://doi.org/10.1039/c5cs00396b.

(10) Hack, J. H.; Chen, Y.; Lewis, N. H. C.; Kung, H. H.; Tokmakoff, A. Strong H-Bonding from Zeolite Brønsted Acid Site to Water: Origin of the Broad IR Doublet. *J Phys Chem B* **2023**, *127* (51), 11054–11063. https://doi.org/10.1021/acs.jpcb.3c06819.

(11) Chakarova, K.; Drenchev, N.; Mihaylov, M.; Nikolov, P.; Hadjiivanov, K. OH/OD Isotopic Shift Factors of Isolated and H-Bonded Surface Silanol Groups. *Journal of Physical Chemistry C* **2013**, *117* (10), 5242–5248. https://doi.org/10.1021/jp400106s.

(12) Hack, J. H.; Dombrowski, J. P.; Ma, X.; Chen, Y.; Lewis, N. H. C.; Carpenter, W. B.; Li, C.; Voth, G. A.; Kung, H. H.; Tokmakoff, A. Structural Characterization of Protonated Water Clusters Confined in HZSM-5 Zeolites. *J Am Chem Soc* **2021**, *143* (27), 10203–10213. https://doi.org/10.1021/jacs.1c03205.

(13) De Marco, L.; Carpenter, W.; Liu, H.; Biswas, R.; Bowman, J. M.; Tokmakoff, A. Differences in the Vibrational Dynamics of H2O and D2O: Observation of Symmetric and Antisymmetric Stretching Vibrations in Heavy Water. *Journal of Physical Chemistry Letters* **2016**, *7* (10), 1769–1774. https://doi.org/10.1021/acs.jpclett.6b00668.

(14) Wolfsberg, M.; Massa, A. A.; Pyper, J. W. Effect of Vibrational Anharmonicity on the Isotopic Self-Exchange Equilibria H2X+D2X=2HDX. *J Chem Phys* **1970**, *53* (8), 3138–3146. https://doi.org/10.1063/1.1674461.

(15) Dereka, B.; Lewis, N. H. C.; Zhang, Y.; Hahn, N. T.; Keim, J. H.; Snyder, S. A.; Maginn, E. J.; Tokmakoff, A. Exchange-Mediated Transport in Battery Electrolytes: Ultrafast or Ultraslow? *J Am Chem Soc* **2022**, *2022*, 8591–8604. https://doi.org/10.1021/jacs.2c00154.

(16) Hamm, P.; Zanni, M. *Concepts and Methods of 2D Infrared Spectroscopy*; Cambridge University Press, 2011; Vol. 9781107000. https://doi.org/10.1017/CBO9780511675935.

(17) Donaldson, P. M.; Howe, R. F.; Hawkins, A. P.; Towrie, M.; Greetham, G. M. Ultrafast 2D-IR Spectroscopy of Intensely Optically Scattering Pelleted Solid Catalysts. *J Chem Phys* **2023**, *158* (11), 114201. https://doi.org/10.1063/5.0139103.

(18) Donaldson, P. M. The 2D-IR Spectrum of Hydrogen-Bonded Silanol Groups in Pyrogenic Silica. *Journal of Chemical Physics* **2024**, *160*, 104204. https://doi.org/10.1063/5.0193551.

(19) Mihaleva, V. V; Van Santen, R. A.; Jansen, A. P. J. Quantum Chemical Calculation of Infrared Spectra of Acidic Groups in Chabazite in the Presence of Water. *J. Chem. Phys* **2004**, *120*, 9212. https://doi.org/10.1063/1.1709896.

(20) Meunier, F. C. Pitfalls and Benefits of in Situ and Operando Diffuse Reflectance FT-IR Spectroscopy (DRIFTS) Applied to Catalytic Reactions. *React Chem Eng* **2016**, *1* (2), 134–141. https://doi.org/10.1039/C5RE00018A.

(21) Donaldson, P. M. Spectrophotometric Concentration Analysis without Molar Absorption Coefficients by 2D-IR and FT-IR Spectroscopy. *Anal Chem* **2022**, *94* (51), 17988–17999.

(22) Martinelli, A.; Creci, S.; Vavra, S.; Carlsson, P. A.; Skoglundh, M. Local Anisotropy in Single Crystals of Zeotypes with the MFI Framework Structure Evidenced by Polarised Raman Spectroscopy. *Physical Chemistry Chemical Physics* **2020**, *22* (3), 1640–1654. https://doi.org/10.1039/c9cp06199a.
